# Synthetic Preparation of the Macrocyclolipopeptide Dysoxylactam A for Potent P‑glycoprotein Inhibition

**DOI:** 10.1021/acs.joc.5c01765

**Published:** 2025-10-16

**Authors:** Petros Danielsen Siapkaras, Karoline Hanssen, Eirik Johansson Solum, Marius Aursnes

**Affiliations:** a Faculty of Chemistry, Biotechnology and Food Science, Norwegian University of Life Sciences, P.O. Box 5003, Ås NO-1433, Norway; b Department of Chemistry, Faculty of Natural Sciences, 8018 Norwegian University of Science and Technology, Trondheim NO-7491, Norway; c Faculty of Nursing and Health Sciences, Nord University, Bodø NO-8049, Norway

## Abstract

In 2019, the cyclolipopeptide dysoxylactam A was isolated and reported to be a potent inhibitor of the drug efflux pump P-glycoprotein and demonstrated the ability to reverse multidrug resistance in cancer cell lines. Herein, we report a reliable and flexible route toward dysoxylactam A, which features key transformations such as the Paterson 1,2-*anti* aldol reaction, an sp^3^-sp^3^ Fu–Suzuki coupling, asymmetric allylation, and the Corey–Nicolaou macrolactonization. All the data obtained on the synthesized natural product matched those of the authentic material.

## Graphical abstract

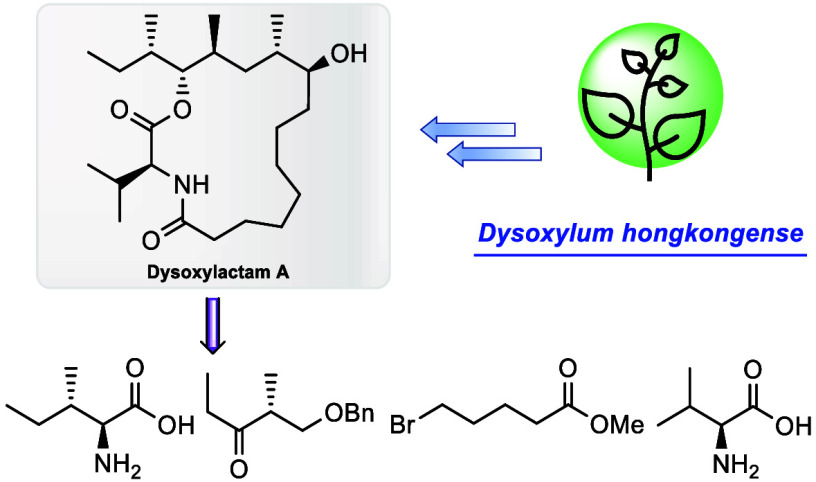

## Introduction

The bark and leaves of the South Chinese plant *Dysoxylum hongkongense* (*D. hongkongense*) have a storied legacy of use in traditional medicine, and the plant has been extensively investigated for biologically and structurally interesting compounds.^1^ In 2019, the cyclolipopeptide dysoxylactam A (**1**) was reported isolated from the bark of *D. hongkongense*.^2^ The compound was found to be an effective inhibitor of the drug efflux pump P-glycoprotein (Pgp) in a dose-dependent manner and demonstrated the ability to effectively reverse multidrug resistance (MDR) in cancer cells. Overexpression of efflux pumps in tumors is often linked to MDR in chemotherapy, and Pgp is one of the most common drug transporters associated with this condition. Drug efflux pumps utilize the free energy derived from ATP hydrolysis to expel drugs from cells against their concentration gradients, thereby reducing intracellular drug accumulation to subtherapeutic levels and thus diminishing treatment efficacy.^3^ A notable feature in this context is the ability of dysoxylactam A (**1**) to reduce the outflow of several Pgp-substrate antineoplastic agents, thus increasing the bioavailability and restoring the cytotoxic effects of the various anticancer drugs. Examples include adriamycin, paclitaxel, and vincristine at a noncytotoxic concentration.^2^ Furthermore, Yue and co-workers have recently developed cyclolipopeptides based on the molecular architecture of dysoxylactam A (**1**), and in the process, they obtained important insights regarding structure–activity relationship (SAR) as well as new analogues, which are both simpler in structure and more potent inhibitors of Pgp.^4^

Encouraged by the natural product’s pharmacological profile coupled with our interest in investigating the pharmacological potential of dysoxylactam A (**1**) against efflux pumps in various cell types also outside the domain of cancer research, we sought to establish an effective and reliable synthesis whereby multimilligram material could be obtained.

## Results and Discussion

The chemical structure of dysoxylactam A (**1**) is characterized by a 17-membered cyclolipopeptide made up from an unusual branched fatty acid as well as l-valine. Its atypical structure and the natural product’s interesting pharmacological properties have all likely been contributing factors to the appearance of several reported syntheses of the compound.^5–10^

An overview of the key disconnections applied to dysoxylactam A (**1**) is shown below (Scheme 1). The molecule was first taken back to the related secoacid **1b**, and then, an evaluation of the manner in which the methyl groups are arranged was undertaken. This was done both with regard to their spatial orientation as well as their relative positions on the carbon backbone.

**Scheme 1:**
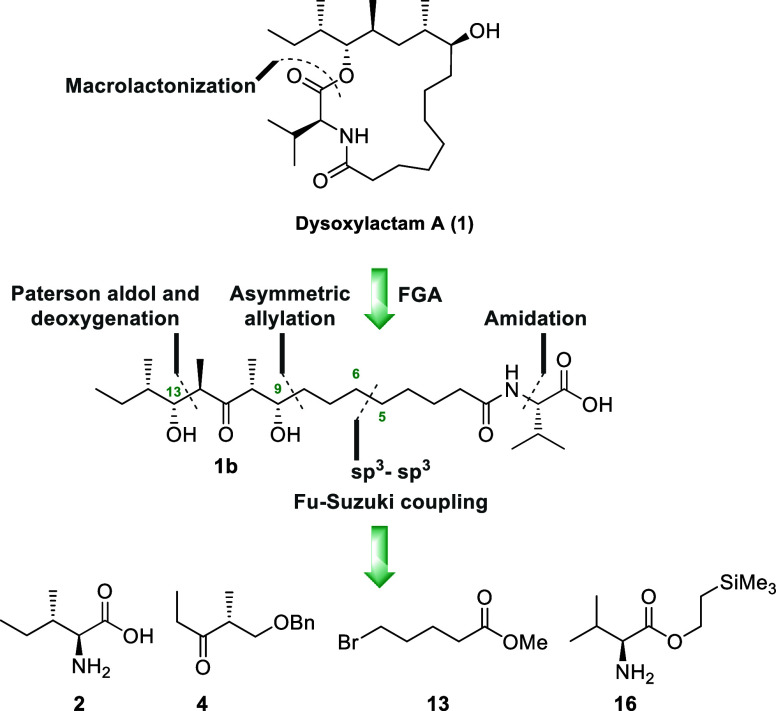
Overview of the Key Retrosynthetic Disconnections Made for Dysoxylactam A (**1**)

Taking these structural cues into consideration, combined with the presence of the two secondary alcohols situated on C13 and C9, led to the decision of attempting a Paterson 1,2-*anti*-1,4-*syn* aldol reaction,^11,12^ followed by ketone deoxygenation, to construct this specific molecular domain of **1b**. The required aldehyde (*S*)-2-methylbutanal (**3**) is available in a gram scale through the one-step Strecker degradation of l-isoleucine (**2**) using ninhydrin,^13,14^ and furthermore, the secondary alcohol at carbon C9 was planned to be built from the existing oxygenated handle present in the form of a benzyl ether in **4** ^15^ after the aldol reaction, through a sequence of deprotection, oxidation, and then an asymmetric allylation reaction. Thereafter, elongation between C5 and C6 should be possible from the terminal alkene, after its transformation to the organoboron derivative via hydroboration, using the Fu–Suzuki sp^3^–sp^3^ cross-coupling^16,17^ with methyl 5-bromopentanoate (**13**).

The synthesis was initiated in line with the plan as elaborated above, and in the 1,2-*anti*-1,4-*syn*-Paterson aldol reaction, the required *E*-enolate was formed by treatment of **4** with Cy_2_BCl followed by Et_3_N. Subsequently, freshly prepared aldehyde **3** was added at −78 °C. After oxidative workup and purification, the desired aldol product **5** was obtained in 91% yield and with a d.r. >20:1 (Scheme 2). A modified Mosher ester analysis^18,19^ experiment confirmed the absolute configuration of the secondary alcohol, and an observed ^3^ *J* coupling of 8.6 Hz verified the expected 1,2-*anti* aldol stereoisomer (Supporting Information). After TBS protection of the secondary alcohol in **5**, the next key transformation was ketone deoxygenation, and the following process proved effective in our hands: Reduction of the ketone with NaBH_4_, construction of the required xanthate, and finally Barton–McCombie deoxygenation^20^ furnished **8**. Careful reaction conditions were needed for the preparation of the xanthate via the secondary alcohol formed from the hydride reduction, as migration of the TBS group easily occurred in the 1,3-diol system in **7**. Deprotonation of secondary alcohol **7** at −78 °C using NaHMDS with excess carbon disulfide present in the reaction mixture, followed by addition of methyl iodide, solved this problem.

**Scheme 2:**
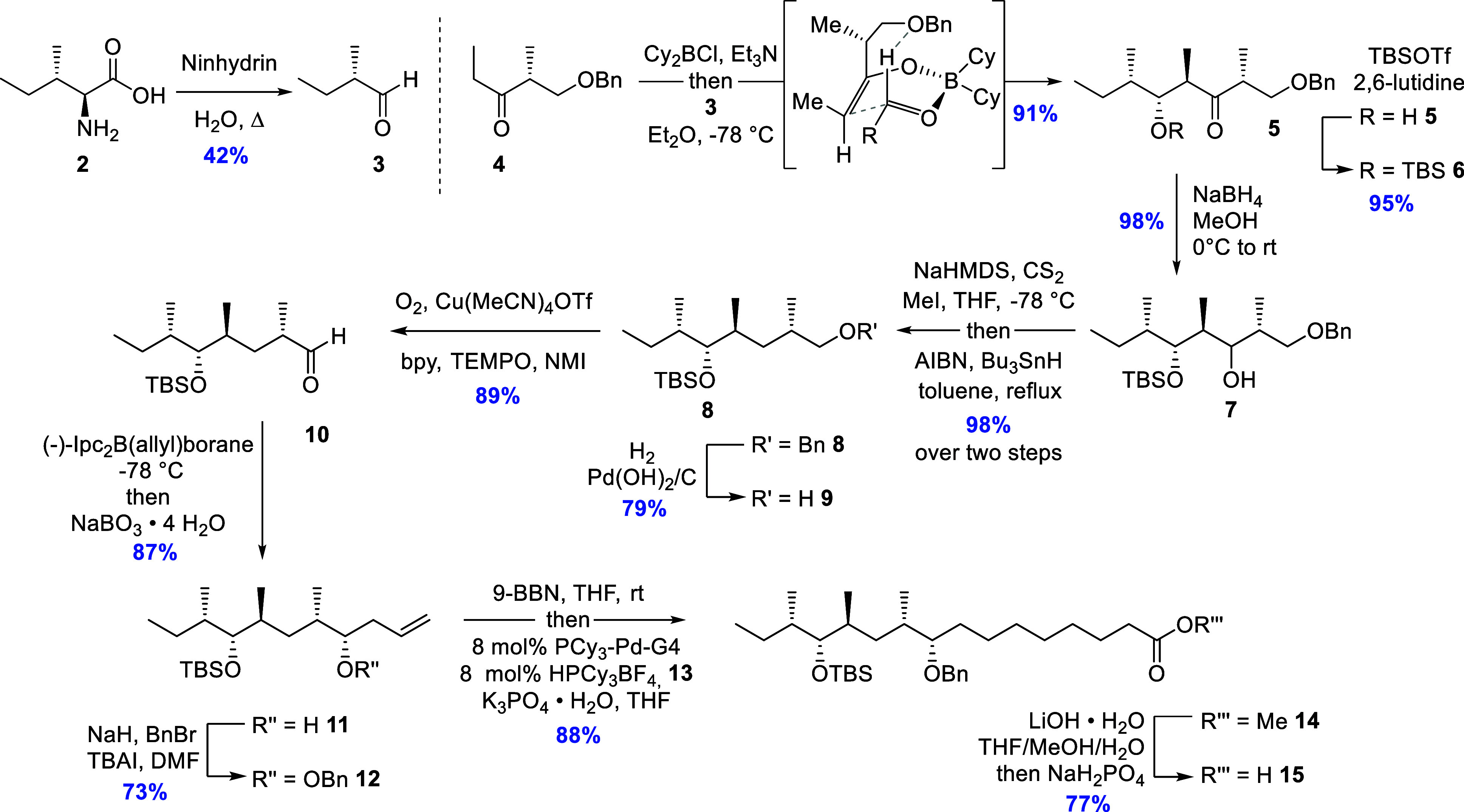
Synthetic Approach to the Branched Fatty Acid Moiety **15** of Dysoxylactam A (**1**)

The benzyl protection group in **8** was cleanly removed using Pearlman’s catalyst in a hydrogen atmosphere, and the resulting primary alcohol **9** was oxidized to the corresponding aldehyde **10** by employing the Hoover–Stahl copper-catalyzed oxidation protocol^21^ with oxygen as the stochiometric oxidant to give **10** in 89% yield. Following a protocol from Curran et al., (−)-Ipc_2_B­(allyl)­borane was made *in situ* from (−)-DIP-Cl and allylmagnesium bromide,^22^ and then, the resulting solution was added directly to aldehyde **10**, leading to the formation of allylic alcohol **11** in 87% yield and with a d.r. >20:1. A Mosher ester analysis experiment was conducted in order to ascertain the absolute configuration of the newly formed carbinol atom (Supporting Information). Next followed benzylation of the secondary alcohol in **11**, affording **12**, and then, hydroboration of the terminal alkene present in **12** was achieved by stirring with 9-BBN-H in THF overnight. Employing a constellation of the Buchwald fourth generation precatalyst PCy_3_-Pd-G4, HPCy_3_·BF_4_ as an additional source of ligand, and K_3_PO_4_·H_2_O as the base, the corresponding organoboron derivative of **12** was smoothly coupled with methyl 5-bromopentanoate (**13**) at room temperature to furnish **14** in 88% overall yield.^23^ It was found necessary to use 8 mol % precatalyst and ligand loading to effect complete union of the coupling partners in this case. Saponification using lithium hydroxide and acidic workup provided the branched fatty acid **15** in 77% yield.

The free acid **15** was then taken forward in an amidation with TMSE-protected l-valine **16**,^24^ using HATU^25,26^ as the coupling reagent, which afforded **17** in 94% yield (Scheme 3). At this stage, the two silyl-based protection groups needed to be removed prior to annulation, and this had been planned to be executed in one step. Surprisingly, however, none of the attempted reaction conditions succeeded in achieving this goal, such as TBAF, TASF, DAST, and HF·pyridine, and a two-step approach was therefore used. First, treatment with HCl generated from acetic chloride in methanol excised the *tert*-butyldimethylsilyl ether and then TBAF effectively removed the TMSE protection group, yielding **19**. For the penultimate step, the Yamaguchi protocol^27^ delivered **20** in only 11% yield. Interestingly, Gangathade successfully employed this method to obtain the desired macrolactone in 74% yield,^8^ albeit with a TBDPS instead of a benzyl-protecting group on the alcohol situated on C9. Efforts to improve upon this yield through modifications of the reaction conditions were unsuccessful in our hands, and the attention therefore turned to other macrolactonization procedures. The most successful was the Corey–Nicolaou macrolactonization^28^ when used in combination with the Gerlach–Thalmann modification,^29^ which involves the addition of silver perchlorate to activate the 2-pyridinethiol ester. This approach furnished **20** in 63% yield. The last step was the removal of the benzyl-protecting group, and this was achieved using catalytic amounts of Pd/C in a hydrogen atmosphere, as previously reported,^6,10^ giving the natural product dysoxylactam A (**1**) in 87% yield.

**Scheme 3:**
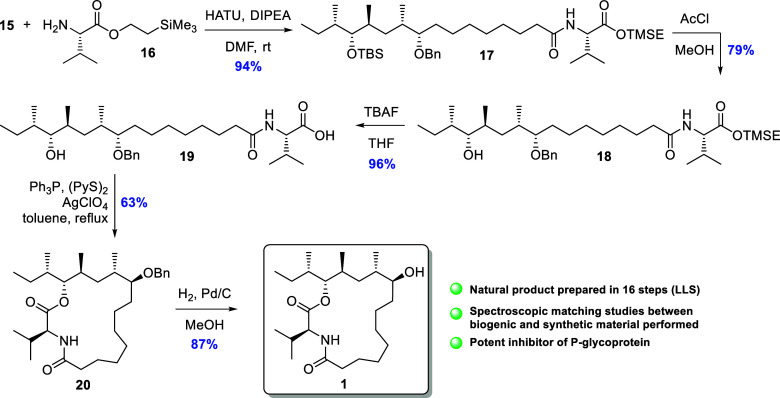
HATU-Promoted Amidation, Macrolactonization, and Finally Deprotection to Furnish Dysoxylactam A (**1**)

Upon completion of the project, the obtained NMR spectra of dysoxylactam A (**1**) were compared to the original NMR data from the isolation and characterization work, which had been kindly provided by Professors Lou and Yue at the State Key Laboratory of Drug Research, Shanghai Institute of Materia Medica, Chinese Academy of Sciences, People’s Republic of China, and this examination demonstrated a clear match (Supporting Information). Furthermore, the observed specific rotation value [α]_D_^25^ −12.5 (*c* 1.00, CHCl_3_) was in good agreement both in magnitude and sign with regard to published values.^5–10^

## Conclusions

In summary, dysoxylactam A (**1**) has been prepared in 16 steps (LLS) from known (*S*)-2-methylbutanal (**3**) and aldol reagent **4** in 10% overall yield. Key to the strategy was the realization that the Patterson protocol combined with the aldehyde generated from the Strecker degradation of l-isoleucine would enable swift establishment of the C14, C12, and C10 methyl groups, as well as the secondary alcohol at C13, with a high degree of stereochemical control and fidelity. Additionally, by this approach, the oxygenated handle positioned at the C9 position was also correctly placed for further elaboration. From there on, the next major transformations included an asymmetric allylation, Fu–Suzuki sp^3^–sp^3^ coupling, and the Corey–Nicolaou macrolactonization. The overall route has proven proficient for obtaining multimilligrams of the natural product, and over 100 mg has been made in our laboratory to this date, enabling biological investigations in various directions to be initiated.

## Experimental Section

### General Experimental Procedures

Optical rotations were measured by using a 0.7 mL cell with a 1.0 dm path length on an Anton Paar MCP 100 polarimeter. Melting points were determined on a Stuart-SMP10 melting point apparatus using open-glass capillaries and are reported as uncorrected. NMR spectra were recorded on a Bruker Ascend 400 MHz for ^1^H NMR and at 100 for ^13^C NMR. Spectra are referenced relative to the central residual protium solvent resonance in ^1^H NMR (CDCl_3_ δH = 7.26 and pyrdine-*d*_5_ δH = 8.74) and the central carbon solvent resonance in ^13^C NMR (CDCl_3_ δC = 77.16 and pyridine-*d*_5_ δC = 135.91). LC–HRMS/MS analyses were performed by using a Vanquish Horizon UHPLC instrument (Thermo Fisher Scientific, Waltham, MA, USA) connected to a Q Exactive mass spectrometer (Thermo Fisher Scientific), equipped with a HESI-II heated electrospray interface. Chromatography was performed on an XSelect CSH C18 column (30 mm × 2.1 mm i.d., 2.5 μm; Waters, Milford, MA, USA) eluted (1.0 mL/min) with a linear gradient of A, water, and B, MeCN, each of which contained 0.1% (v/v) formic acid. The gradient was 5% B for the first 0.5 min, then to 98% B at 2.5 min (held until 4.0 min), and to 5% B at 4.1 min followed by 0.9 min of equilibration (total run time of 5.0 min). Injection volumes were 1 or 2 μL. The mass spectrometer was operated with alternating full-scan (FS) positive and negative data acquisition modes, with spray voltages of +3.3 and −2.8 kV, a capillary at 250 °C, sheath and auxiliary gas of 35 and 10 units, respectively, a probe heater at 300 °C, and the S-lens RF level set to 50. The mass spectrometer was set to scan *m*/*z* 200–800, set at 70,000 resolution, max IT 100 ms, and an AGC target of 1 × 10^6^. Data were processed with Xcalibur v. 4.7 (Thermo Fisher Scientific). Thin-layer chromatography was performed on silica gel 60 F254 aluminum-backed plates fabricated by Merck (Darmstadt, Germany). Flash column chromatography was performed on silica gel 60 (40–63 μm) produced by Merck (Darmstadt, Germany). Unless stated otherwise, all commercially available reagents and solvents were used in the form in which they were supplied without any further purification. All reactions were performed under a nitrogen atmosphere unless otherwise stated. The yields given are based on the isolated material. Liquid chromatography-grade solvents were purchased from Fisher Scientific (Oslo, Norway).

### (*S*)-2-Methylbutanal (**3**)

A flask containing degassed water (800 mL) was heated until 90 °C (heating mantle), and at that point, commercially available (Merck) l-isoleucine **2** (5.00 g, 38.1 mmol, 1.00 equiv) and ninhydrin (28.8 g, 162 mmol, 4.24 equiv) were added in quick succession. The reaction mixture was heated further until boiling, with vigorous stirring and a gentle stream of nitrogen, and then, a simple short-path distillation was performed until approximately half the original volume had been collected. The clear distillate was saturated with solid NaCl, and the aqueous layer was extracted with cold pentane (5 × 50 mL). The combined organic phase was dried (Na_2_SO_4_), filtrated, and concentrated *in vacuo* (cooling bath at 0 °C, pressure not lower than 200 mbar) to give aldehyde **3** (1.38 g, 16.0 mmol, 42%) as a clear oil, which was swiftly taken forward in the next reaction. A small amount of pentane was often present, but this was inconsequential to the outcome of the ensuing transformation. Additional experimentation confirmed the stereochemical integrity, see below. The spectroscopic data were in agreement with previously reported data.^13,14,30^ ^1^H NMR (400 MHz, CDCl_3_): δ 9.62 (d, *J* = 1.9 Hz, 1H), 2.28 (hd, *J* = 6.9, 2.0 Hz, 1H), 1.75 (dqd, *J* = 13.9, 7.5, 6.5 Hz, 1H), 1.50–1.37 (m, 1H), 1.09 (d, *J* = 7.0 Hz, 3H), 0.95 (t, *J* = 7.5 Hz, 3H). ^13^C NMR (100 MHz, CDCl_3_): δ 205.6, 47.9, 23.7, 13.0, 11.5.

#### Experiments Related to Stereochemical Integrity

The distillate (∼20 mL) following the Strecker degradation of l-isoleucine (250 mg, 1.91 mmol, 1.00 equiv), performed as per the procedure described above, was extracted with CH_2_Cl_2_ (3 × 8 mL) and dried (Na_2_SO_4_). The organic phase was filtrated directly into a solution of sodium borohydride (144 mg, 3.81 mmol, 2.00 equiv) in methanol (10 mL) at 0 °C and then allowed to warm up to room temperature. The reaction mixture was stirred for 1 h and then quenched by the addition of sat. aq. NH_4_Cl (∼30 mL). The organic phase was separated, and the aqueous phase was extracted with pentane (3 × 10 mL), dried (Na_2_SO_4_), filtrated, and concentrated *in vacuo* to yield (*S*)-(−)-2-methylbutanol (102 mg, 1.16 mmol, 61%) as a clear oil. Careful optical rotation experiments with commercially available (*S*)-(−)-2-methylbutanol (Fluorochem, F473128, PO15579-100003) gave the following results: commercial: [α]_D_^20^ = −5.2 (*c* = 1.0, EtOH); synthesized: [α]_D_^20^ = −5.3 (*c* = 1.0, EtOH). These experiments were conducted at the alcohol oxidation level given the high volatility of (*S*)-2-methylbutanal, which made it challenging to perform the precise measurements needed to obtain reliable data.

### (2*R*,4*R*,5*R*,6*S*)-1-(Benzyloxy)-5-hydroxy-2,4,6-trimethyloctan-3-one (**5**)

To a solution of chlorodicyclohexylborane (1 M solution in hexanes, 15.6 mL, 15.6 mmol, 1.50 equiv) in anhydrous Et_2_O (30 mL) was added triethylamine (2.62 mL, 18.8 mmol, 1.80 equiv) at 0 °C, leading to the formation of a white precipitate. A solution of **4** (2.15 g, 10.4 mmol, 1.00 equiv) in anhydrous Et_2_O (16 mL) was added to this suspension, and the reaction mixture was stirred at 0 °C for 2 h. The flask was cooled to −78 °C, and then, freshly prepared **3** (1.97 g, 22.9 mmol, 2.20 equiv) was added dropwise. The reaction mixture was stirred at this temperature for 1 h and then overnight inside a freezer at −20 °C. Subsequently, the flask was warmed to 0 °C and quenched by the addition of pH 7 buffer (60 mL) followed by Et_2_O (120 mL). The biphasic mixture was separated, and the aqueous layer was extracted with Et_2_O (3 × 120 mL). The combined organic phase was washed with brine, dried (Na_2_SO_4_), filtrated, and concentrated *in vacuo.* The resulting colorless oil was dissolved in MeOH (120 mL) and cooled to 0 °C. Next, pH 7 buffer (120 mL) was added, followed by the dropwise addition of aqueous H_2_O_2_ (30% w/w, 120 mL). The reaction mixture was stirred at 0 °C for 1.5 h, diluted with additional water (120 mL), and extracted with CH_2_Cl_2_ (4 × 120 mL). The combined organic phase was washed with brine, dried (Na_2_SO_4_), and concentrated *in vacuo*. The crude product was purified by flash column chromatography (15% EtOAc in heptane) to afford the desired aldol product **5** (2.78 g, 9.51 mmol, 91%) as a colorless oil. *R*_f_ (15% EtOAc in heptane) = 0.17; [α]_D_^20^ = −7.6 (*c* = 1.0, CHCl_3_); ^1^H NMR (400 MHz, CDCl_3_): δ 7.37–7.27 (m, 5H), 4.51 (d, *J* = 12.0 Hz, 1H), 4.46 (d, *J* = 12.0 Hz, 1H), 3.76–3.65 (m, 2H), 3.43 (dd, *J* = 8.9, 4.9 Hz, 1H), 3.15–3.04 (m, 1H), 2.86 (dq, *J* = 8.6, 7.1 Hz, 1H), 2.44 (d, *J* = 5.5 Hz, 1H), 1.51–1.38 (m, 2H), 1.35–1.24 (m, 1H), 1.05 (dd, *J* = 7.1, 1.9 Hz, 6H), 0.95–0.83 (m, 6H); ^13^C­{^1^H} NMR (100 MHz, CDCl_3_): δ 218.1, 138.0, 128.5, 127.8, 127.8, 75.4, 73.5, 72.4, 49.7, 45.7, 36.4, 27.0, 13.9, 13.4, 12.4, 12.0; HRESIMS *m*/*z* 315.1927 [M + Na]^+^ (calcd for C_18_H_28_O_3_Na, 315.1930).

### (2*R*,4*R*,5*R*,6*S*)-1-(Benzyloxy)-5-((*tert*-butyldimethylsilyl)­oxy)-2,4,6-trimethyloctan-3-one (**6**)

To a solution of **5** (6.32 g, 21.6 mmol, 1.00 equiv) in CH_2_Cl_2_ (72 mL) at 0 °C were added 2,6-lutidine (5.00 mL, 43.0 mmol, 2.00 equiv) and TBSOTf (5.95 mL, 25.9 mmol, 1.20 equiv). The resulting mixture was stirred at this temperature for 1 h. The reaction was then quenched with sat. aq. NH_4_Cl (30 mL), CH_2_Cl_2_ (80 mL) was added, and then, the two layers were separated. The aqueous layer was extracted with EtOAc (3 × 80 mL), and the combined organic phase was washed with brine, dried (Na_2_SO_4_), and concentrated *in vacuo*. The crude product was purified by flash column chromatography (SiO_2_, 5% EtOAc in heptane) to afford silyl protected compound **6** (8.38 g, 20.6 mmol, 95%) as a colorless oil. *R*_f_ (15% EtOAc in heptane) = 0.53; [α]_D_^25^ = −38.9 (*c* = 1.08, CHCl_3_); ^1^H NMR (400 MHz, CDCl_3_): δ 7.36–7.26 (m, 5H), 4.51 (d, *J* = 12.0 Hz, 1H), 4.47 (d, *J* = 12.0 Hz, 1H), 3.94 (dd, *J* = 8.0, 2.1 Hz, 1H), 3.66 (dd, *J* = 9.1, 6.8 Hz, 1H), 3.50 (dd, *J* = 9.1, 5.5 Hz, 1H), 3.02–2.86 (m, 2H), 1.49–1.37 (m, 2H), 1.22–1.12 (m, 1H), 1.08 (d, *J* = 7.0 Hz, 3H), 0.96 (d, *J* = 7.1 Hz, 3H), 0.92–0.86 (m, 6H), 0.85 (s, 9H), 0.06 (s, 3H), −0.07 (s, 3H); ^13^C­{^1^H} NMR (100 MHz, CDCl_3_): δ 214.4, 138.4, 128.5, 127.7, 127.6, 76.6, 73.4, 72.3, 50.0, 47.0, 38.7, 26.6, 26.3, 18.6, 13.8, 13.4, 13.2, 12.5, −3.6, −4.4; HRESIMS *m*/*z* 429.2792 [M + Na]^+^ (calcd for C_24_H_42_O_3_SiNa, 429.2795).

### (2*R*,4*S*,5*R*,6*S*)-1-(Benzyloxy)-5-((*tert*-butyldimethylsilyl)­oxy)-2,4,6-trimethyloctan-3-ol (**7**)

To a solution of **6** (8.38 g, 20.6 mmol, 1.00 equiv) in MeOH (100 mL) at 0 °C was added portion-wise NaBH_4_ (1.56 g, 41.2 mmol, 2.00 equiv). The resulting solution was allowed to warm up to rt and stirred for 1 h. Additional NaBH_4_ (1.56 g, 41.2 mmol, 2.00 equiv) was added at 0 °C, and the reaction mixture was warmed to rt and stirred for another 1 h. The flask was then cooled to 0 °C and quenched with sat. aq. NH_4_Cl (30 mL). EtOAc (100 mL) was added, and the two layers were separated. The aqueous layer was extracted with EtOAc (3 × 200 mL), and the combined organic phase was washed with brine, dried (Na_2_SO_4_), filtrated, and concentrated *in vacuo*. The crude product was purified by flash column chromatography (SiO_2_, 15% EtOAc in heptane) to afford **7** (8.29 g, 20.3 mmol, 98%) as a colorless oil and a diastereomeric mixture. *R*_f_ (15% EtOAc in heptane) = 0.49. [α]_D_^25^ = −3.3 (*c* = 1.3, CHCl_3_); ^1^H NMR (400 MHz, CDCl_3_): δ 7.40–7.26 (m, 5H), 4.54 (d, *J* = 12.0 Hz, 1H), 4.50 (d, *J* = 12.0 Hz, 1H), 3.74–3.67 (m, 1H), 3.66–3.53 (m, 2H), 3.52–3.41 (m, 2H), 1.99–1.86 (m, 1H)­z, 1.85–1.72 (m, 1H), 1.54–1.38 (m, 2H), 1.26–1.12 (m, 1H), 0.92 (s, 9H), 0.91–0.85 (m, 9H), 0.83–0.78 (m, 3H), 0.11 (s, 3H), 0.08 (s, 3H); ^13^C­{^1^H} NMR (100 MHz, CDCl_3_): δ 138.8, 128.5, 127.7, 127.6, 80.5, 74.9, 73.6, 73.4, 40.2, 39.9, 35.2, 26.9, 26.2, 18.4, 15.3, 14.6, 12.4, 9.3, −3.9, −4.2; HRESIMS *m*/*z* 431.2947 [M + Na]^+^ (calcd for C_24_H_44_O_3_SiNa, 431.2952).

### (((3*S*,4*R*,5*S*,7*S*)-8-(Benzyloxy)-3,5,7-trimethyloctan-4-yl)­oxy)­(*tert*-butyl)­dimethylsilane (**8**)

To a solution of **7** (3.58 g, 8.76 mmol, 1.00 equiv) in THF (30 mL) at −78 °C was added CS_2_ (4.10 mL, 68.2 mmol, 7.78 equiv). Next, NaHMDS (1 M solution in hexanes, 10.5 mL, 10.5 mmol, 1.20 equiv) was added dropwise, and the reaction mixture was stirred for 30 min (solution turned dark orange at this stage). Methyl iodide (3.95 mL, 63.4 mmol, 7.24 equiv) was added in a dropwise manner, and the reaction mixture was allowed to warm up to rt and stirred for an additional 30 min (solution turned yellow). The reaction was quenched by the addition of sat. aq. NH_4_Cl (25 mL) and EtOAc (25 mL), and the resulting biphasic mixture was separated. The aqueous layer was extracted with EtOAc (3 × 25 mL), and the combined organic phase was washed with brine, dried (Na_2_SO_4_), filtrated, and concentrated *in vacuo*. The crude product was dissolved in toluene (50 mL), and to this solution was added Bu_3_SnH (7.55 mL, 28.1 mmol, 3.20 equiv) followed by AIBN (156 mg, 0.950 mmol, 10.0 mol%). The flask was warmed to reflux (oil bath; temperature 120 °C) and stirred for 30 min. The reaction mixture was concentrated *in vacuo* and purified by flash column chromatography (SiO_2_, 2% EtOAc in heptane) to give deoxygenated compound **8** (3.40 g, 8.66 mmol, 98% over two steps) as a colorless oil. *R*_f_ (15% EtOAc in heptane) = 0.64; [α]_D_^25^ = −11.8 (*c* = 1.06, CHCl_3_); ^1^H NMR (400 MHz, CDCl_3_): δ 7.36–7.27 (m, 5H), 4.51 (s, 2H), 3.36–3.20 (m, 3H), 1.89–1.77 (m, 1H), 1.75–1.63 (m, 1H), 1.51–1.44 (m, 1H), 1.42–1.33 (m, 1H), 1.24–1.17 (m, 2H), 1.17–1.09 (m, 1H), 0.90 (s, 9H), 0.89–0.83 (m, 12H), 0.03 (s, 6H); ^13^C­{^1^H} NMR (100 MHz, CDCl_3_): δ 139.0, 128.4, 127.7, 127.5, 80.1, 77.2, 73.1, 38.0, 36.7, 34.9, 31.1, 28.0, 26.3, 18.6, 16.6, 16.4, 14.7, 12.4, −3.6, −3.7; HRESIMS *m*/*z* 393.3173 [M + H]^+^ (calcd for C_24_H_45_O_2_Si, 393.3184).

Caution! Carbon disulfide is highly flammable and toxic. Methyl iodide is toxic and flammable. Tributyltin hydride is toxic and harmful to the environment. AIBN is unstable and toxic and may explode on heating. Handle carefully and with strict laboratory safety techniques.

### (2*S*,4*S*,5*R*,6*S*)-5-((*tert*-Butyldimethylsilyl)­oxy)-2,4,6-trimethyloctan-1-ol (**9**)

To a solution of **8** (2.60 g, 6.62 mmol, 1.00 equiv) in THF (220 mL) was added Pd­(OH)_2_/C (20 wt %, 2.79 g, 3.97 mmol, 0.60 equiv), and the resulting suspension was allowed to stir under a H_2_ atmosphere at rt for 1 h. The reaction mixture was filtered through Celite, and the plug was washed with THF (2 × 200 mL). The filtrate was washed with brine, dried (Na_2_SO_4_), and concentrated *in vacuo*. The crude product was purified by flash column chromatography (SiO_2_, 15% EtOAc in heptane) to give alcohol **9** (1.59 g, 5.25 mmol, 79%) as a colorless oil. *R*_f_ (20% EtOAc in heptane) = 0.27; [α]_D_^20^ = −13 (*c* = 0.99, CH_2_Cl_2_); ^1^H NMR (400 MHz, CDCl_3_): δ 3.52–3.38 (m, 2H), 3.33 (dd, *J* = 5.1, 3.3 Hz, 1H), 1.76–1.61 (m, 2H), 1.53–1.44 (m, 1H), 1.44–1.33 (m, 1H), 1.30–1.25 (m, 1H), 1.26–1.08 (m, 3H), 0.90 (s, 9H), 0.89–0.84 (m, 12H), 0.03 (d, *J* = 1.9 Hz, 6H); ^13^C­{^1^H} NMR (100 MHz, CDCl_3_): δ 80.1, 69.6, 38.0, 36.2, 34.9, 33.5, 28.0, 26.3, 18.6, 16.5, 16.0, 14.7, 12.4, −3.6, −3.7; HRESIMS *m*/*z* 303.2714 [M + H]^+^ (calcd for C_17_H_39_O_2_Si, 303.2711).

### (2*S*,4*S*,5*R*,6*S*)-5-((*tert*-Butyldimethylsilyl)­oxy)-2,4,6-trimethyloctanal (**10**)

To a solution of **9** (1.59 g, 5.25 mmol, 1.00 equiv) in MeCN (20 mL) were added [Cu­(MeCN)_4_]­OTf (99.0 mg, 262 μmol, 5.00 mol %) and a 0.2 M solution of TEMPO (262 μmol, 5.00 mol %), bpy (262 μmol, 5.00 mol %), and NMI (525 μmol, 10.0 mol %) in MeCN (1.31 mL). The flask was equipped with an O_2_ balloon, and the dark red solution was stirred vigorously overnight at rt. The next day, the solution turned dark green, and water (20 mL) and EtOAc (20 mL) were added. The biphasic layer was separated, and the aqueous layer was extracted with EtOAc (3 × 20 mL). The combined organic phase was washed with brine, dried (Na_2_SO_4_), filtrated, and concentrated *in vacuo* to give aldehyde **10** (1.41 g, 4.69 mmol, 89%) as a light orange oil. *R*_f_ (20% EtOAc in heptane) = 0.54; [α]_D_^25^ = −3.6 (*c* = 1.1, CHCl_3_); ^1^H NMR (400 MHz, CDCl_3_): δ 9.59 (d, *J* = 1.8 Hz, 1H), 3.32 (dd, *J* = 5.0, 3.4 Hz, 1H), 2.39–2.26 (m, 1H), 1.72–1.61 (m, 1H), 1.46–1.34 (m, 4H), 1.17–1.06 (m, 1H), 1.02 (d, *J* = 6.9 Hz, 3H), 0.86 (s, 9H), 0.85–0.80 (m, 9H), −0.00 (s, 6H); ^13^C­{^1^H} NMR (100 MHz, CDCl_3_): δ 205.5, 79.8, 44.6, 38.4, 34.9, 33.2, 27.7, 26.3, 18.6, 16.7, 14.7, 13.0, 12.4, −3.6, −3.7; HRESIMS *m*/*z* 345.2463 [M + HCO_2_]^−^ (calcd for C_18_H_37_O_4_Si, 345.2467).

### (4*S*,5*S*,7*S*,8*R*,9*S*)-8-((*tert*-Butyldimethylsilyl)­oxy)-5,7,9-trimethylundec-1-en-4-ol (**11**)

To a solution of (−)-DIP-Cl (598 mg, 1.86 mmol, 1.60 equiv) in anhydrous Et_2_O (5.6 mL) was added allylmagnesium bromide (1 M solution in Et_2_O, 1.51 mL, 1.51 mmol, 1.30 equiv) in a dropwise manner at 0 °C. The reaction mixture was stirred at this temperature for 1 h, and then, the stirring was turned off. Next, the supernatant was carefully taken up in a syringe, while avoiding disturbing the settled magnesium salts, and then added in a dropwise fashion to a solution of **10** (350 mg, 1.16 mmol, 1.00 equiv) in anhydrous Et_2_O (1.2 mL) at −78 °C. The reaction mixture was stirred at this temperature for 2 h and then MeOH (0.3 mL) was added, and the reaction mixture was allowed to warm up to rt. Next, a solution of THF and H_2_O (1:1, 0.3 mL) was added, followed by NaBO_3_·4H_2_O (2.15 g, 14.0 mmol, 12.0 equiv), and the resulting suspension was stirred at rt overnight. Water (15 mL) and EtOAc (15 mL) were next added, and the organic phase was separated. The aqueous layer was extracted with EtOAc (3 × 15 mL). The combined organic phase was washed with brine, dried (Na_2_SO_4_), filtrated, and concentrated *in vacuo*. The crude product was purified by flash column chromatography (SiO_2_, 10% EtOAc in heptane) to give **11** (347 mg, 1.01 mmol, 87%) as a colorless oil. *R*_f_ (15% EtOAc in heptane) = 0.49; [α]_D_^20^ = −10 (*c* = 1.0, CH_2_Cl_2_); ^1^H NMR (400 MHz, CDCl_3_): δ 5.84 (dddd, *J* = 16.8, 10.4, 8.0, 6.3 Hz, 1H), 5.18–5.10 (m, 2H), 3.48 (dq, *J* = 8.4, 4.1 Hz, 1H), 3.33 (dd, *J* = 5.2, 3.2 Hz, 1H), 2.35–2.26 (m, 1H), 2.22–2.09 (m, 1H), 1.72–1.57 (m, 2H), 1.49 (d, *J* = 4.2 Hz, 1H), 1.42–1.31 (m, 2H), 1.21–1.10 (m, 2H), 0.90 (s, 9H), 0.89–0.82 (m, 12H), 0.04 (d, *J* = 3.7 Hz, 6H); ^13^C­{^1^H} NMR (100 MHz, CDCl_3_): δ 135.7, 118.0, 80.2, 75.4, 39.2, 38.1, 36.1, 35.6, 35.0, 28.0, 26.3, 18.6, 16.4, 14.6, 13.7, 12.4, −3.5, −3.6; HRESIMS *m*/*z* 343.3018 [M + H]^+^ (calcd for C_20_H_43_O_2_Si, 343.3027).

### (4*S*,5*S*,7*S*,8*R*,9*S*)-8-((*tert*-Butyldimethylsilyl)­oxy)-5,7,9-trimethylundec-1-en-4-ol (**12**)

To a stirred suspension of NaH (60 wt %, 176.3 mg, 4.41 mmol, 3.00 equiv) in DMF (3.40 mL) was added dropwise a solution of **11** (504 mg, 1.47 mmol, 1.00 equiv) in DMF (2.90 mL) at 0 °C. The reaction mixture was stirred at this temperature for 10 min, warmed to rt, and stirred for an additional 45 min. Benzyl bromide (525 μL, 4.41 mmol, 3.00 equiv) was added dropwise, followed by TBAI (55.4 mg, 150 μmol, 10.0 mol %), and then stirred at rt overnight. The reaction mixture was quenched with sat. aq. NH_4_Cl (3 mL), and the biphasic mixture was separated. The aqueous layer was extracted with EtOAc (3 × 10 mL). The combined organic phase was washed with brine, dried (Na_2_SO_4_), and concentrated *in vacuo*. The crude product was purified with flash column chromatography (SiO_2_, 1% EtOAc in heptane) to afford **12** (463 mg, 1.07 mmol, 73%) as a colorless oil. *R*_f_ (5% EtOAc in heptane) = 0.55; [α]_D_^20^ = −16 (*c* = 1.0, CH_2_Cl_2_); ^1^H NMR (400 MHz, CDCl_3_): δ 7.38–7.30 (m, 4H), 7.29–7.24 (m, 1H), 5.87 (ddt, *J* = 17.2, 10.1, 7.0 Hz, 1H), 5.14–5.00 (m, 2H), 4.56 (d, *J* = 11.5 Hz, 1H), 4.50 (d, *J* = 11.6 Hz, 1H), 3.32 (dd, *J* = 5.1, 3.2 Hz, 1H), 3.26 (m, 1H), 2.38–2.27 (m, 2H), 1.84–1.72 (m, 1H), 1.65 (m, 1H), 1.52–1.08 (m, 5H), 0.89 (s, 9H), 0.89–0.80 (m, 12H), 0.03 (d, *J* = 1.6 Hz, 6H); ^13^C­{^1^H} NMR (100 MHz, CDCl_3_): δ 139.2, 136.0, 128.4, 127.9, 127.5, 116.6, 83.9, 80.1, 71.9, 38.0, 35.5, 35.5, 35.2, 33.2, 28.0, 26.3, 18.6, 16.3, 14.7, 14.7, 12.4, −3.5, −3.7; HRESIMS *m*/*z* 455.3308 [M + Na]^+^ (calcd for C_27_H_48_O_2_SiNa, 455.3316).

Caution! Sodium hydride in DMF is highly reactive and thermally unstable, and this combination may lead to a runaway reaction. Handle carefully and with strict laboratory safety techniques.

### Methyl (9*S*,10*S*,12*S*,13*R*,14*S*)-9-(Benzyloxy)-13-((*tert*-butyldimethylsilyl)­oxy)-10,12,14-trimethylhexadecanoate (**14**)

A solution of 9-BBN (0.5 M in THF, 2.28 mL, 1.14 mmol, 1.20 equiv) was added dropwise to an oven-dried flask charged with **12** (411 mg, 950 μmol, 1.00 equiv). The reaction mixture was stirred at rt overnight. An undried vial was charged in open air with PCy_3_-Pd-G4 (50.5 mg, 76.0 μmol, 8.00 mol %), HPCy_3_·BF_4_ (28.0 mg, 76.0 μmol, 8.00 mol %), and K_3_PO_4_·H_2_O (284 mg, 1.23 mmol, 1.30 equiv) and then evacuated and backfilled with N_2_ (3×). Next, methyl 5-bromovalerate (**13**) (136 μL, 950 μmol, 1.00 equiv) was added to the organoborane solution, and then, the resulting solution was transferred to the vial containing the precatalyst system and base. The resulting light-yellow suspension was stirred overnight at rt. The next day, the light gray reaction mixture was diluted with Et_2_O (10 mL), and the contents of the flask were filtered through a short plug of silica. The filtrate was concentrated *in vacuo*, and the crude product was purified by flash column chromatography (SiO_2_, 0 → 2% EtOAc in heptane) to give **14** (458 mg, 834 μmol, 88%) as a colorless oil. *R*_f_ (10% EtOAc in heptane) = 0.34; [α]_D_^25^ = −16 (*c* = 0.29, CHCl_3_); ^1^H NMR (400 MHz, CDCl_3_): δ 7.36–7.27 (m, 5H), 4.53 (d, *J* = 11.5 Hz, 1H), 4.47 (d, *J* = 11.5 Hz, 1H), 3.67 (s, 3H), 3.32 (dd, *J* = 5.2, 3.1 Hz, 1H), 3.18 (dd, *J* = 7.0, 4.3 Hz, 1H), 2.30 (t, *J* = 7.6 Hz, 2H), 1.85–1.73 (m, 1H), 1.69–1.57 (m, 3H), 1.49–1.16 (m, 15H), 0.90 (s, 9H), 0.89–0.82 (m, 12H), 0.03 (d, *J* = 1.9 Hz, 6H); ^13^C­{^1^H} NMR (100 MHz, CDCl_3_): δ 174.5, 139.4, 128.4, 127.9, 127.5, 84.4, 80.2, 71.8, 51.6, 38.0, 35.3, 35.1, 34.3, 32.8, 30.5, 29.8, 29.4, 29.3, 28.1, 26.3, 26.1, 25.1, 18.6, 16.3, 15.1, 14.7, 12.4, −3.5, −3.6; HRESIMS *m*/*z* 566.4597 [M + NH_4_]^+^ (calcd for C_33_H_64_NO_4_Si, 566.4599).

### (9*S*,10*S*,12*S*,13*R*,14*S*)-9-(Benzyloxy)-13-((*tert*-butyldimethylsilyl)­oxy)-10,12,14-trimethylhexadecanoic acid (**15**)

To a solution of methyl ester **14** (473 mg, 862 μmol, 1.00 equiv) in THF/MeOH/H_2_O (2/2/1, 4.3 mL) was added LiOH·H_2_O (108 mg, 2.57 mmol, 3.00 equiv), and the resulting solution was allowed to stir overnight at rt. The reaction mixture was acidified by sat. aq. NaH_2_PO_4_ (10 mL), and then, EtOAc (10 mL) was added. The two layers were separated, and the aqueous layer was extracted with EtOAc (3 × 10 mL). The combined organic phase was dried (Na_2_SO_4_), filtrated, and concentrated *in vacuo*. The crude product was purified by flash column chromatography (SiO_2_, 15 → 40% EtOAc in heptane) to afford desired carboxylic acid **15** (356 mg, 666 μmol, 77%) as a colorless oil. *R*_f_ (20% EtOAc in heptane) = 0.28; [α]_D_^25^ = −19 (*c* = 0.21, CHCl_3_); ^1^H NMR (400 MHz, CDCl_3_): δ 7.36–7.20 (m, 5H), 4.50 (d, *J* = 11.5 Hz, 1H), 4.45 (d, *J* = 11.5 Hz, 1H), 3.30 (dd, *J* = 5.2, 3.1 Hz, 1H), 3.20–3.11 (m, 1H), 2.32 (t, *J* = 7.5 Hz, 2H), 1.84–1.71 (m, 1H), 1.68–1.54 (m, 3H), 1.50–1.06 (m, 15H), 0.87 (s, 9H), 0.85–0.79 (m, 12H), 0.00 (d, *J* = 2.0 Hz, 6H); ^13^C­{^1^H} NMR (100 MHz, CDCl_3_): δ 178.4, 139.2, 128.3, 127.8, 127.4, 84.3, 80.1, 71.7, 37.9, 35.1, 34.9, 33.8, 32.7, 30.3, 29.7, 29.2, 29.1, 27.9, 26.2, 25.9, 24.7, 18.5, 16.2, 15.0, 14.5, 12.3, −3.7, −3.8; HRESIMS *m*/*z* 557.3991 [M + Na]^+^ (calcd for C_32_H_58_O_4_SiNa, 557.3996).

### 2-(Trimethylsilyl)­ethyl ((9*S*,10*S*,12*S*,13*R*,14*S*)-9-(Benzyloxy)-13-((*tert*-butyldimethylsilyl)­oxy)-10,12,14-trimethylhexadecanoyl)-l-valinate (**17**)

To a solution of **15** (356 mg, 666 μmol, 1.00 equiv) in anhydrous DMF (3.3 mL) were added DIPEA (0.29 mL, 1.66 mmol, 2.50 equiv) and HATU (379 mg, 997 μmol, 1.50 equiv), and the solution was stirred for 15 min at rt. To the resulting brown solution was added **16** (289 mg, 1.33 mmol, 2.00 equiv) dropwise in anhydrous DMF (1 mL). The resulting reaction mixture was stirred overnight at rt and then quenched by the addition of CH_2_Cl_2_ (10 mL) and H_2_O (10 mL). The two layers were separated, and the aqueous layer was extracted with CH_2_Cl_2_ (3 × 10 mL). The combined organic phase was washed with brine, dried (Na_2_SO_4_), filtrated, and concentrated *in vacuo*. The crude product was purified by flash column chromatography (SiO_2_, 40% EtOAc in heptane) to give amide **17** (458 mg, 624 μmol, 94%) as a light-yellow oil. *R*_f_ (20% EtOAc in heptane) = 0.30; [α]_D_^25^ = −3.3 (*c* = 0.40, CHCl_3_); ^1^H NMR (400 MHz, CDCl_3_): δ 7.37–7.29 (m, 5H), 5.89 (d, *J* = 8.8 Hz, 1H), 4.58–4.45 (m, 3H), 4.25–4.18 (m, 2H), 3.32 (dd, *J* = 5.2, 3.1 Hz, 1H), 3.21–3.15 (m, 1H), 2.26–2.19 (m, 2H), 2.19–2.10 (m, 1H), 1.85–1.74 (m, 1H), 1.70–1.56 (m, 4H), 1.50–1.12 (m, 14H), 1.06–0.98 (m, 2H), 0.96–0.82 (m, 27H), 0.05 (s, 9H), 0.03 (d, *J* = 2.2 Hz, 6H); ^13^C­{^1^H} NMR (100 MHz, CDCl_3_): δ 173.1, 172.5, 139.4, 128.4, 127.9, 127.5, 84.4, 80.2, 71.8, 63.8, 57.0, 38.0, 37.0, 35.3, 35.1, 32.8, 31.5, 30.5, 29.9, 29.5, 29.4, 28.1, 26.3, 26.1, 25.9, 19.1, 18.6, 17.9, 17.6, 16.3, 15.1, 14.7, 12.4, −1.4, −3.5, −3.7; HRESIMS *m*/*z* 734.5568 [M + H]^+^ (calcd for C_42_H_80_NO_5_Si_2_, 734.5570).

### 2-(Trimethylsilyl)­ethyl ((9*S*,10*S*,12*S*,13*R*,14*S*)-9-(Benzyloxy)-13-hydroxy-10,12,14-trimethylhexadecanoyl)-l-valinate (**18**)

To a solution of **17** (400 mg, 545 μmol, 1.00 equiv) in anhydrous MeOH (3 mL) was added a 1 M solution of HCl in MeOH (freshly prepared from AcCl in MeOH, 5.45 mL, 5.45 mmol, 10 equiv) at 0 °C. The resulting solution was allowed to warm to rt and stirred for 2 h. The reaction mixture was then concentrated *in vacuo*, and the crude product was purified by flash column chromatography (SiO_2_, heptane → 20% EtOAc in heptane) to afford **18** (268 mg, 432 μmol, 79%) as a colorless oil. *R*_f_ (20% EtOAc in heptane) = 0.11; [α]_D_^25^ = +10 (*c* = 0.20, CHCl_3_); ^1^H NMR (400 MHz, CDCl_3_): δ 7.33–7.22 (m, 5H), 5.85 (d, *J* = 8.8 Hz, 1H), 4.53–4.41 (m, 3H), 4.21–4.13 (m, 2H), 3.19–3.07 (m, 2H), 2.21–2.15 (m, 2H), 2.15–2.06 (m, 1H), 1.87–1.76 (m, 1H), 1.63–1.51 (m, 4H), 1.43–1.11 (m, 14H), 1.00–0.93 (m, 2H), 0.91–0.76 (m, 18H), −0.00 (s, 9H); ^13^C­{^1^H} NMR (100 MHz, CDCl_3_): δ 173.1, 172.6, 139.3, 128.4, 128.0, 127.5, 84.6, 79.3, 72.0, 63.8, 57.0, 37.0, 36.7, 34.4, 33.6, 32.7, 31.5, 30.4, 29.8, 29.5, 29.4, 27.0, 26.1, 25.8, 19.1, 17.9, 17.6, 16.1, 15.4, 12.9, 11.9, −1.4; HRESIMS *m*/*z* 642.4516 [M + Na]^+^ (calcd for C_36_H_65_NO_5_SiNa, 642.4524).

### ((9*S*,10*S*,12*S*,13*R*,14*S*)-9-(Benzyloxy)-13-hydroxy-10,12,14-trimethylhexadecanoyl)-l-valine (**19**)

To a solution of **18** (268 mg, 432 μmol, 1.00 equiv) in THF (4.3 mL) was added dropwise TBAF (1 M solution in THF, 1.30 mL, 1.30 mmol, 3.00 equiv) at 0 °C. The reaction mixture was allowed to warm up at rt and was stirred overnight. The reaction mixture was diluted with EtOAc (10 mL), aq. HCl (1 M, 10 mL) was added, and the two layers were separated. The organic phase was washed with aq. HCl (1 M, 2 × 10 mL), brine, dried (Na_2_SO_4_), filtrated, and concentrated *in vacuo* to afford the secoacid **19** (215 mg, 0.414 mmol, 96%) as a colorless oil. *R*_f_ (1% MeOH in CH_2_Cl_2_) = 0.13; [α]_D_^25^ = +17 (*c* = 0.22, CHCl_3_); ^1^H NMR (400 MHz, CDCl_3_): δ 7.38–7.22 (m, 5H), 6.01 (d, *J* = 8.7 Hz, 1H), 4.88–4.33 (m, 3H), 3.44–2.94 (m, 2H), 2.64–2.12 (m, 3H), 2.03–1.82 (m, 1H), 1.85–1.11 (m, 18H), 1.08–0.71 (m, 18H); ^13^C­{^1^H} NMR (100 MHz, CDCl_3_): δ 174.2, 173.9, 139.0, 128.4, 128.1, 127.6, 84.7, 79.5, 71.9, 57.1, 36.7, 36.6, 33.9, 33.5, 32.5, 31.0, 30.0, 29.7, 29.0, 28.9, 27.0, 25.8, 25.7, 19.2, 17.9, 15.9, 15.8, 12.8, 11.8; HRESIMS *m*/*z* 542.3811 [M + Na]^+^ (calcd for C_31_H_53_NO_5_Na, 542.3816).

### (3*S*,13*S*,14*S*,16*S*,17*R*)-13-(Benzyloxy)-17-((*S*)-*sec*-butyl)-3-isopropyl-14,16-dimethyl-1-oxa-4-azacycloheptadecane-2,5-dione (**20**)

To a solution of secoacid **19** (97 mg, 187 μmol, 1.00 equiv) were added 2,2′-dipyridil disulfide (61.7 mg, 280 μmol, 1.50 equiv) and Ph_3_P (73.4 mg, 280 μmol, 1.50 equiv). The mixture was dissolved in anhydrous toluene (13 mL), and the resulting yellow solution was stirred at rt for 12 h. The reaction mixture was added dropwise over 12 h using a syringe pump to a refluxing solution of anhydrous AgClO_4_ (201 mg, 970 μmol, 5.20 equiv) in anhydrous toluene (140 mL). The reaction mixture was cooled to rt, filtered through Celite, and concentrated *in vacuo*. The crude product was purified by flash column chromatography (SiO_2_, 10% EtOAc in heptane) to afford **20** (58.9 mg, 117 μmol, 63%) as a white solid. *R*_f_ (15% EtOAc/heptane) = 0.17; melting point: 128–129 °C; [α]_D_^25^ = +22 (*c* = 0.70, CHCl_3_); ^1^H NMR (400 MHz, CDCl_3_): δ 7.39–7.20 (m, 5H), 6.06 (d, *J* = 8.7 Hz, 1H), 4.80 (dd, *J* = 9.3, 2.4 Hz, 1H), 4.67 (dd, *J* = 8.8, 3.6 Hz, 1H), 4.54–4.42 (m, 2H), 3.08 (dq, *J* = 6.4, 3.3 Hz, 1H), 2.37 (ddd, *J* = 14.4, 7.3, 3.5 Hz, 1H), 2.25–2.09 (m, 2H), 1.92–1.78 (m, 2H), 1.77–1.62 (m, 2H), 1.59–1.12 (m, 15H), 0.98 (d, *J* = 6.8 Hz, 3H), 0.95–0.81 (m, 15H); ^13^C­{^1^H} NMR (100 MHz, CDCl_3_): δ 173.1, 172.9, 139.4, 128.4, 127.8, 127.4, 84.8, 81.9, 71.5, 56.9, 36.4, 35.9, 34.2, 33.3, 32.5, 31.7, 29.7, 28.3, 28.3, 27.6, 27.3, 25.7, 24.9, 19.5, 17.2, 16.4, 15.6, 13.1, 12.0; HRESIMS *m*/*z* 502.3889 [M + H]^+^ (calcd for C_31_H_52_NO_4_, 502.3891).

### Dysoxylactam A (**1**)

To a solution of compound **20** (63 mg, 0.13 mmol) in MeOH (4.0 mL) was added 10% Pd/C (13 mg), and the reaction mixture was stirred for 1 h under a H_2_ atmosphere. The reaction mixture was filtered through Celite, the plug was washed with MeOH (3 × 10 mL), and the filtrate was concentrated *in vacuo*. The crude product was purified by flash column chromatography (SiO_2_, 20% EtOAc in heptane) to afford dysoxylactam A (**1**) (45 mg, 0.11 mmol, 87%) as a white solid. *R*_f_ (20% EtOAc/heptane) = 0.15; melting point: 138–139 °C; [α]_D_^25^ = −12.5 (*c* = 1.00, CHCl_3_); ^1^H NMR (400 MHz, C_5_D_5_N): δ 5.69 (d, *J* = 5.2 Hz, 1H), 5.18 (dd, *J* = 9.3, 4.8 Hz, 1H), 5.08 (dd, *J* = 8.6, 3.1 Hz, 1H), 3.74–3.65 (m, 1H), 2.61–2.45 (m, 2H), 2.40–2.27 (m, 1H), 2.13–2.01 (m, 1H), 2.01–1.88 (m, 2H), 1.87–1.78 (m, 2H), 1.74–1.53 (m, 4H), 1.48–1.21 (m, 10H), 1.15 (d, *J* = 6.7 Hz, 3H), 1.08 (d, *J* = 6.8 Hz, 3H), 1.01 (d, *J* = 3.3 Hz, 3H), 0.99 (d, *J* = 3.2 Hz, 3H), 0.94–0.90 (m, 6H); ^13^C­{^1^H} NMR (100 MHz, C_5_D_5_N): δ 173.8, 173.7, 81.6, 76.5, 57.9, 37.9, 36.4, 36.0, 35.1, 33.3, 33.1, 31.8, 28.8, 28.3, 28.1, 27.8, 26.0, 25.4, 20.2, 18.1, 16.9, 16.3, 13.9, 12.3; HRESIMS *m*/*z* 434.3239 [M + Na]^+^ (calcd for C_24_H_45_NO_4_Na, 434.3241).

## Supplementary Material



## Data Availability

The data underlying this study are available in the published article and in its Supporting Information.

## Notes

Selected experimental procedures; Mosher ester analysis data; ^1^H, ^13^C, and ^19^F NMR spectra; comparative NMR of authentic and synthetic dysoxylactam A; HRMS spectra of dysoxylactam A (PDF)
