# Supplementary material for: Synthetic Preparation of the Macrocyclolipopeptide Dysoxylactam A for Potent P‑glycoprotein Inhibition
Source: J Org Chem. 2025 Oct 16;90(43):15248–55. doi: 10.1021/acs.joc.5c01765 (PMC12584111; doi:10.1021/acs.joc.5c01765)
Supplement: Supplementary file 1 [file jo5c01765_si_001.pdf]

Supporting Information

Synthetic Preparation of the Macrocyclolipopeptide Dysoxylactam A for Potent P-glycoprotein Inhibition

Petros Danielsen Siapkaras [a], *Karoline Hanssen*[b], *Eirik Johansson Solum*[b,c*] *and Marius Aursnes*[a*]

[a] Department of Chemistry, Biotechnology and Food Science, Norwegian University of Life Sciences, P.O. Box 5003, NO-1433 Ås, Norway

[b] Department of Chemistry, Faculty of Natural Sciences, Norwegian University of Science and Technology, NO-7491 Trondheim, Norway

[c] Faculty of Nursing and Health Sciences, Nord University, NO-8049 Bodø, Norway

Contents

Experimental Procedures S2
^{1}H, ^{13}C and ^{19}F NMR-spectra S6
HRMS-spectra S31
References S33

Experimental Procedures

(3*S*,4*R*,5*R*,7*R*)-8-(Benzyloxy)-3,5,7-trimethyl-6-oxooctan-4-yl (*R*)-3,3,3-trifluoro-2-methoxy-2-phenylpropanoate (5-I)

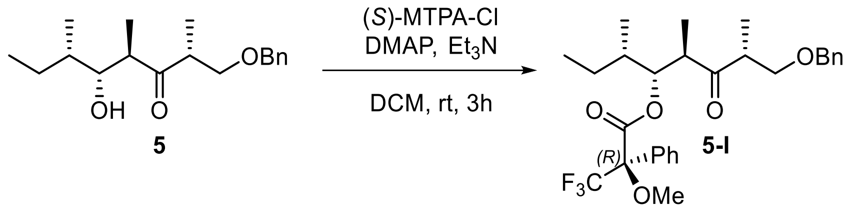

To a solution of the aldol product **5** (10.0 mg, 34.2 μmol, 1.00 equiv) in CH_2Cl_2 (0.70 mL), was added Et_3N (15.3 μL, 110 μmol, 3.20 equiv), DMAP (9.61 mg, 78.7 μmol, 2.30 equiv) and (*S*)-MTPA-Cl (14.7 μL, 78.6 μmol, 2.30 equiv) and was stirred for 3h at rt. The reaction mixture was concentrated *in vacuo* and the crude product was purified by flash column chromatography (SiO_2, 5 → 10% EtOAc in heptane) to afford **5-I** (16.0 mg, 31.5 μmol, 92%) as a colorless oil. R_f (10% EtOAc in heptane) = 0.20; $[\alpha]_D^{25}$= -10 (*c* = 1.0, $CHCl_3$); ^1H NMR (400 MHz, $CDCl_3$) δ 7.50 – 7.43 (m, 2H), 7.40 – 7.26 (m, 8H), 5.54 (dd, *J* = 9.4, 2.4 Hz, 1H), 4.49 (d, *J* = 12.0 Hz, 1H), 4.44 (d, *J* = 12.0 Hz, 1H), 3.63 (dd, *J* = 9.1, 7.8 Hz, 1H), 3.43 (d, *J* = 1.0 Hz, 3H), 3.38 (dd, *J* = 9.1, 5.1 Hz, 1H), 3.07 (dq, *J* = 9.4, 7.2 Hz, 1H), 2.88 (pd, *J* = 7.2, 5.1 Hz, 1H), 1.73 – 1.62 (m, 1H), 1.48 – 1.35 (m, 1H), 1.17 – 1.10 (m, 1H), 1.07 (d, *J* = 7.2 Hz, 3H), 0.94 (t, *J* = 7.4 Hz, 3H), 0.84 (dd, *J* = 19.7, 7.0 Hz, 6H); $^{13}C\{^1H\}$ NMR (100 MHz, $CDCl_3$) δ 212.5, 165.7, 138.4, 131.8, 129.7, 128.5, 128.4, 128.3, 127.7, 127.6, 79.2, 73.4, 71.9, 55.2, 46.8, 46.0, 36.5, 26.6, 13.2, 13.1, 13.0, 12.2; HRESIMS *m/z* 531.2319 $[M + Na]^+$ (calcd for $C_{28}H_{35}F_3O_5Na$, 531.2329).

(3*S*,4*R*,5*R*,7*R*)-8-(Benzyloxy)-3,5,7-trimethyl-6-oxooctan-4-yl (*S*)-3,3,3-trifluoro-2-methoxy-2-phenylpropanoate (5-II)

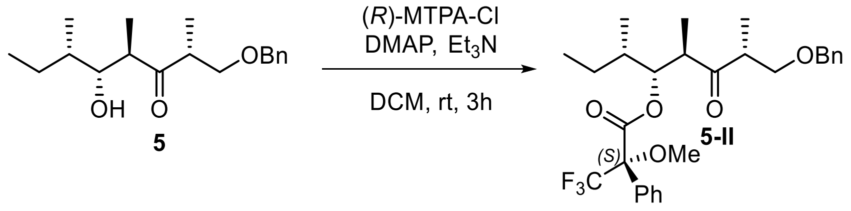

To a solution of the aldol product **5** (10.0 mg, 34.2 μmol, 1.00 equiv) in CH_2Cl_2 (0.70 mL), was added Et_3N (15.3 μL, 110 μmol, 3.20 equiv), DMAP (9.61 mg, 78.7 μmol, 2.30 equiv) and (*R*)-MTPA-Cl (14.7 μL, 78.6 μmol, 2.30 equiv) and was stirred for 3h at rt. The reaction mixture was concentrated *in vacuo* and the crude product was purified by flash column chromatography

(SiO$_2$, 5 → 10% EtOAc in heptane) to afford **5-II** (15.2 mg, 29.9 µmol, 87%) as a colorless oil. R_f (10% EtOAc in heptane) = 0.20; $[\alpha]_D^{25}$= -9.40 (*c* = 1.06, CHCl$_3$); ^{1}H NMR (400 MHz, CDCl$_3$) δ 7.53 – 7.46 (m, 2H), 7.40 – 7.26 (m, 8H), 5.56 (dd, *J* = 9.6, 2.3 Hz, 1H), 4.48 (d, *J* = 11.9 Hz, 1H), 4.42 (d, *J* = 11.9 Hz, 1H), 3.58 (dd, *J* = 9.1, 7.8 Hz, 1H), 3.50 (t, *J* = 1.3 Hz, 3H), 3.33 (dd, *J* = 9.1, 5.1 Hz, 1H), 3.07 (dq, *J* = 9.7, 7.2 Hz, 1H), 2.85 (pd, *J* = 7.2, 5.0 Hz, 1H), 1.74 – 1.63 (m, 1H), 1.52 – 1.40 (m, 1H), 1.16 (dt, *J* = 13.6, 7.6 Hz, 1H), 1.05 (d, *J* = 7.2 Hz, 3H), 1.00 – 0.90 (m, 6H), 0.74 (d, *J* = 7.1 Hz, 3H); ^{13}C{^{1}H} NMR (100 MHz, CDCl$_3$) δ 212.2, 165.4, 138.4, 132.0, 129.5, 128.5, 128.3, 127.9, 127.7, 127.6, 79.0, 73.4, 71.8, 55.5, 46.8, 46.0, 36.3, 26.9, 13.1, 13.1, 12.9, 12.3; HRESIMS *m/z* 531.2319 [M + Na]$^+$ (calcd for $C_{28}H_{35}F_3O_5Na$, 531.2329).

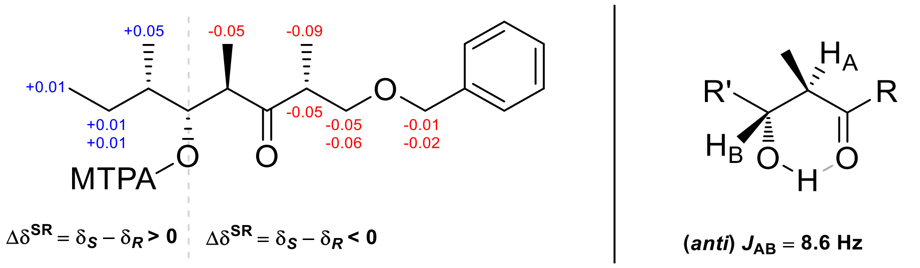

Figure S-1 Modified Mosher ester analysis of **5-I** & **5-II**: Values in blue and red are consistent with the assigned configuration. The $^3J_{AB}$-coupling constant is consistent with the *anti*-aldol product.

(4*S*,5*S*,7*S*,8*R*,9*S*)-8-((*tert*-Butyldimethylsilyl)oxy)-5,7,9-trimethylundec-1-en-4-yl (*R*)-3,3,3-trifluoro-2-methoxy-2-phenylpropanoate (11-I)

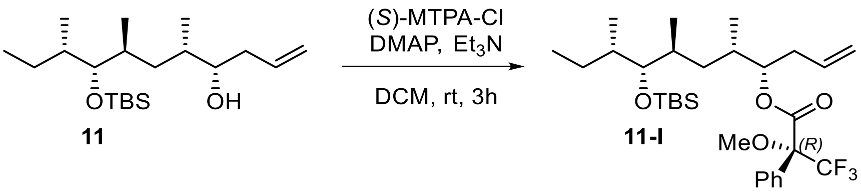

To a solution of **11** (14.5 mg, 42.3 µmol) in CH$_2$Cl$_2$ (0.8 mL), was added Et$_3$N (18.9 µL, 0.136 mmol, 3.20 equiv), DMAP (11.9 mg, 97.4 µmol, 2.30 equiv) and (*S*)-MTPA-Cl (18.2 µL, 97.3 µmol, 2.30 equiv). The reaction mixture was stirred for 3h at rt and then concentrated *in vacuo.* The crude product was purified by flash column chromatography (SiO$_2$, 2% EtOAc in heptane) to afford **11-I** (20 mg, 36 µmol, 85%) as a colorless oil. R_f (10% EtOAc in heptane) = 0.58; $[\alpha]_D^{25}$= +1.91 (*c* = 1.06, CHCl$_3$); ^{1}H NMR (400 MHz, CDCl$_3$) δ 7.60 – 7.51 (m, 2H), 7.43 – 7.33 (m, 3H), 5.76 (ddt, *J* = 17.1, 10.2, 6.9 Hz, 1H), 5.15 – 5.05 (m, 3H), 3.55 (s, 3H), 3.27 (t, *J* = 4.0 Hz, 1H), 2.43 (t, *J* = 6.9 Hz, 2H), 1.86 – 1.76 (m, 1H), 1.66 – 1.57 (m, 1H), 1.37 – 1.04

(m, 5H), 0.89 (s, 9H), 0.86 – 0.80 (m, 9H), 0.76 (d, *J* = 6.8 Hz, 3H), 0.01 (d, *J* = 4.9 Hz, 6H); $^{13}C\{^1H\}$ NMR (100 MHz, $CDCl_3$) δ 166.3, 133.8, 132.6, 129.6, 128.4, 127.6, 118.3, 80.9, 79.9, 55.6, 38.2, 35.5, 34.8, 34.7, 33.2, 27.8, 26.3, 18.6, 16.4, 15.0, 14.1, 12.3, -3.6, -3.8; ^{19}F NMR (376 MHz, $CDCl_3$) δ -71.0; HRESIMS *m/z* 581.3245 $[M + Na]^+$ (calcd for $C_{30}H_{49}F_3O_4SiNa$, 581.3244).

(4*S*,5*S*,7*S*,8*R*,9*S*)-8-((*tert*-Butyldimethylsilyl)oxy)-5,7,9-trimethylundec-1-en-4-yl (*S*)-3,3,3-trifluoro-2-methoxy-2-phenylpropanoate (11-II)

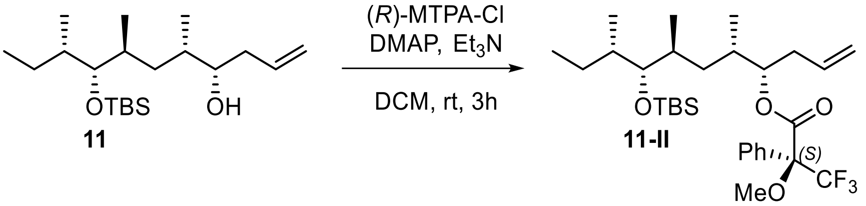

To a stirred solution of **11** (15.8 mg, 46.1 μmol) in CH_2Cl_2 (0.9 mL), was added Et_3N (20.6 μL, 0.148 mmol, 3.20 equiv), DMAP (13.0 mg, 0.106 mmol, 2.30 eq) and (*R*)-MTPA-Cl (19.8 μL, 0.11 mmol, 2.30 equiv). The reaction mixture was stirred for 3h at rt and then concentrated *in vacuo.* The crude product was purified by flash column chromatography (SiO_2, 2% EtOAc in heptane) to afford **11-II** (23 mg, 41 μmol, 89%) as a colorless oil. R_f (10% EtOAc in heptane) = 0.58; $[\alpha]_D^{25}$= -17.0 (*c* = 1.06, $CHCl_3$); ^1H NMR (400 MHz, $CDCl_3$) δ 7.58 – 7.50 (m, 2H), 7.43 – 7.35 (m, 3H), 5.66 (ddt, *J* = 17.2, 10.2, 7.1 Hz, 1H), 5.11 – 4.98 (m, 3H), 3.52 (s, 3H), 3.31 (dd, *J* = 4.6, 3.5 Hz, 1H), 2.39 – 2.33 (m, 2H), 1.90 – 1.79 (m, 1H), 1.70 – 1.60 (m, 1H), 1.46 – 1.08 (m, 5H), 0.90 (s, 9H), 0.89 – 0.81 (m, 12H), 0.03 (d, *J* = 2.3 Hz, 6H); $^{13}C\{^1H\}$ NMR (100 MHz, $CDCl_3$) δ 166.4, 133.5, 132.4, 129.6, 128.4, 127.8, 118.2, 81.0, 80.0, 55.5, 38.3, 35.4, 35.1, 34.8, 33.3, 27.8, 26.3, 18.6, 16.6, 15.0, 14.3, 14.3, 12.3, -3.6, -3.8; ^{19}F NMR (376 MHz, $CDCl_3$) δ -71.2; HRESIMS *m/z* 581.3245 $[M + Na]^+$ (calcd for $C_{30}H_{49}F_3O_4SiNa$, 581.3244).

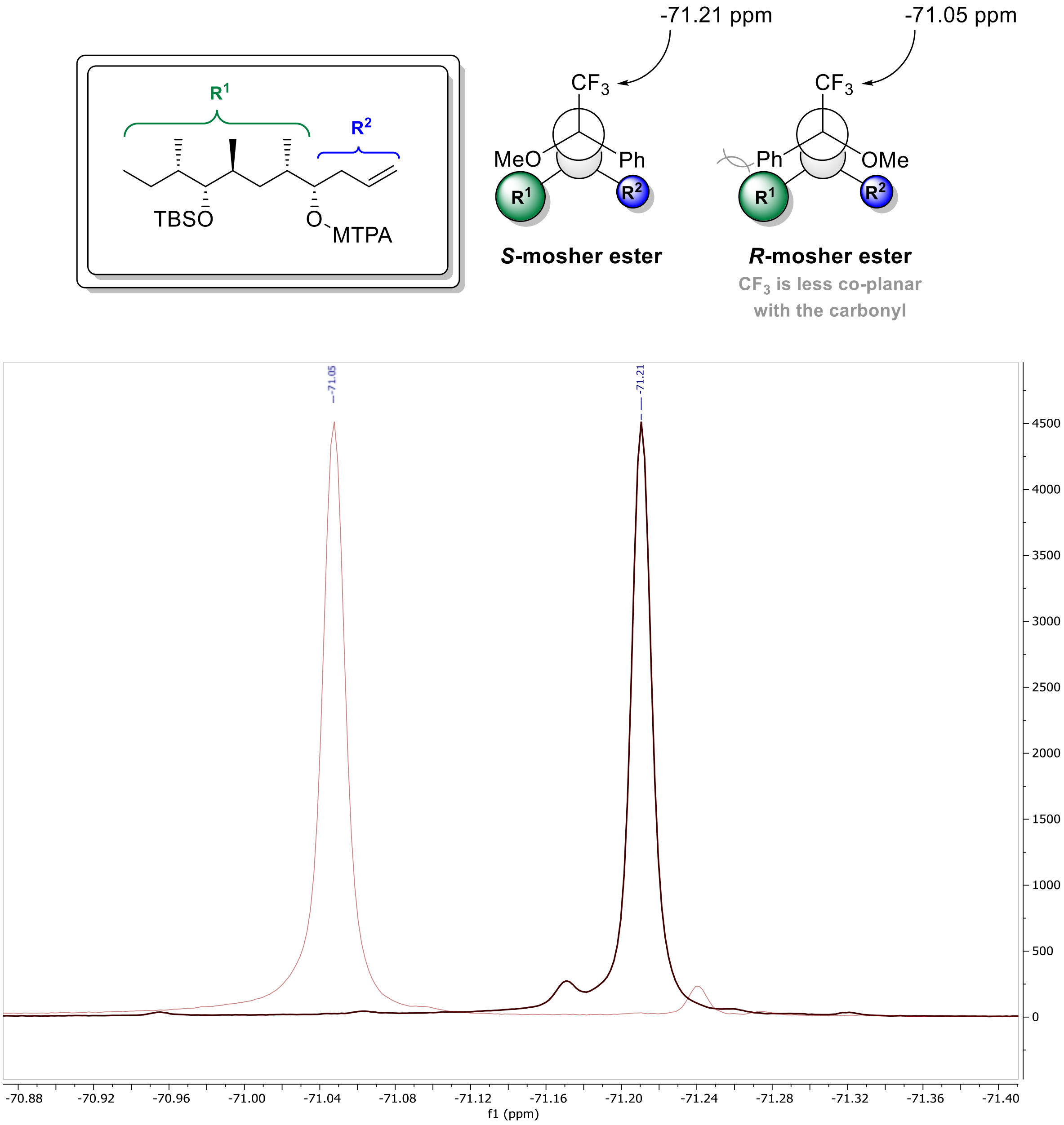

Figure S-2 Mosher ester analysis of compound **11-I** & **11-II**: The Δ between the ppm-values of the CF_3 groups in the stacked ^{19}F NMR spectrum is consistent with the assigned absolute configuration.

^{1}H, ^{13}C and ^{19}F NMR-spectra

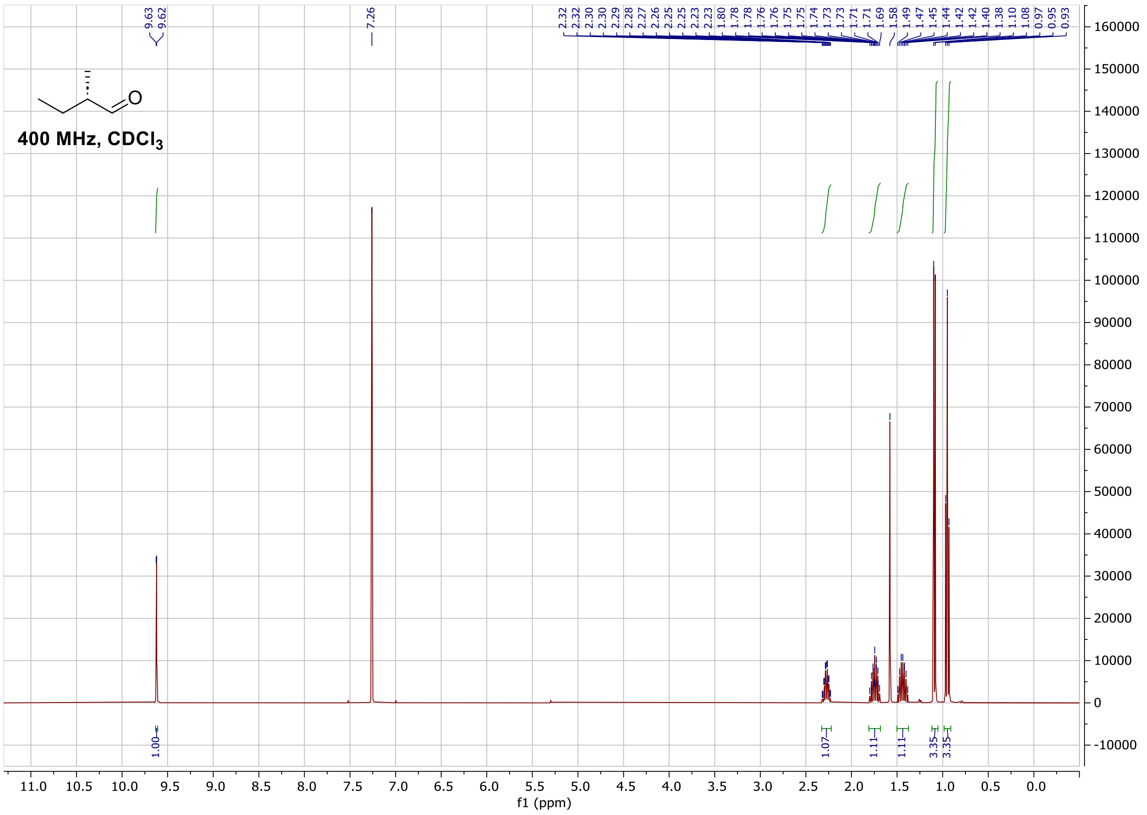

Figure S-3 ^{1}H-NMR spectrum of compound **3**.

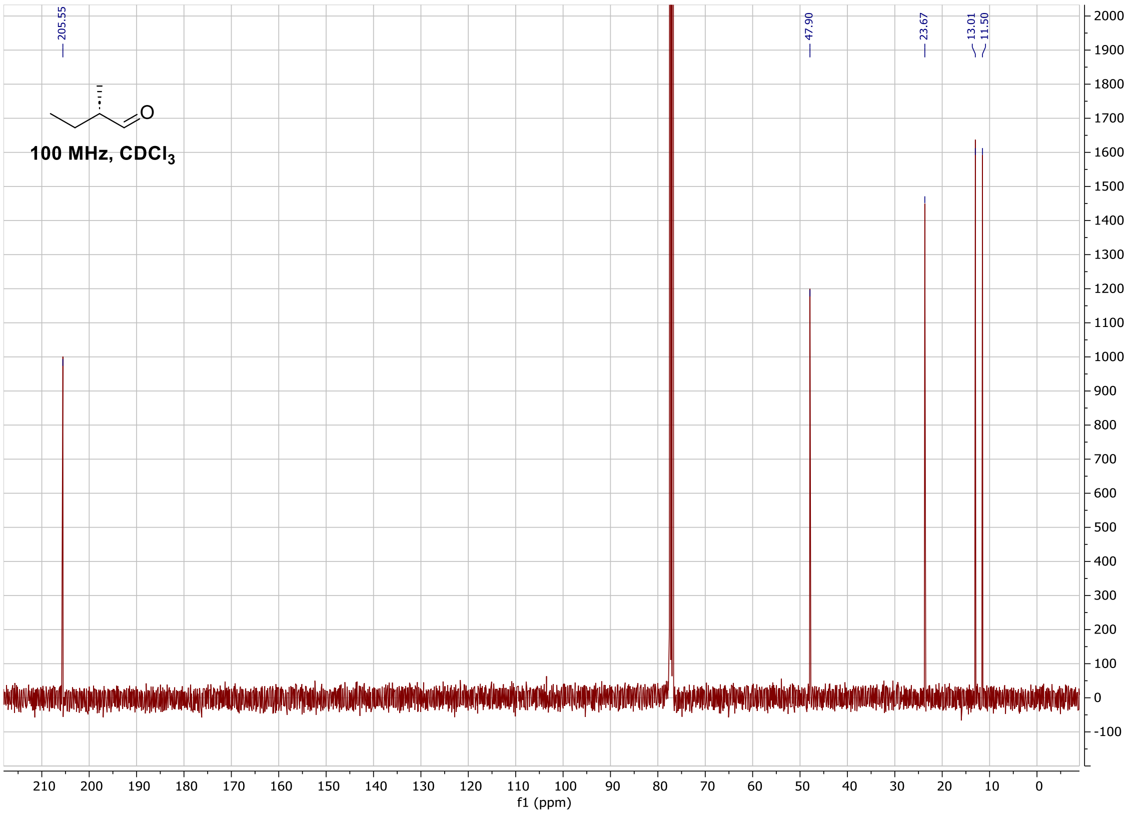

Figure S-4 ^{13}C{^{1}H} NMR spectrum of compound **3**.

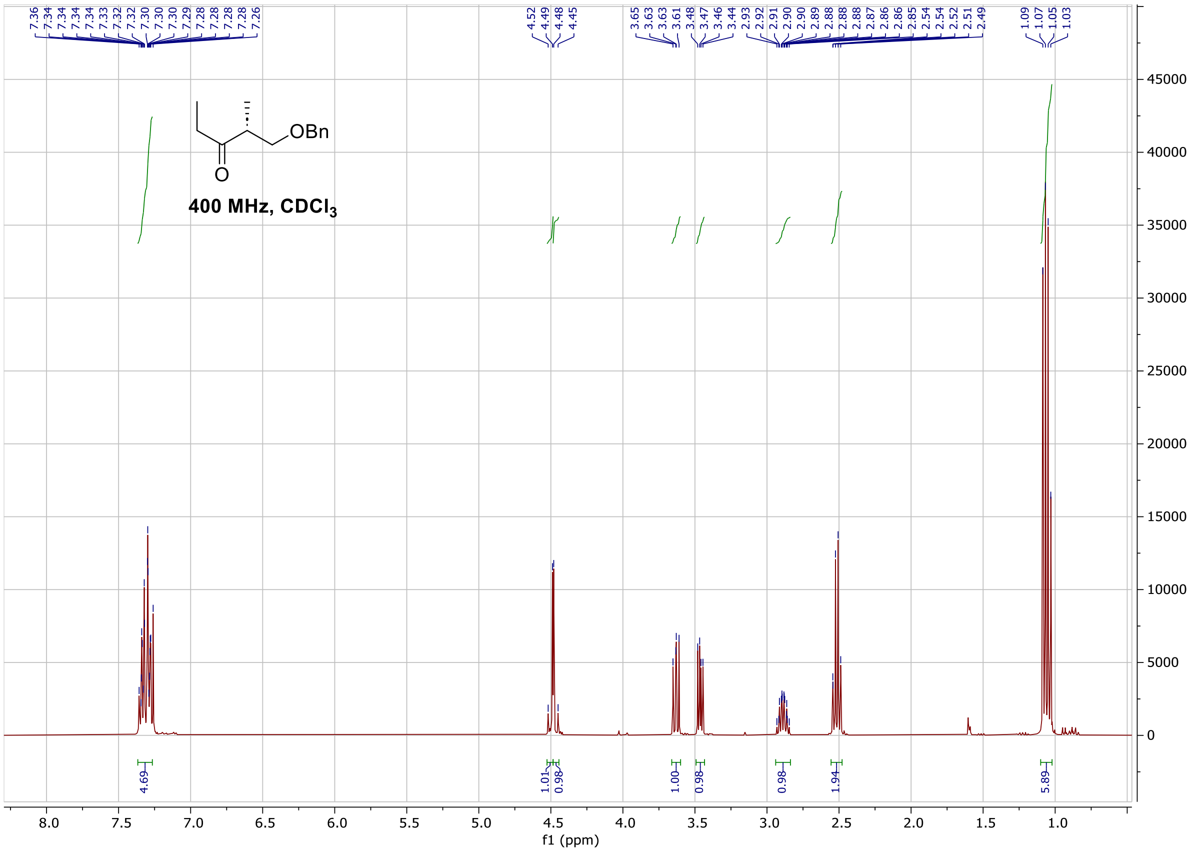

Figure S-5 ^{1}H-NMR spectrum of compound **4**.[1]

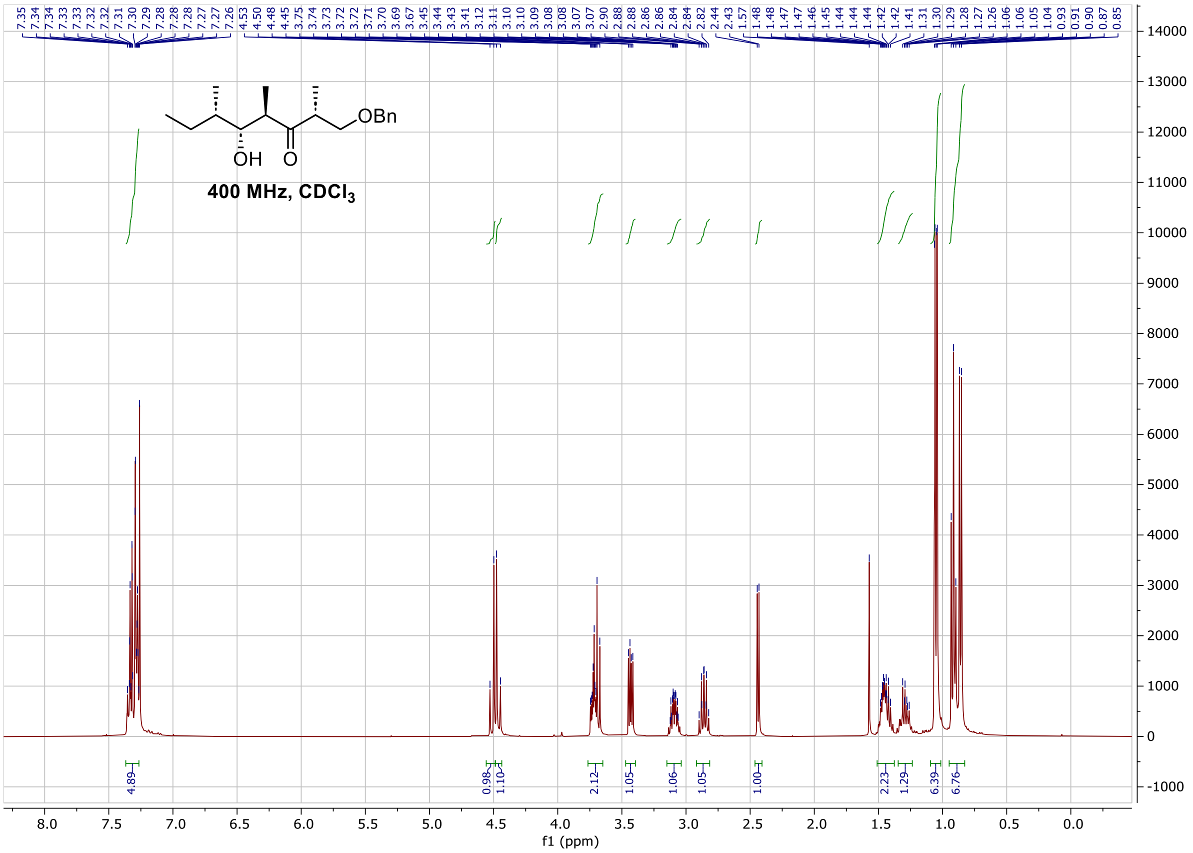

Figure S-6 ^{1}H-NMR spectrum of compound **5**.

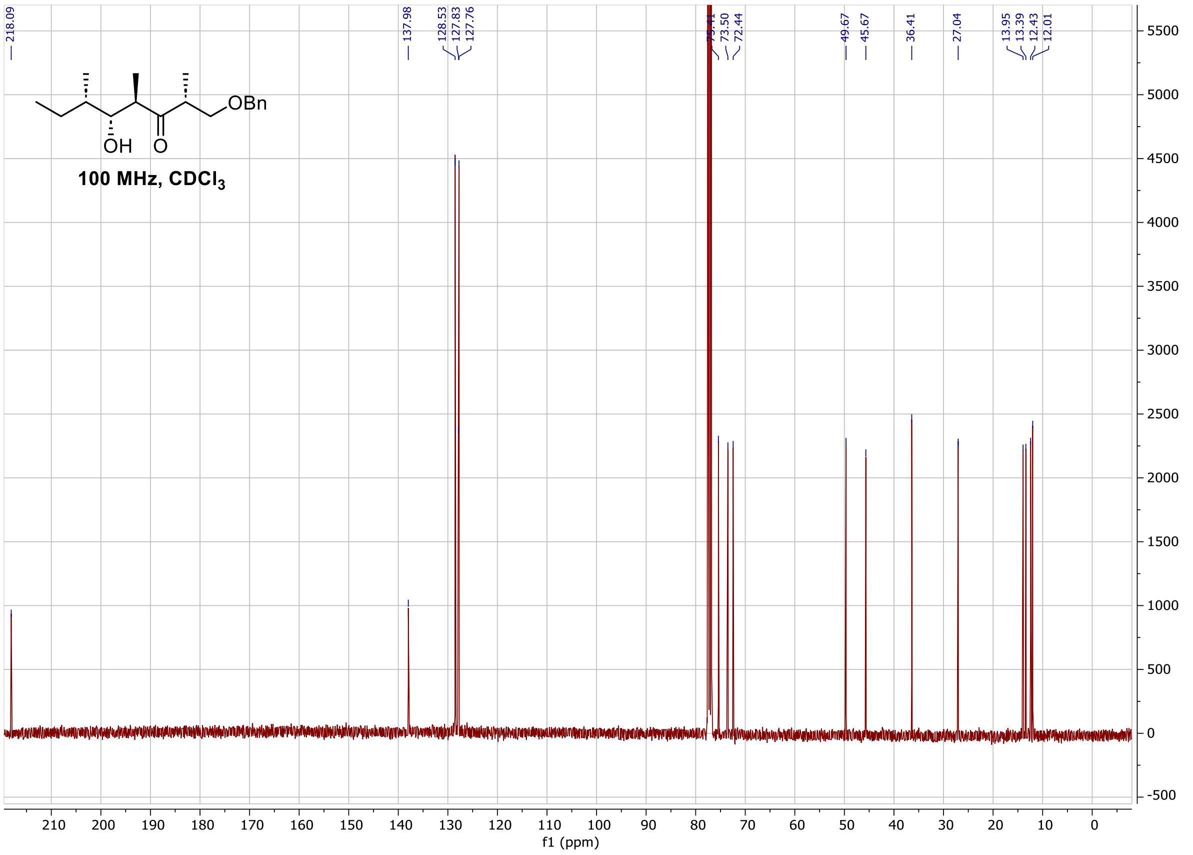

Figure S-7 $^{13}C\{^{1}H\}$ NMR spectrum of compound **5**.

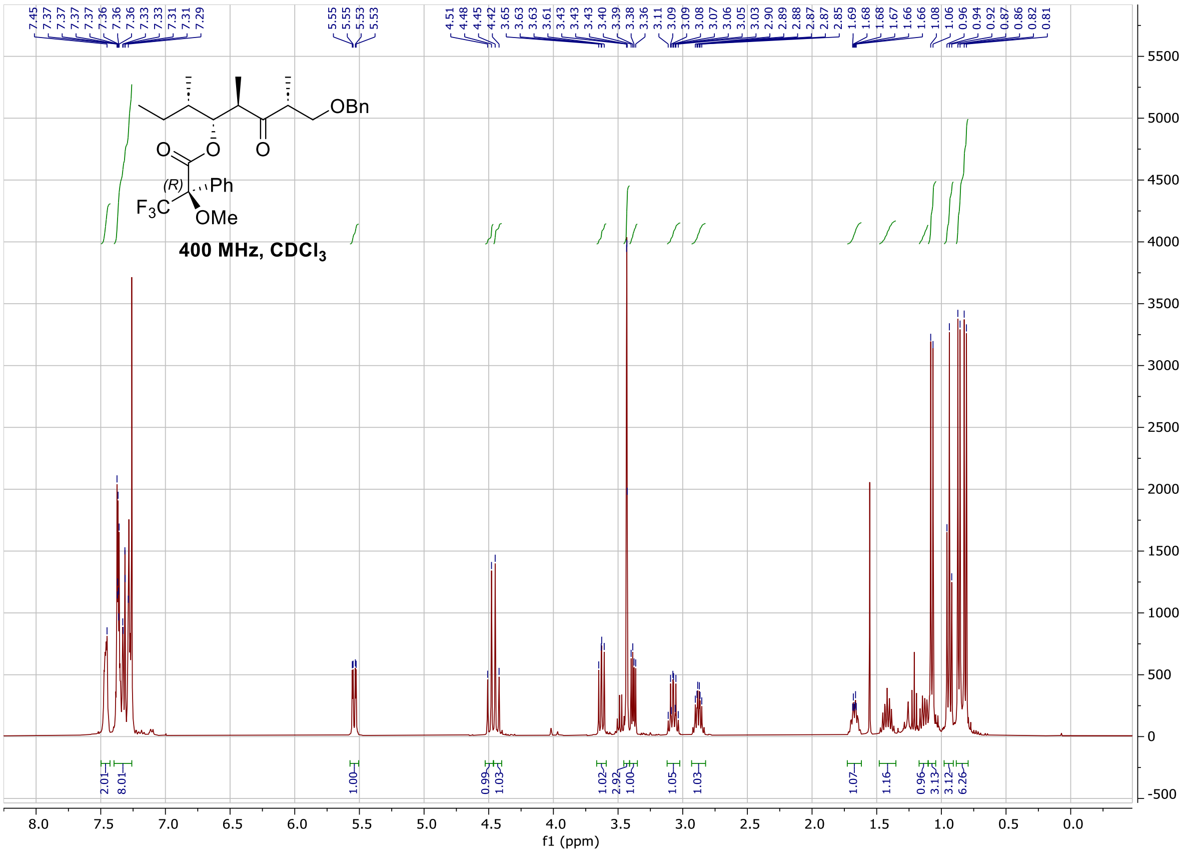

Figure S-8 ^{1}H-NMR spectrum of compound **5-I**.

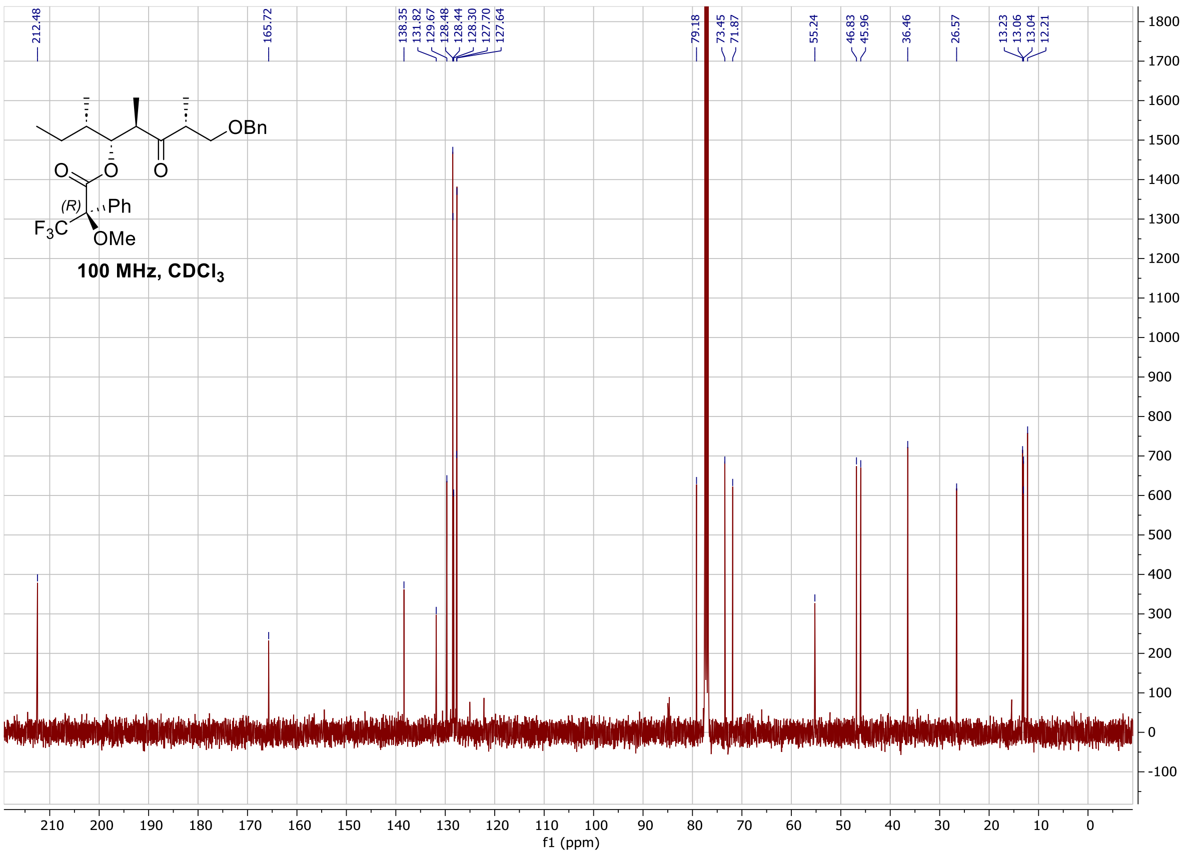

Figure S-9 $^{13}C\{^{1}H\}$ NMR spectrum of compound **5-I**.

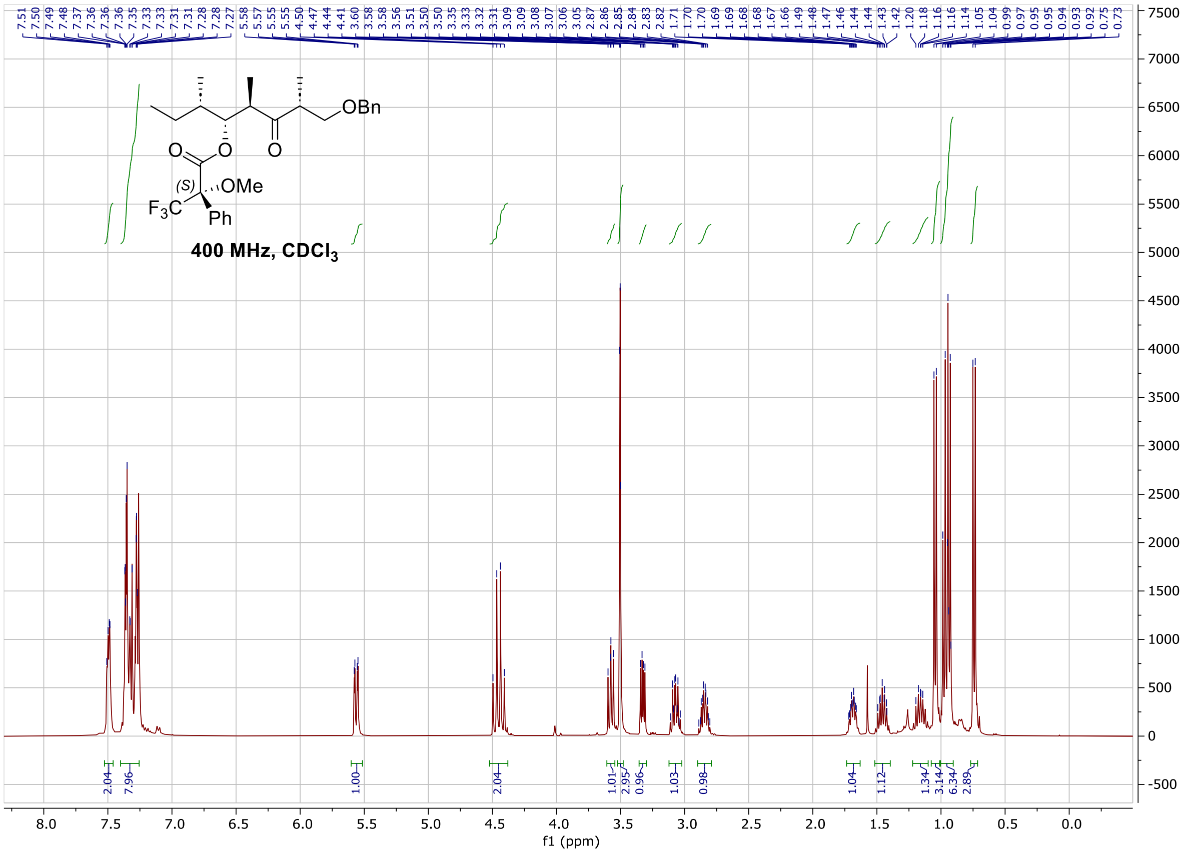

Figure S-10 ^{1}H-NMR spectrum of compound **5-II**.

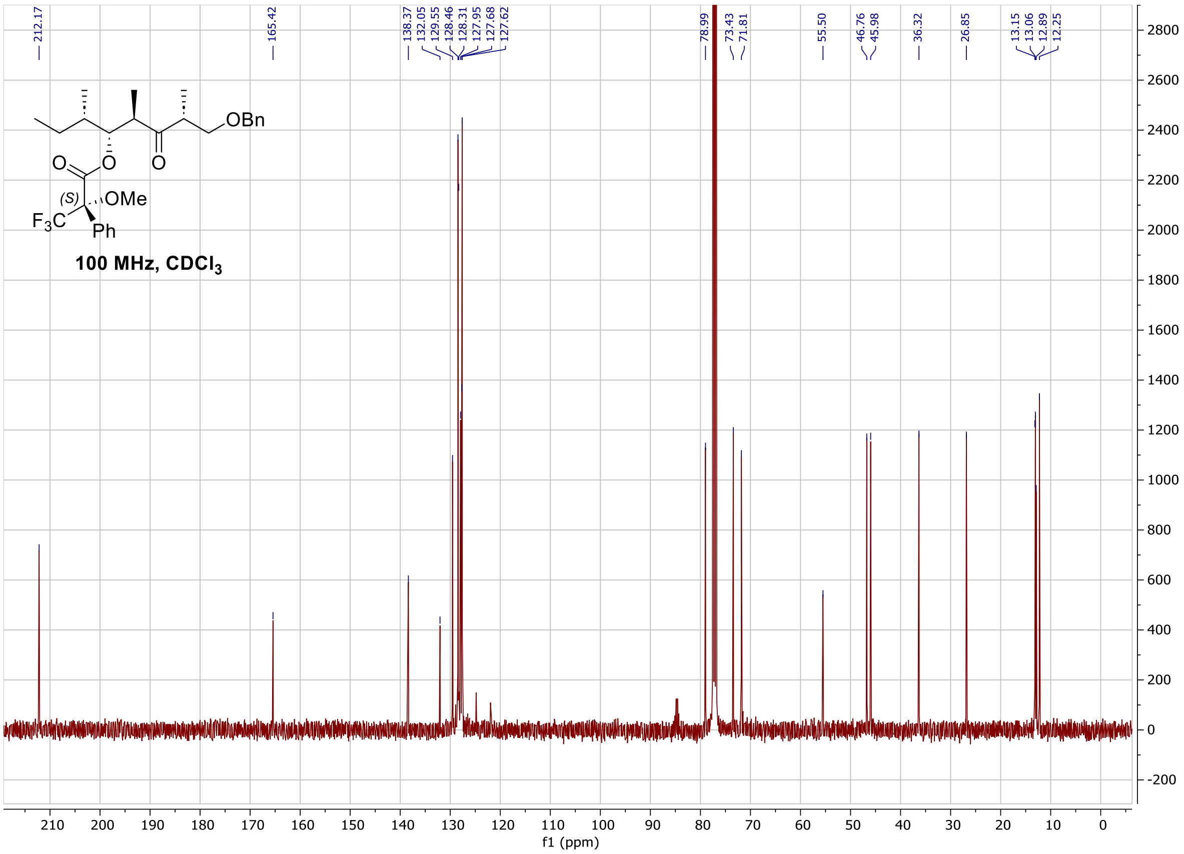

Figure S-11 $^{13}C\{^{1}H\}$ NMR spectrum of compound **5-II**.

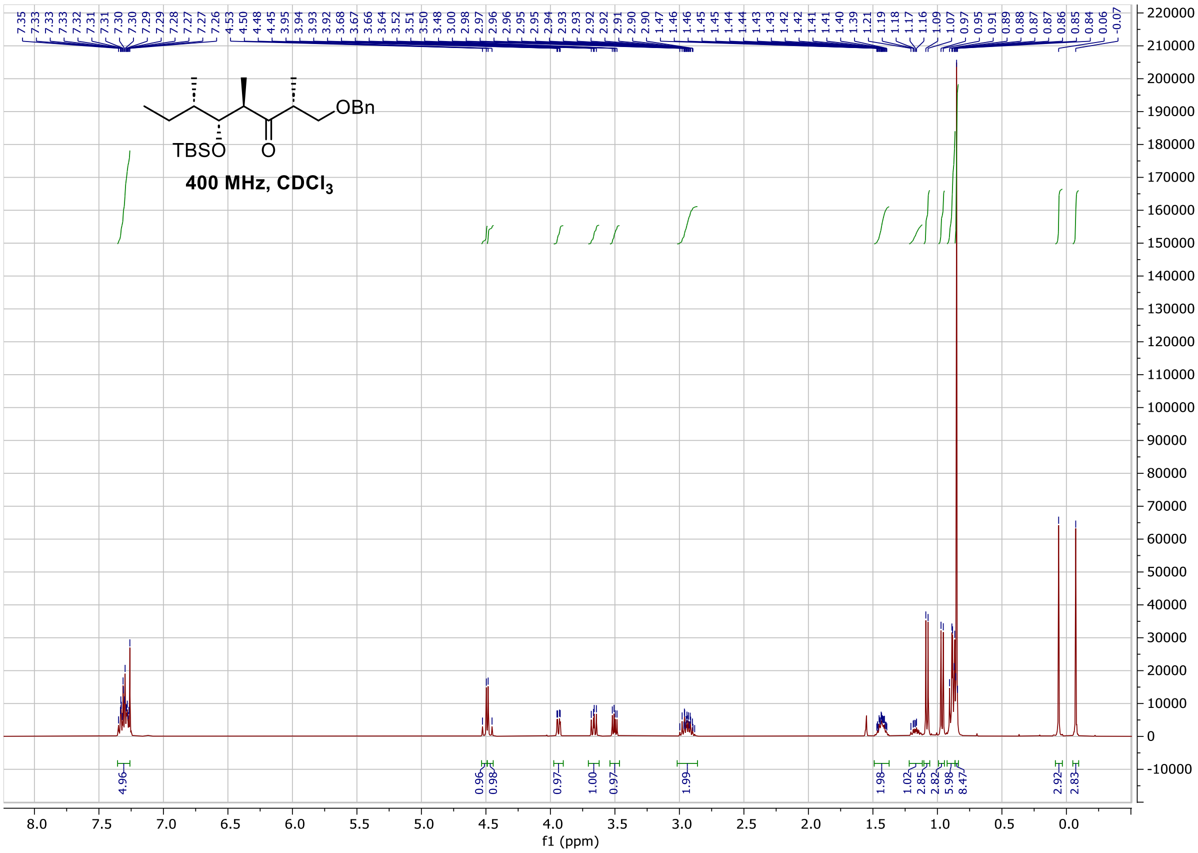

Figure S-12 ^{1}H-NMR spectrum of compound **6**.

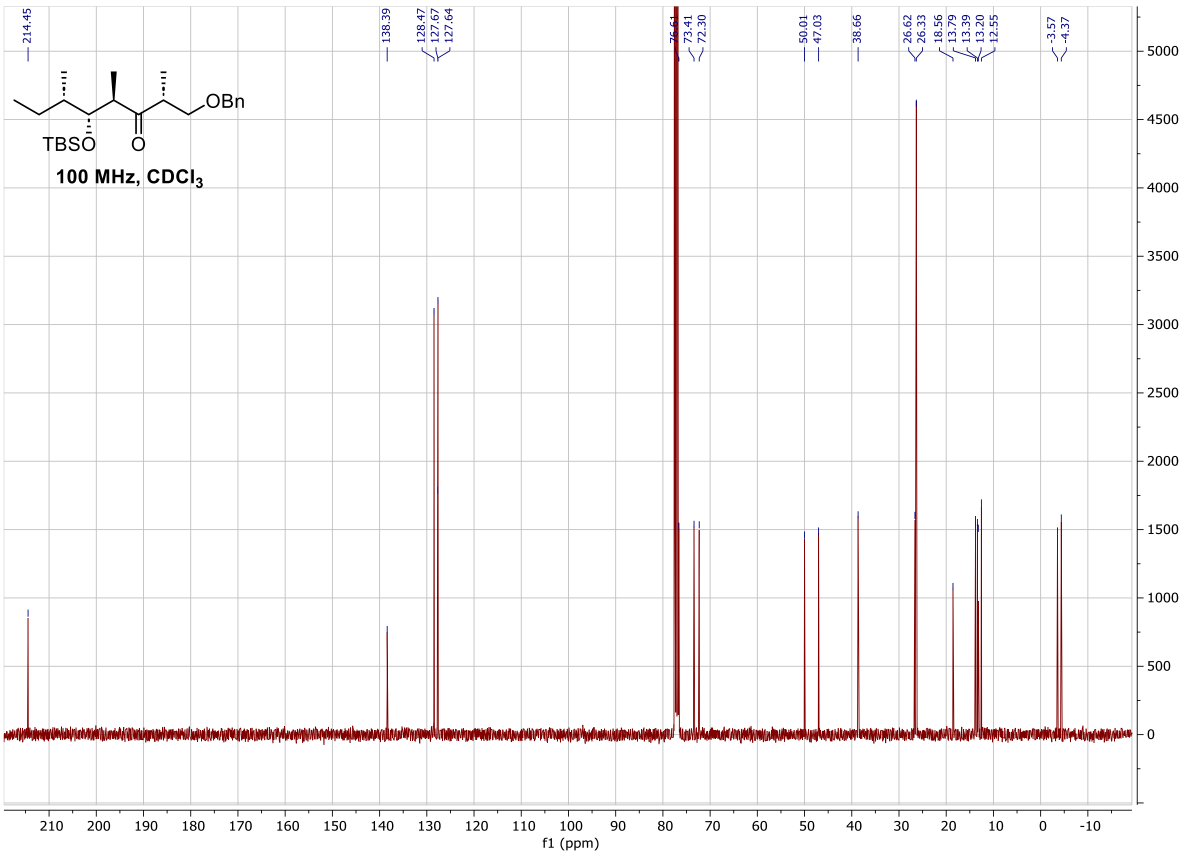

Figure S-13 ^{13}C{^{1}H} NMR spectrum of compound **6**.

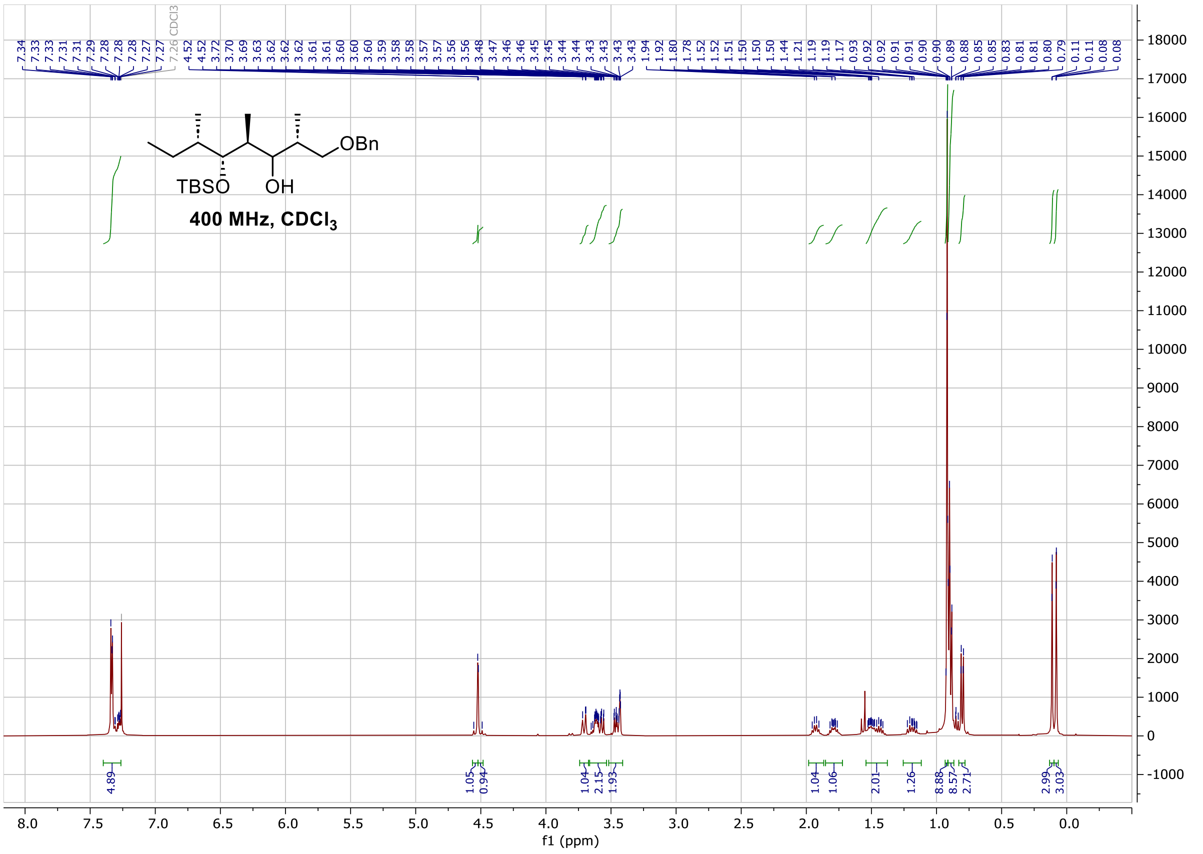

Figure S-14 ^{1}H-NMR spectrum of compound **7**.

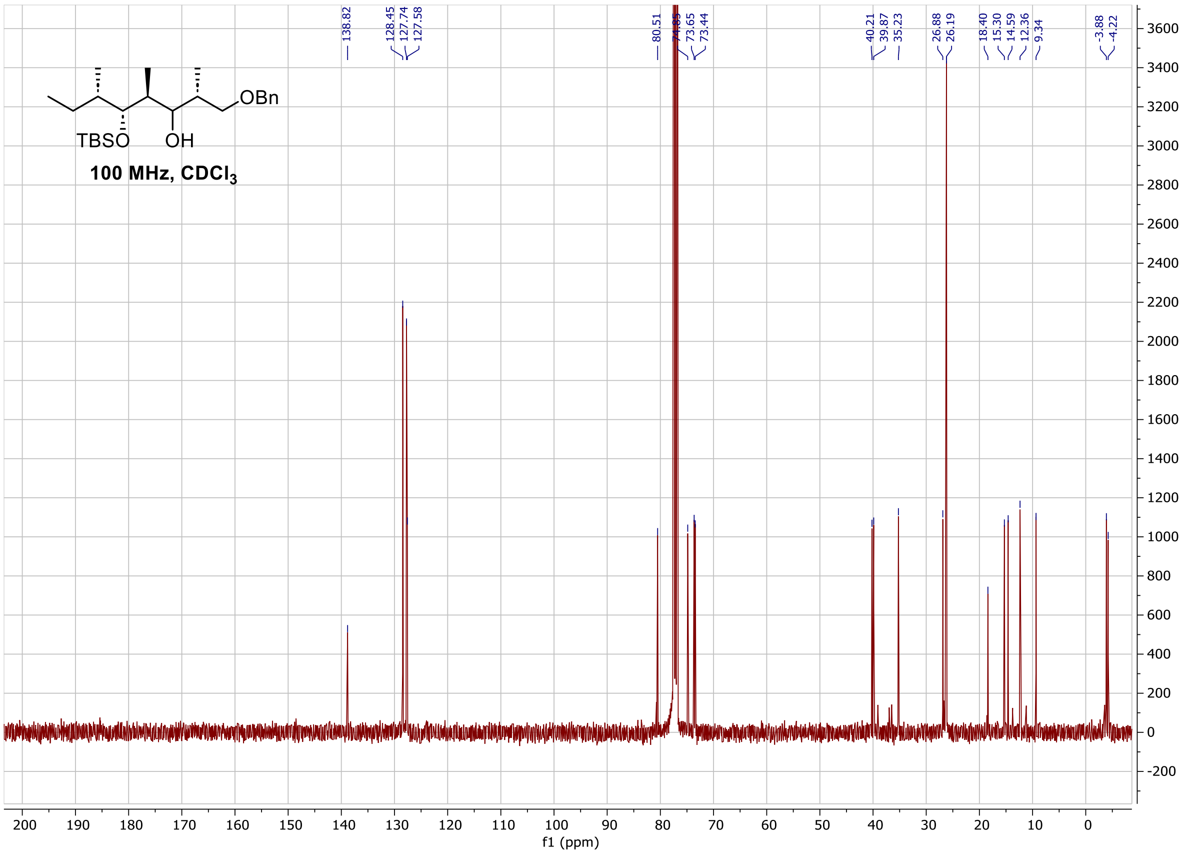

Figure S-15 $^{13}C\{^{1}H\}$ NMR spectrum of compound **7**.

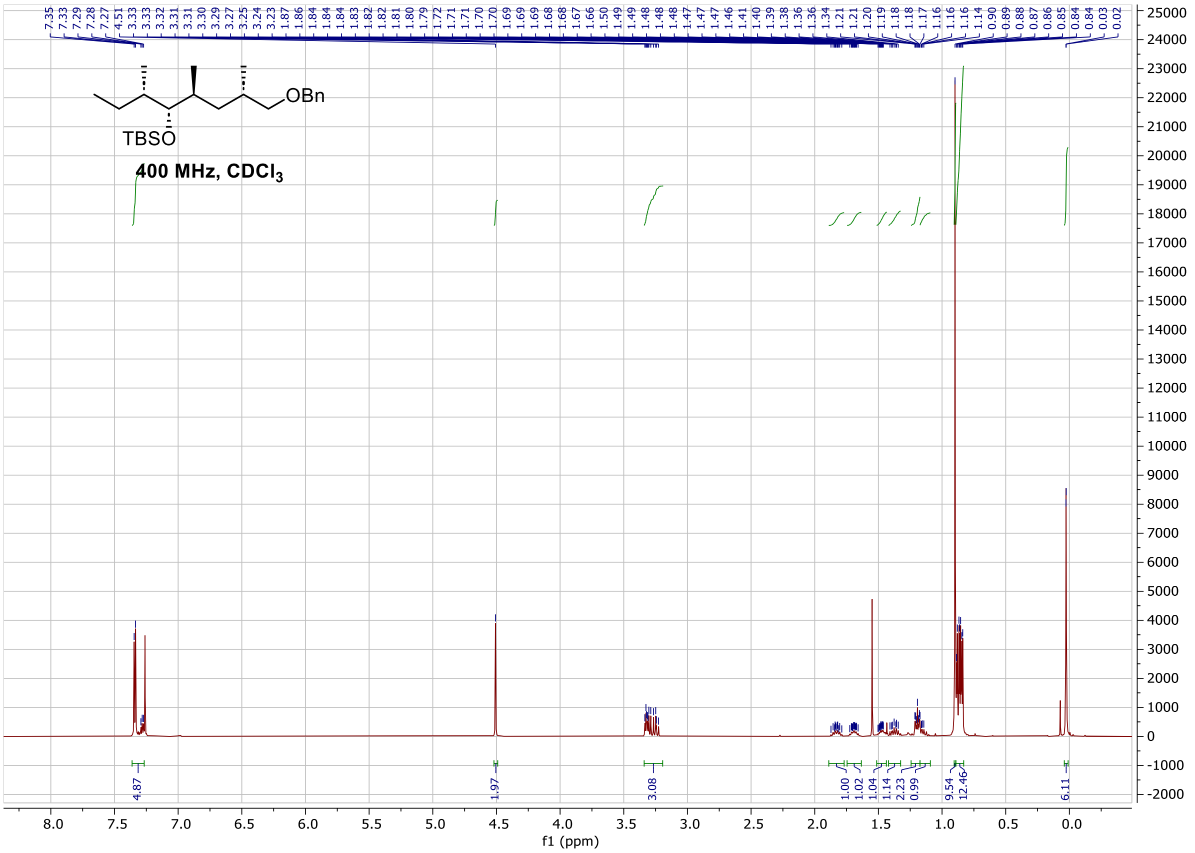

Figure S-16 ^{1}H-NMR spectrum of compound **8**.

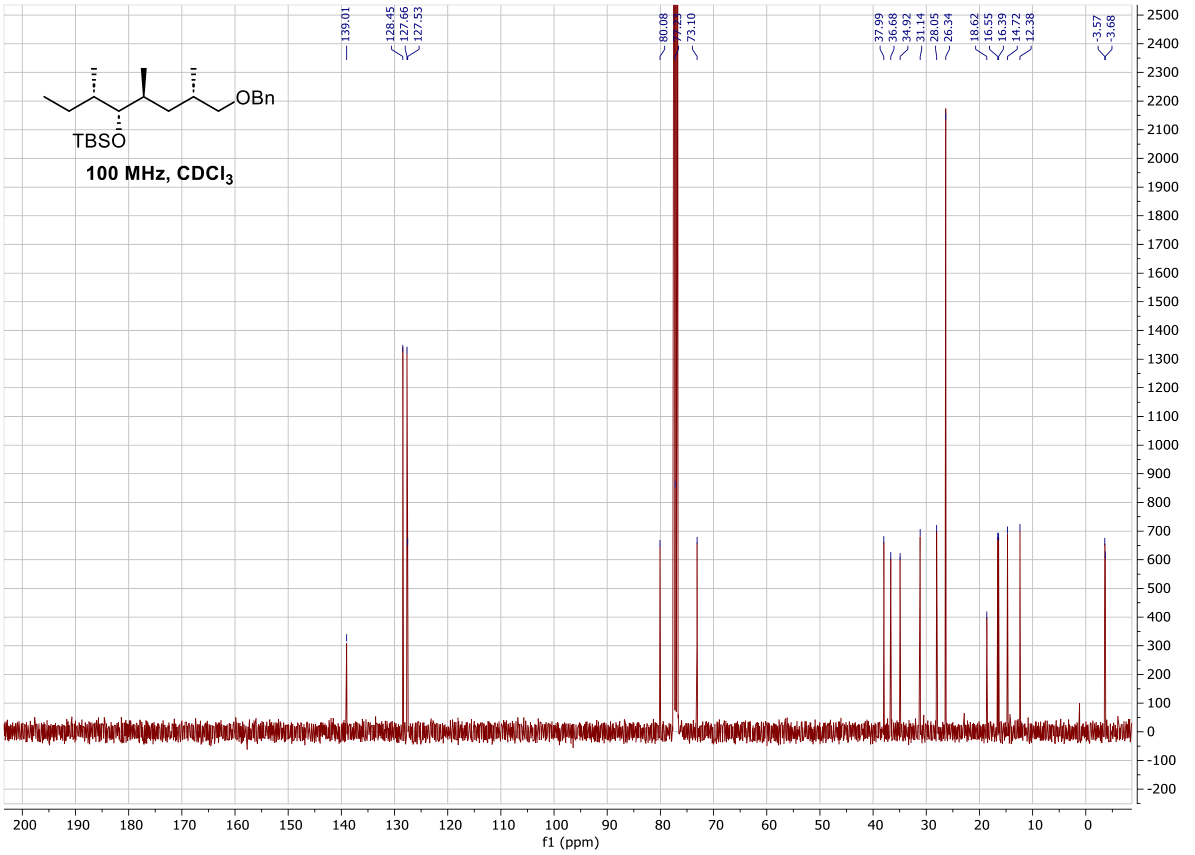

Figure S-17 ^{13}C{^{1}H} NMR spectrum of compound **8**.

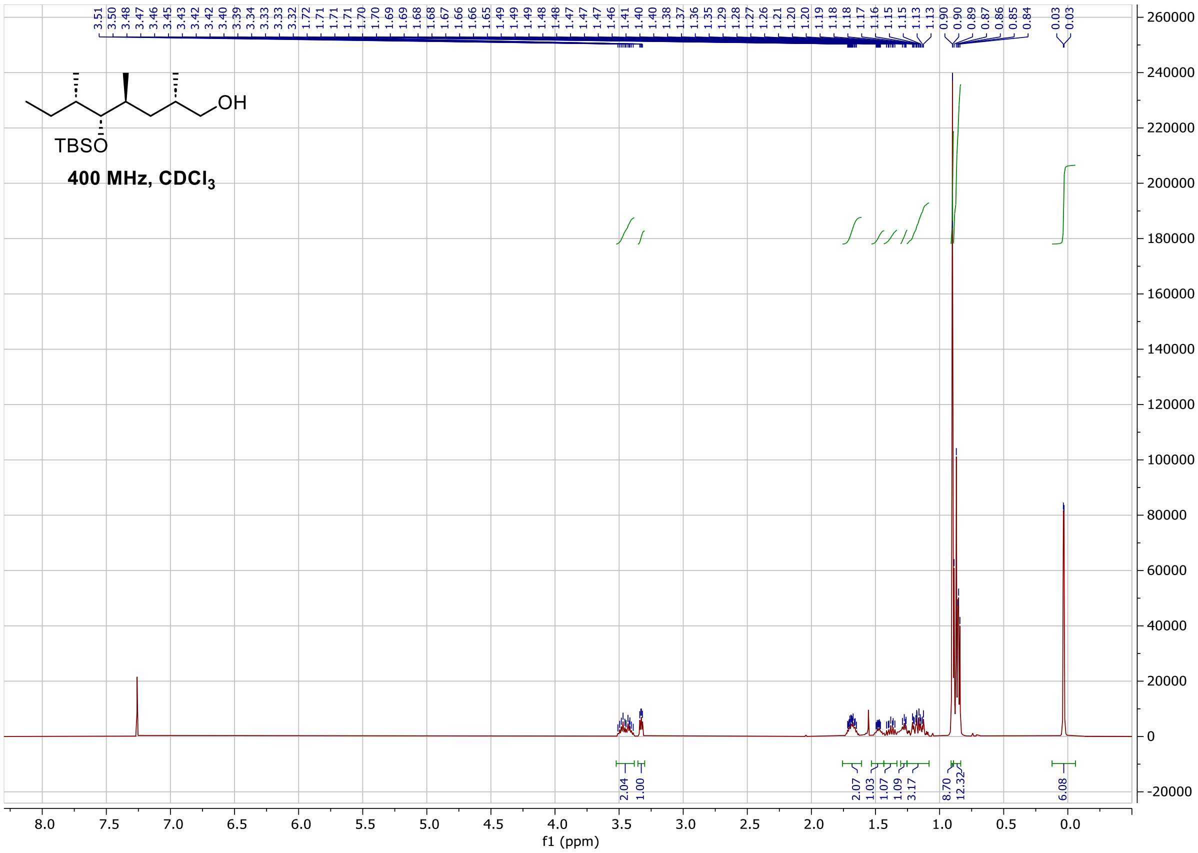

Figure S-18 ^{1}H-NMR spectrum of compound **9**.

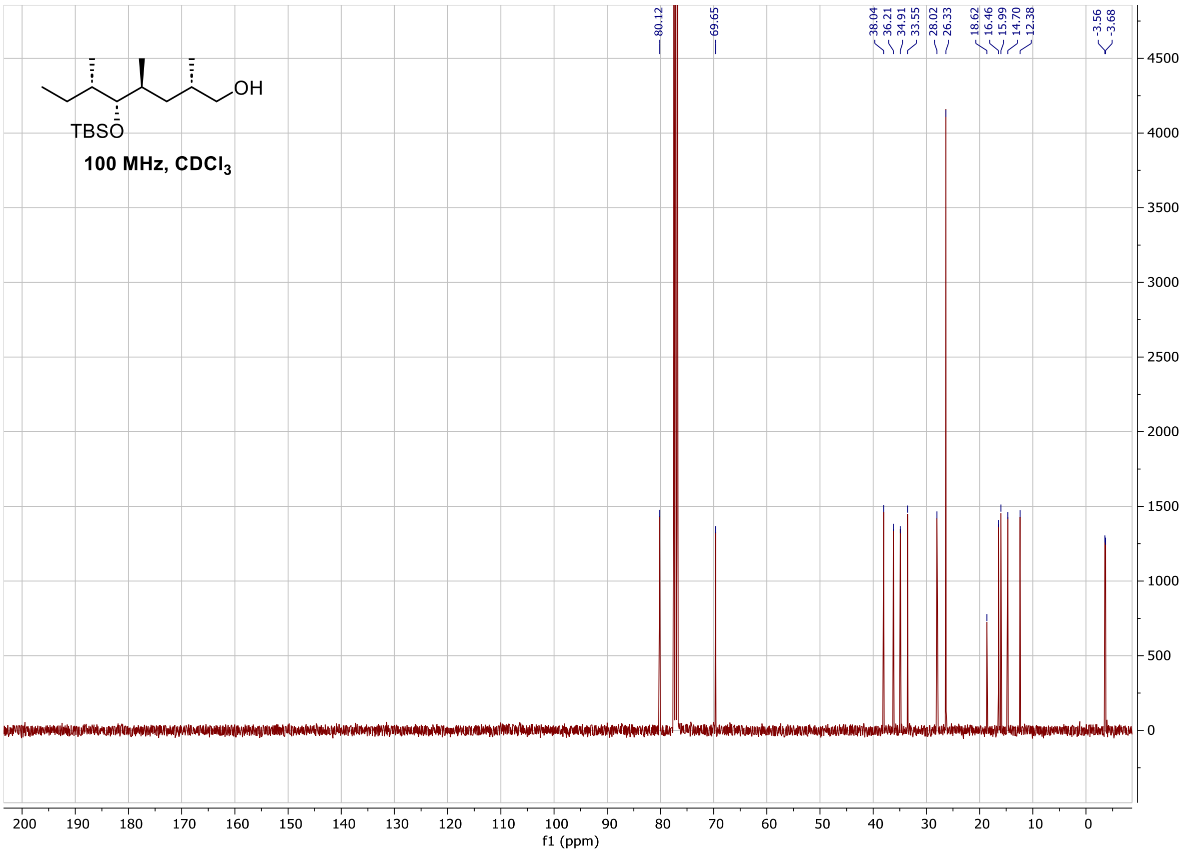

Figure S-19 ^{13}C{^{1}H} NMR spectrum of compound **9**.

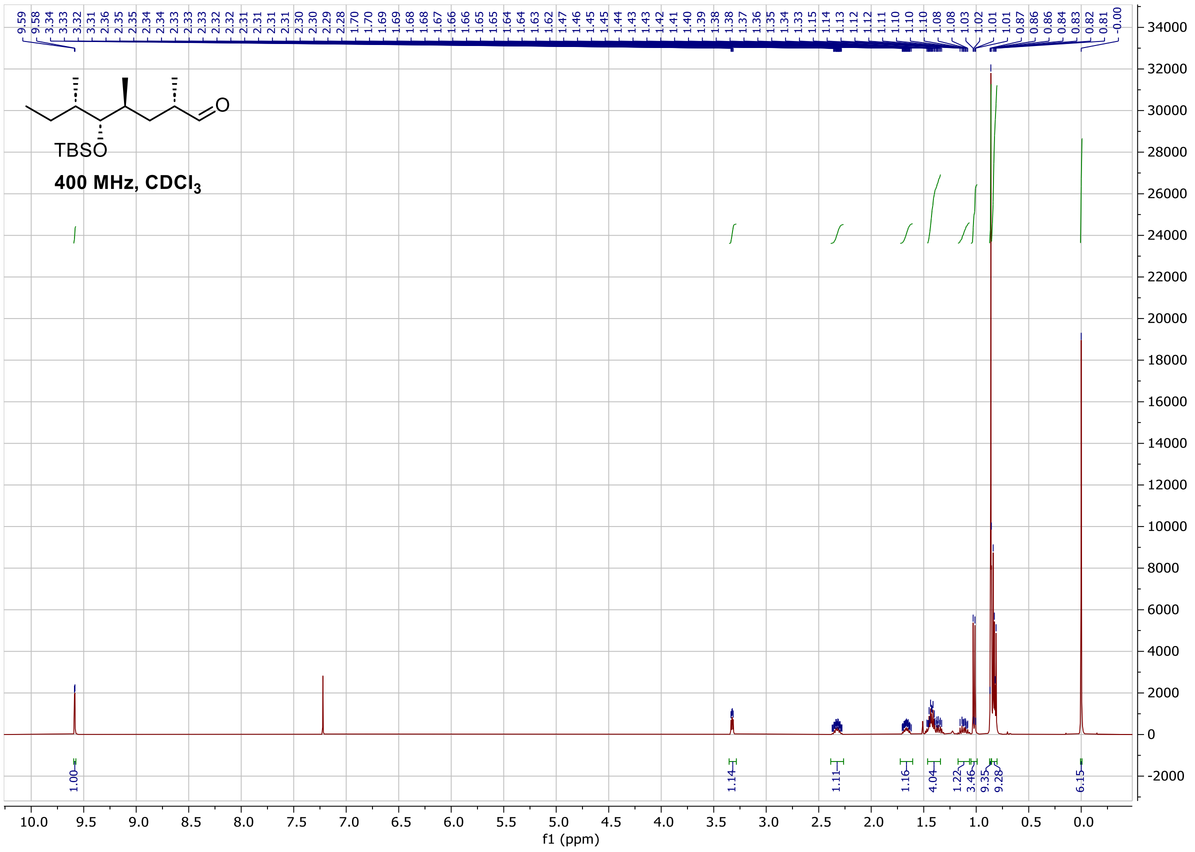

Figure S-20 ^{1}H-NMR spectrum of compound **10**.

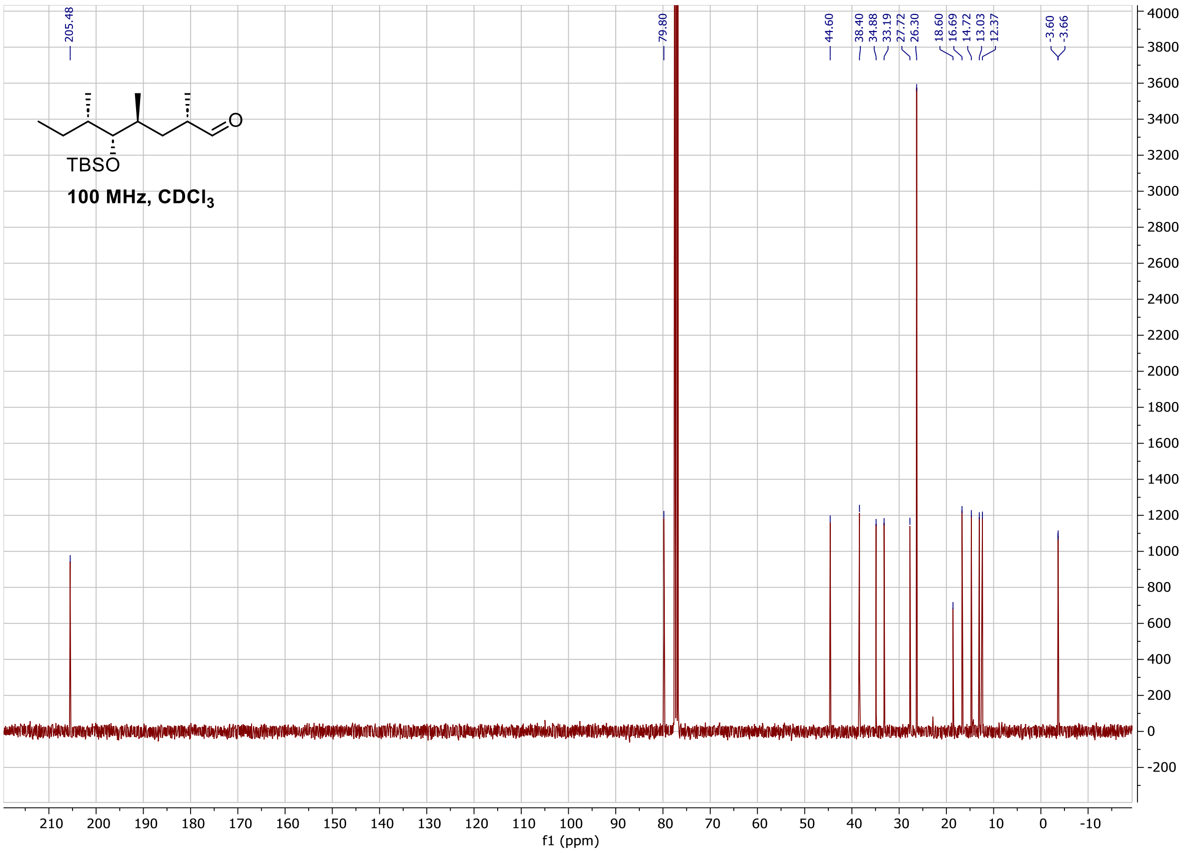

Figure S-21 ^{13}C{^{1}H} NMR spectrum of compound **10**.

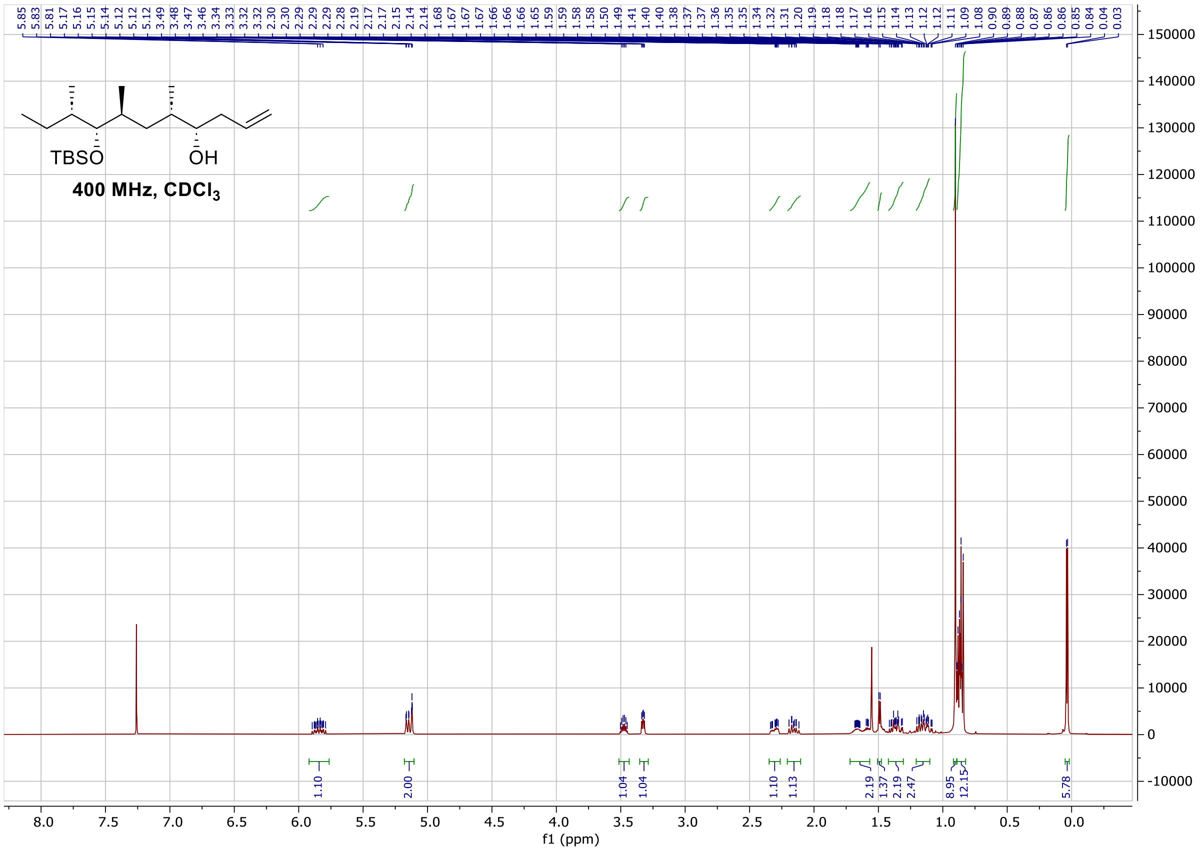

Figure S-22 ^{1}H-NMR spectrum of compound **11**.

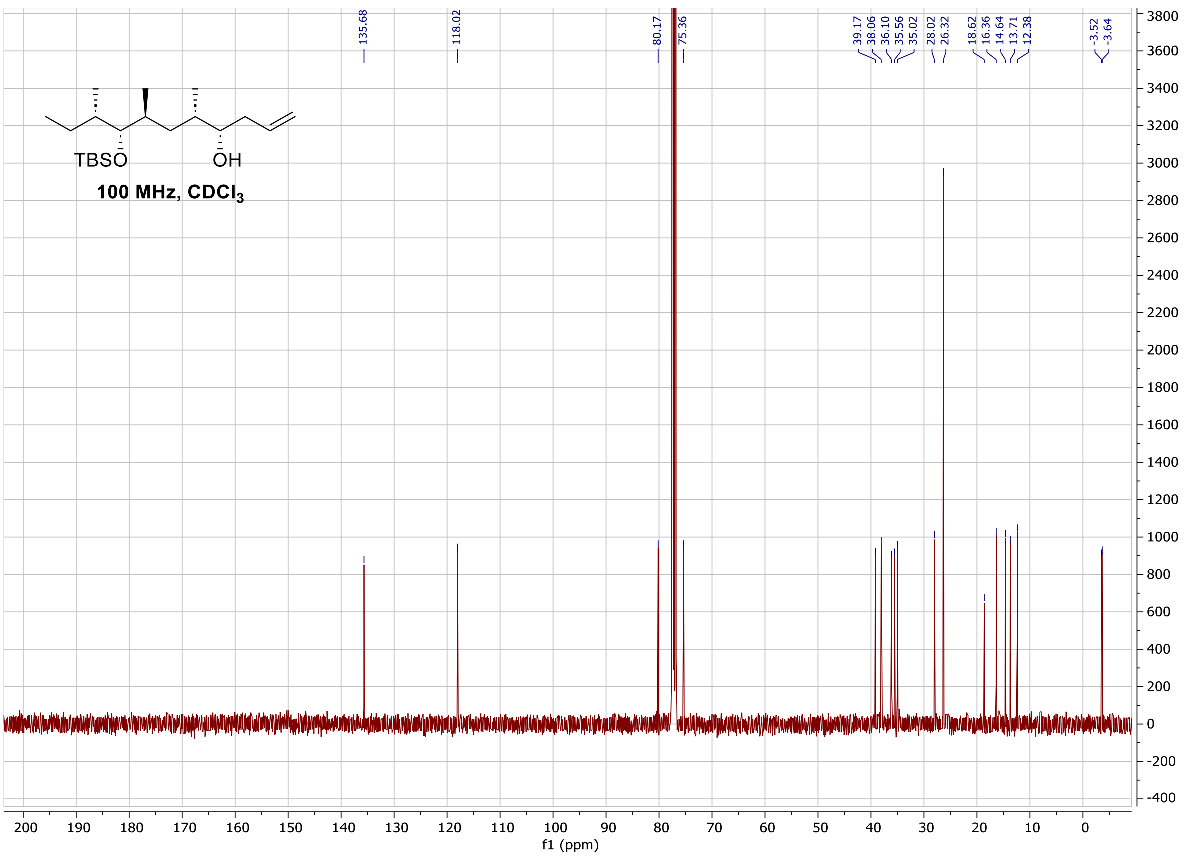

Figure S-23 $^{13}C\{^{1}H\}$ NMR spectrum of compound **11**.

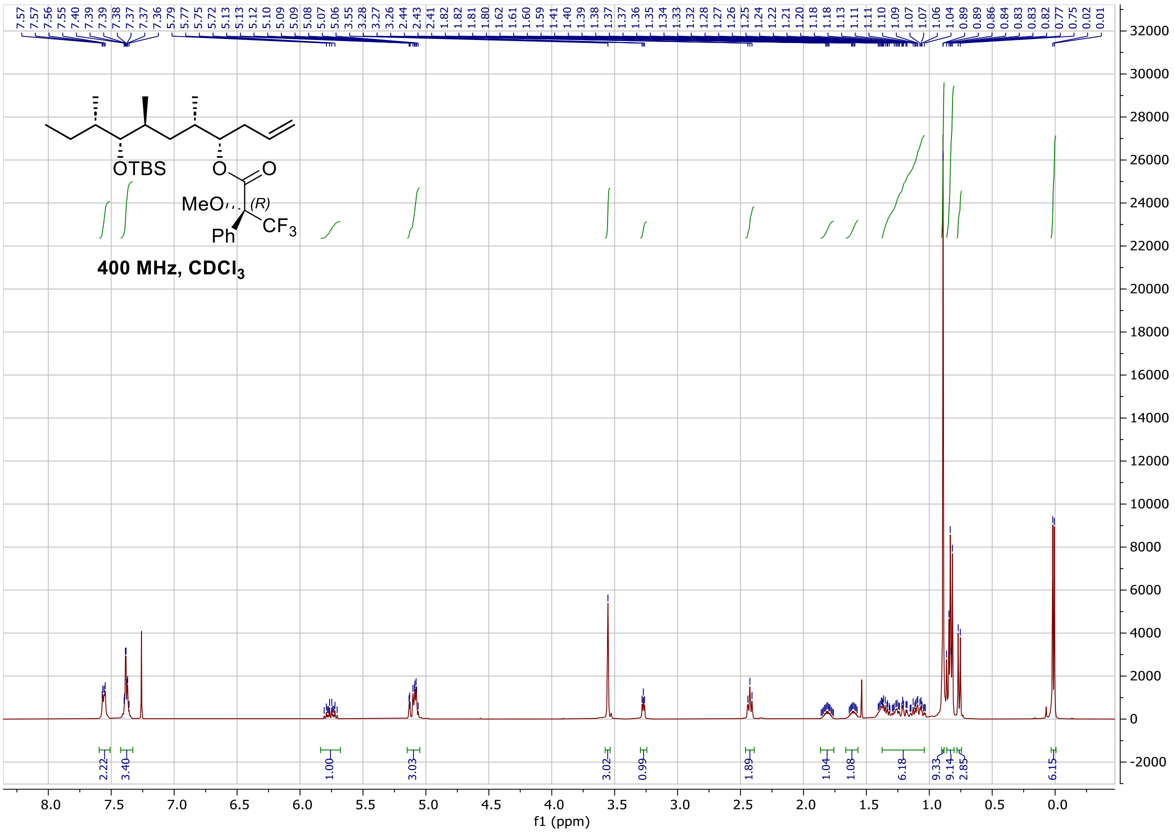

Figure S-24 ^{1}H-NMR spectrum of compound **11-I.**

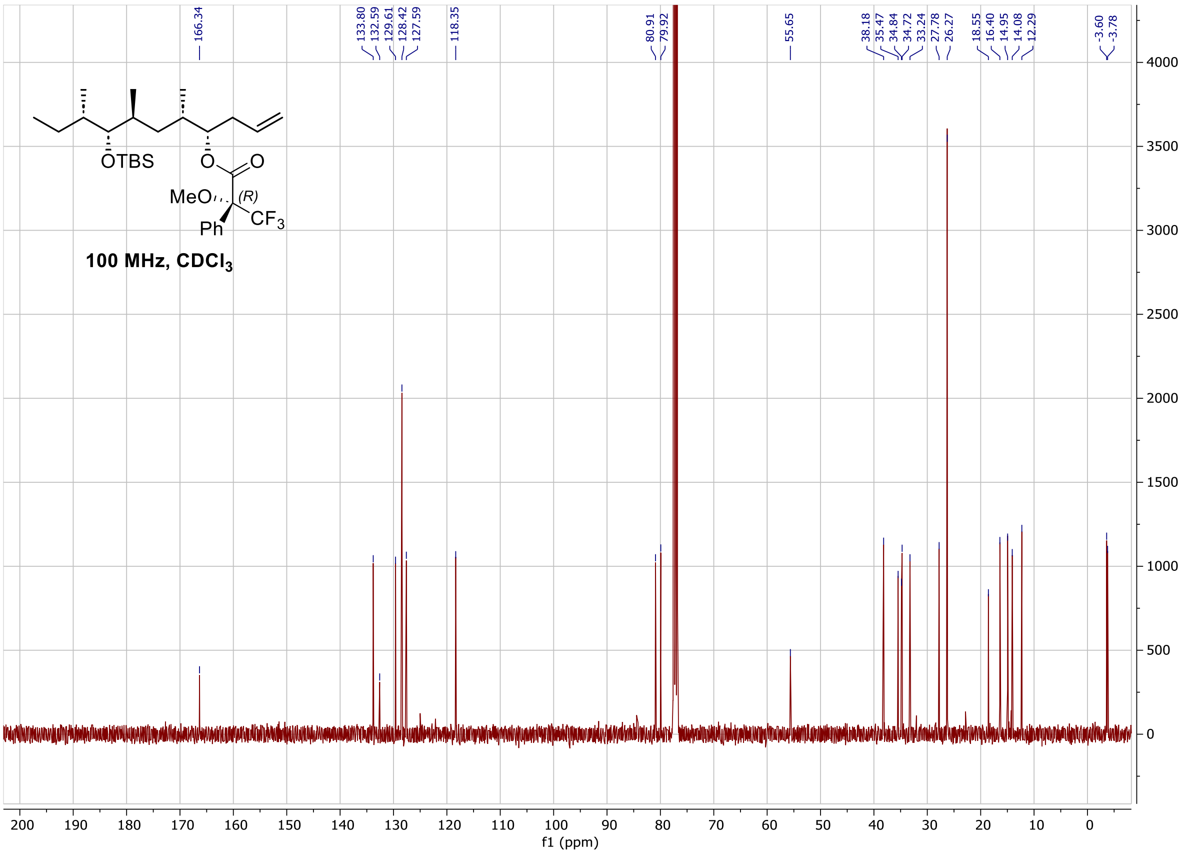

Figure S-25 ^{13}C{^{1}H} NMR spectrum of compound **11-I.**

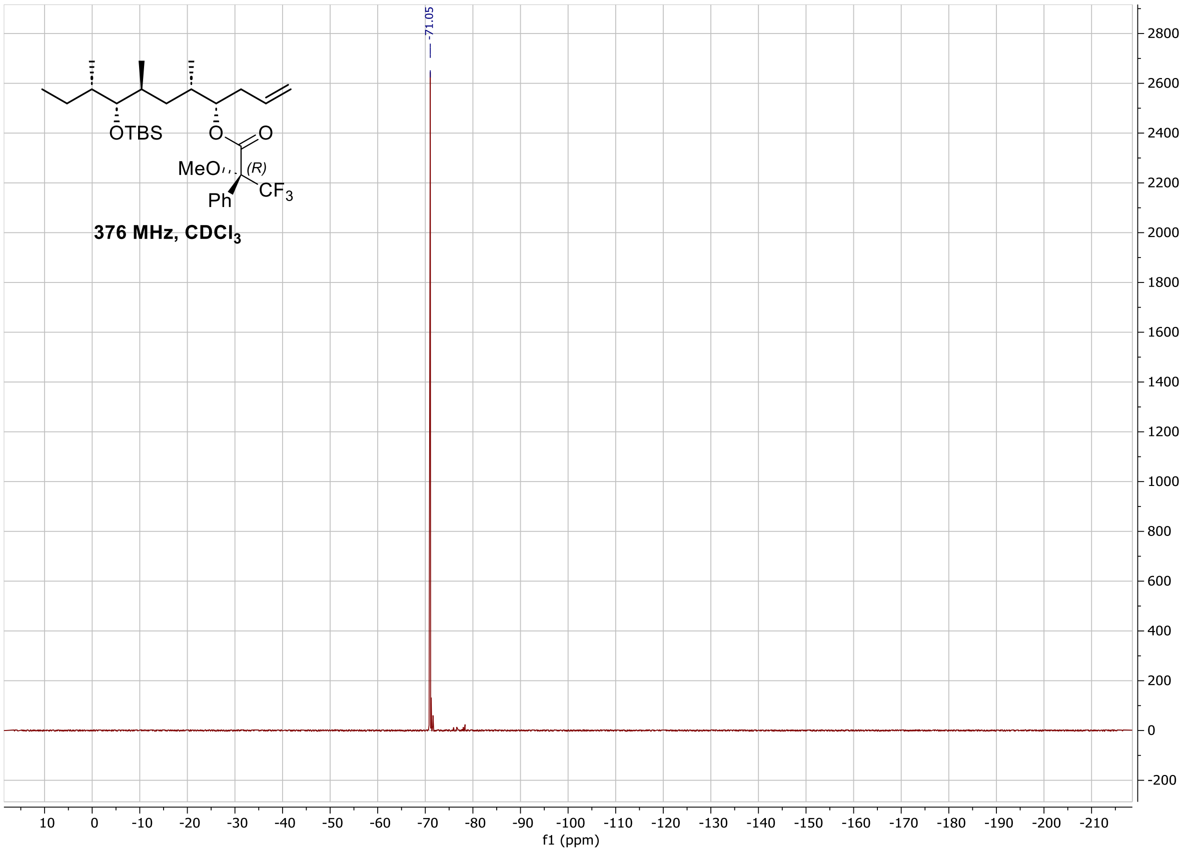

Figure S-26 ^{19}F-NMR spectrum of compound **11-I.**

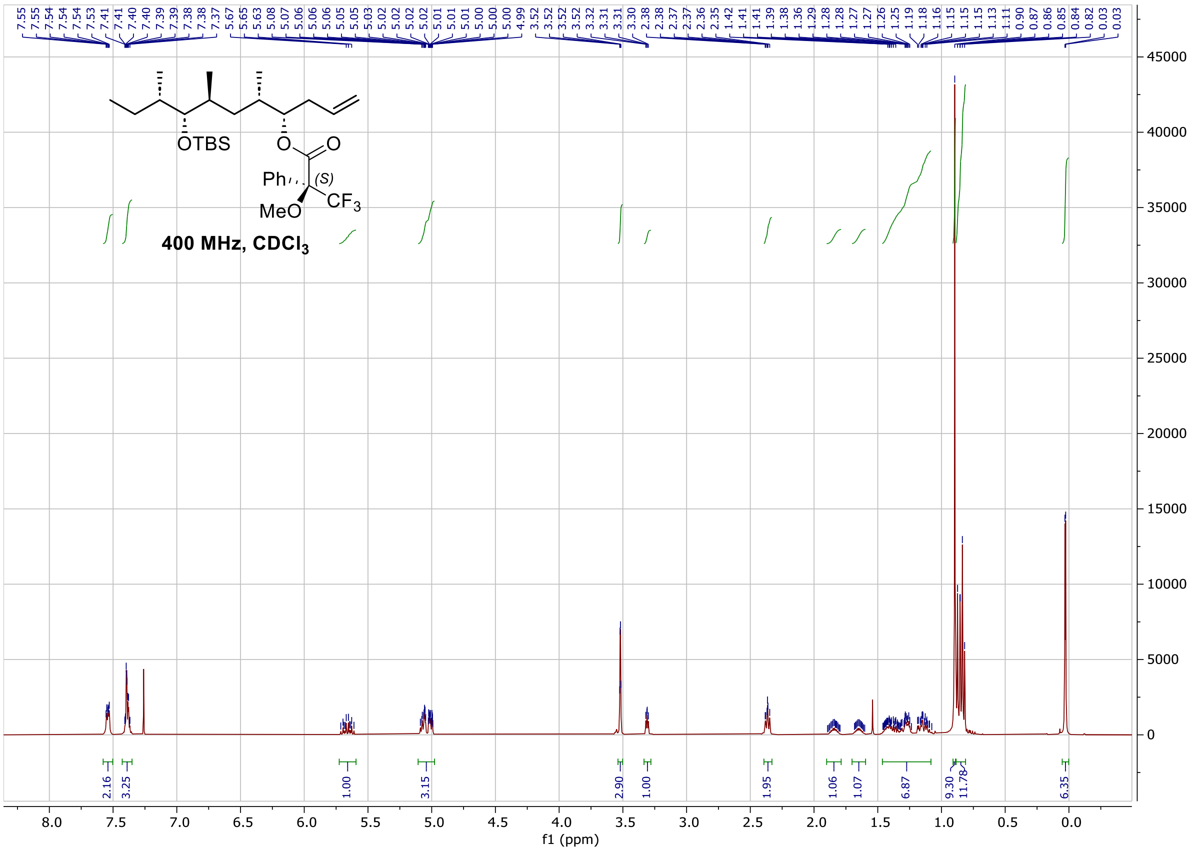

Figure S-27 ^{1}H-NMR spectrum of compound **11-II.**

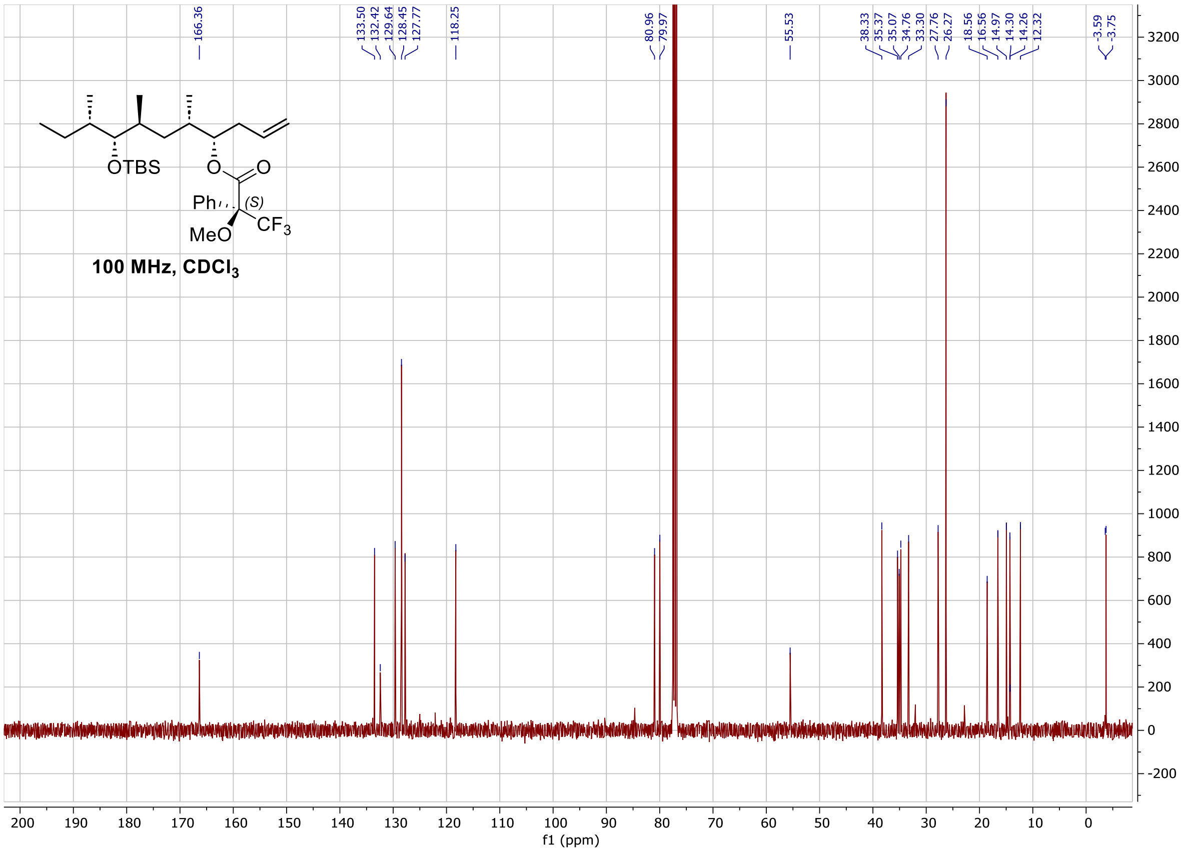

Figure S-28 $^{13}C\{^{1}H\}$ NMR spectrum of compound **11-II.**

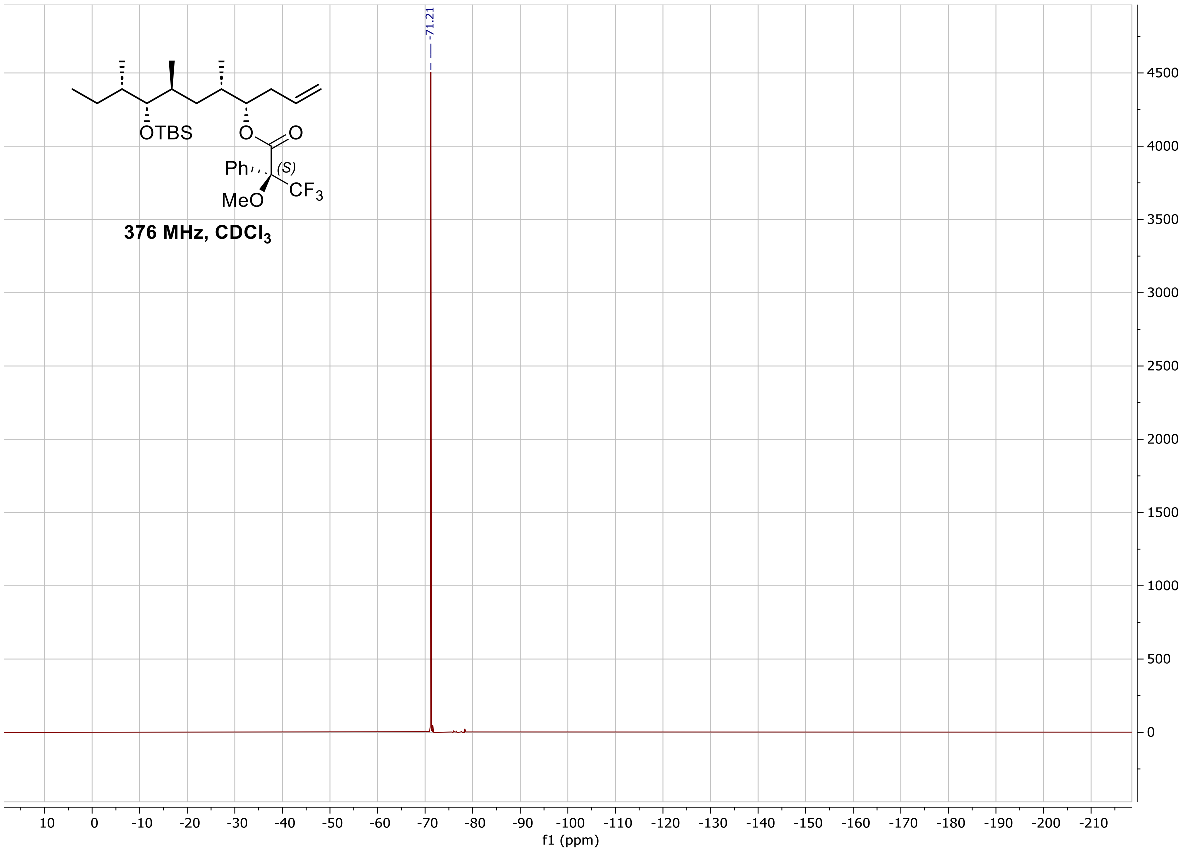

Figure S-29 ^{19}F-NMR spectrum of compound **11-II.**

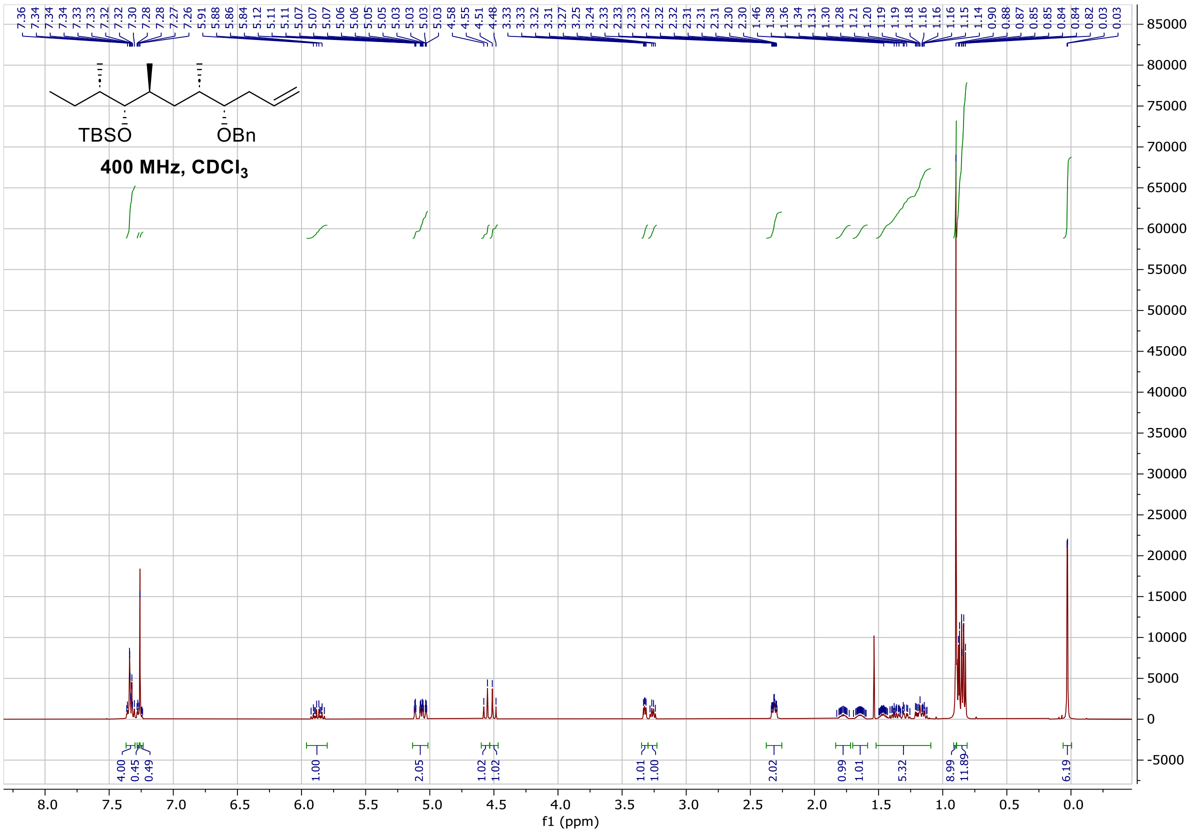

Figure S-30 ^{1}H-NMR spectrum of compound **12**.

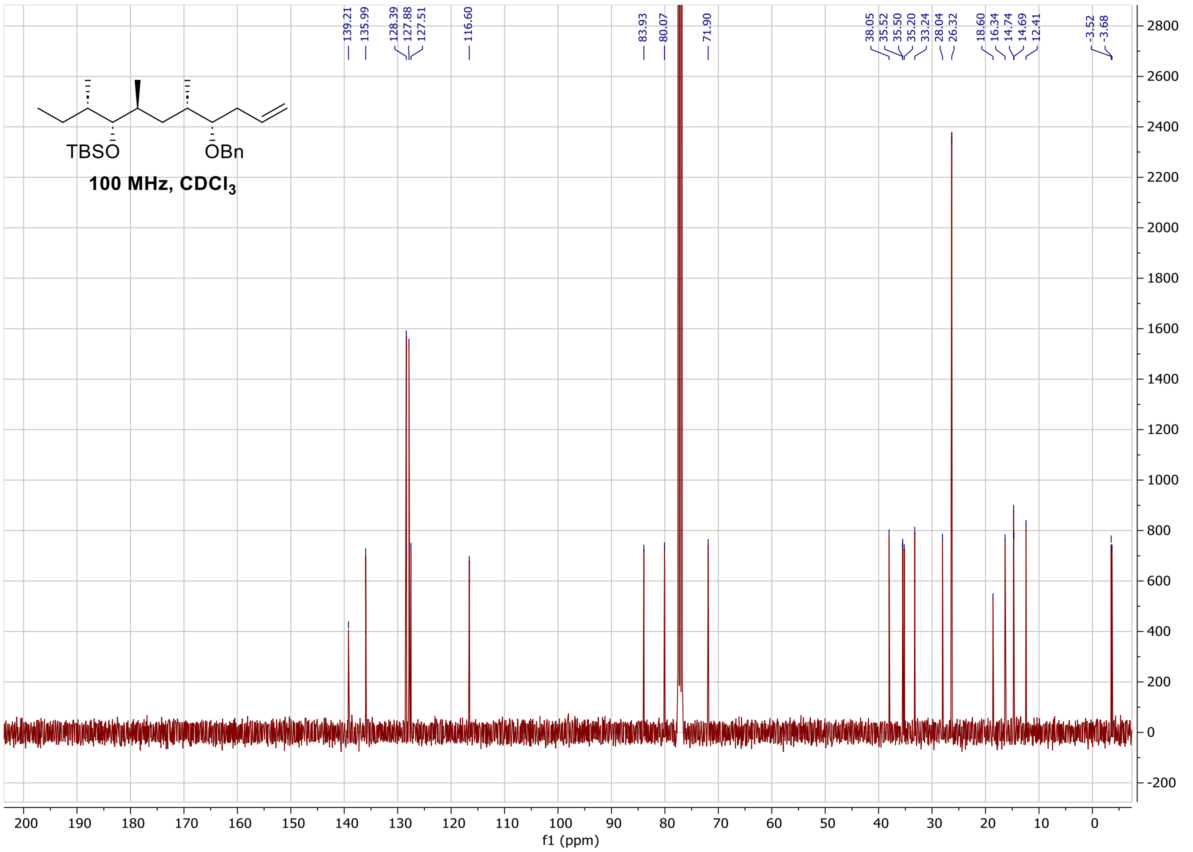

Figure S-31 ^{13}C{^{1}H} NMR spectrum of compound **12**.

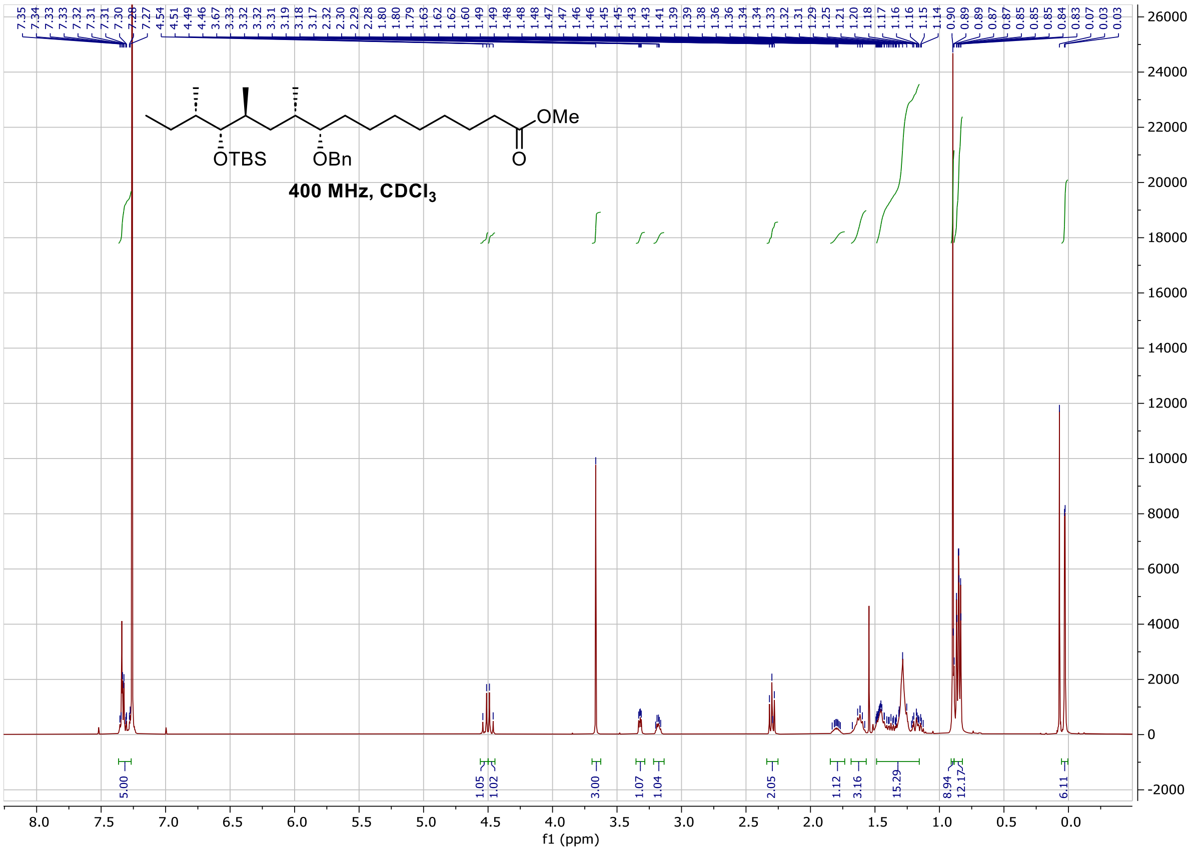

Figure S-32 ^{1}H-NMR spectrum of compound **14**.

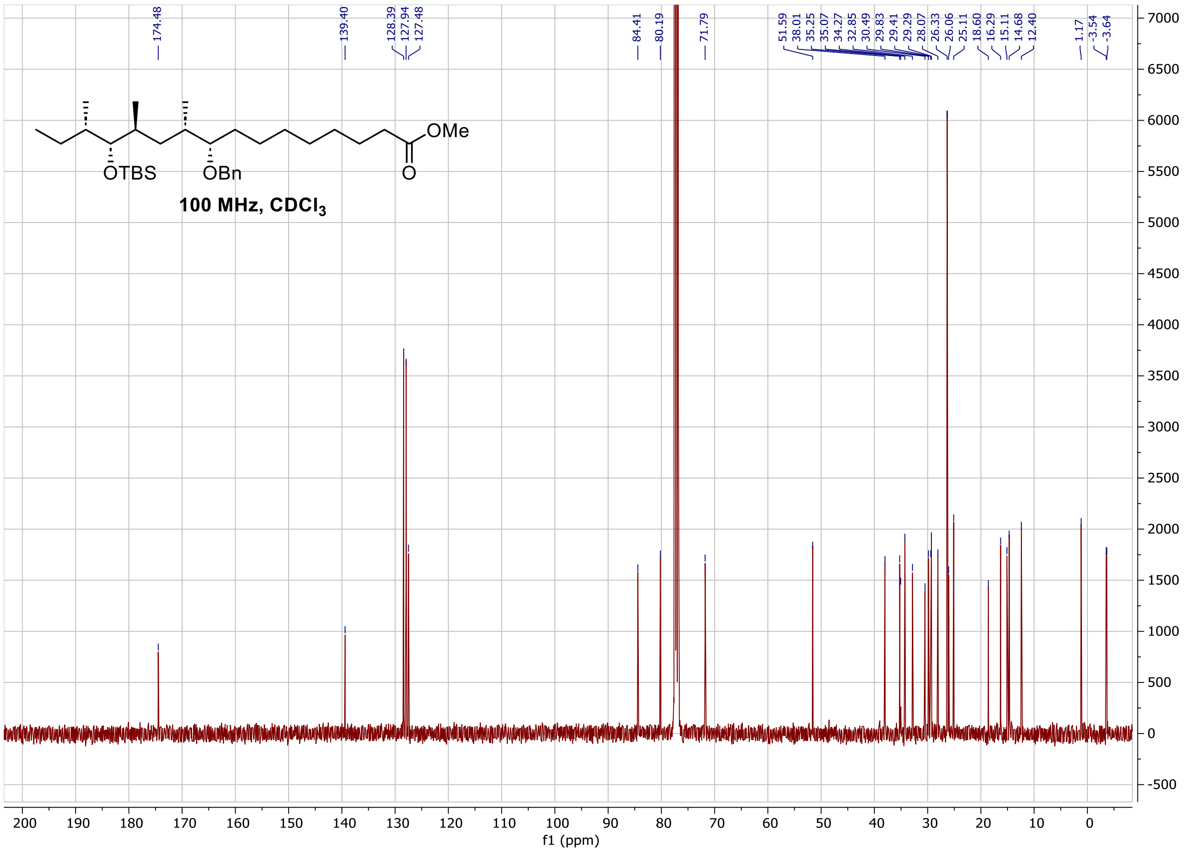

Figure S-33 $^{13}C\{^{1}H\}$ NMR spectrum of compound **14**.

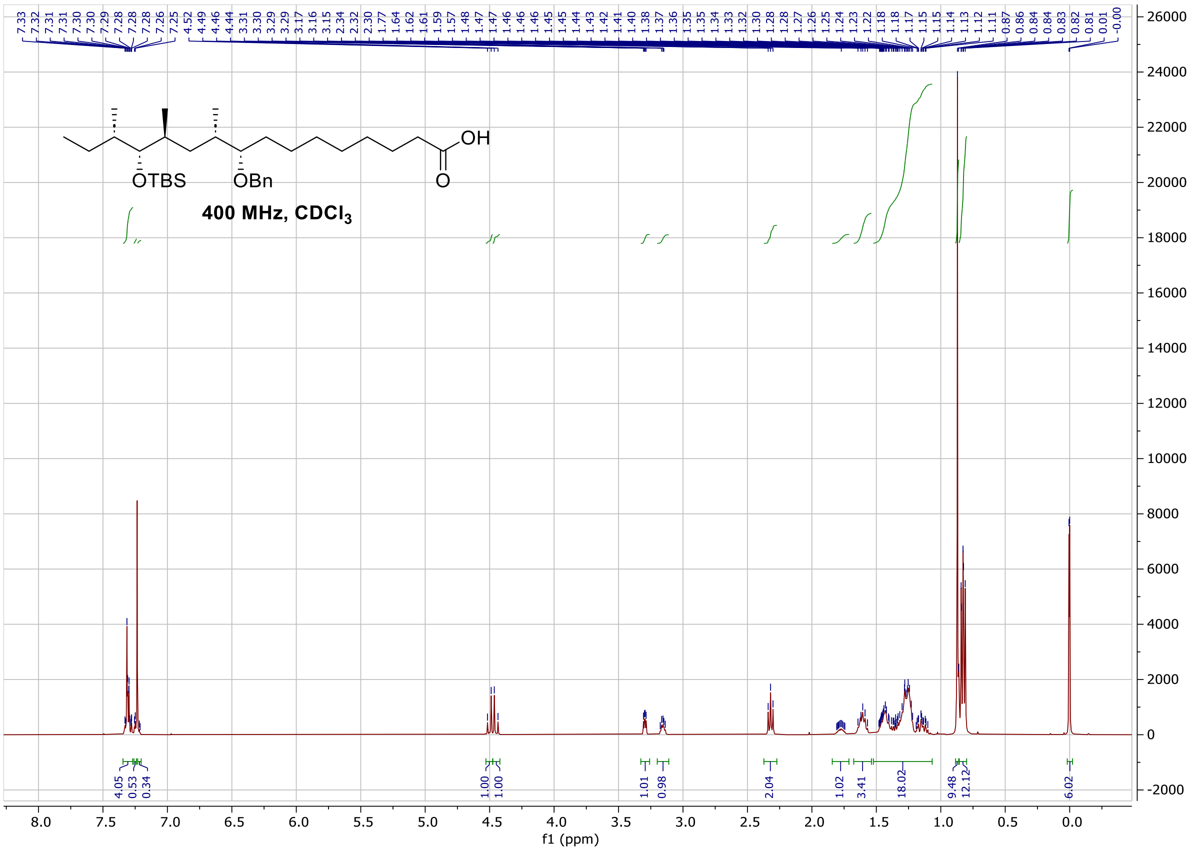

Figure S-34 ^{1}H-NMR spectrum of compound **15**.

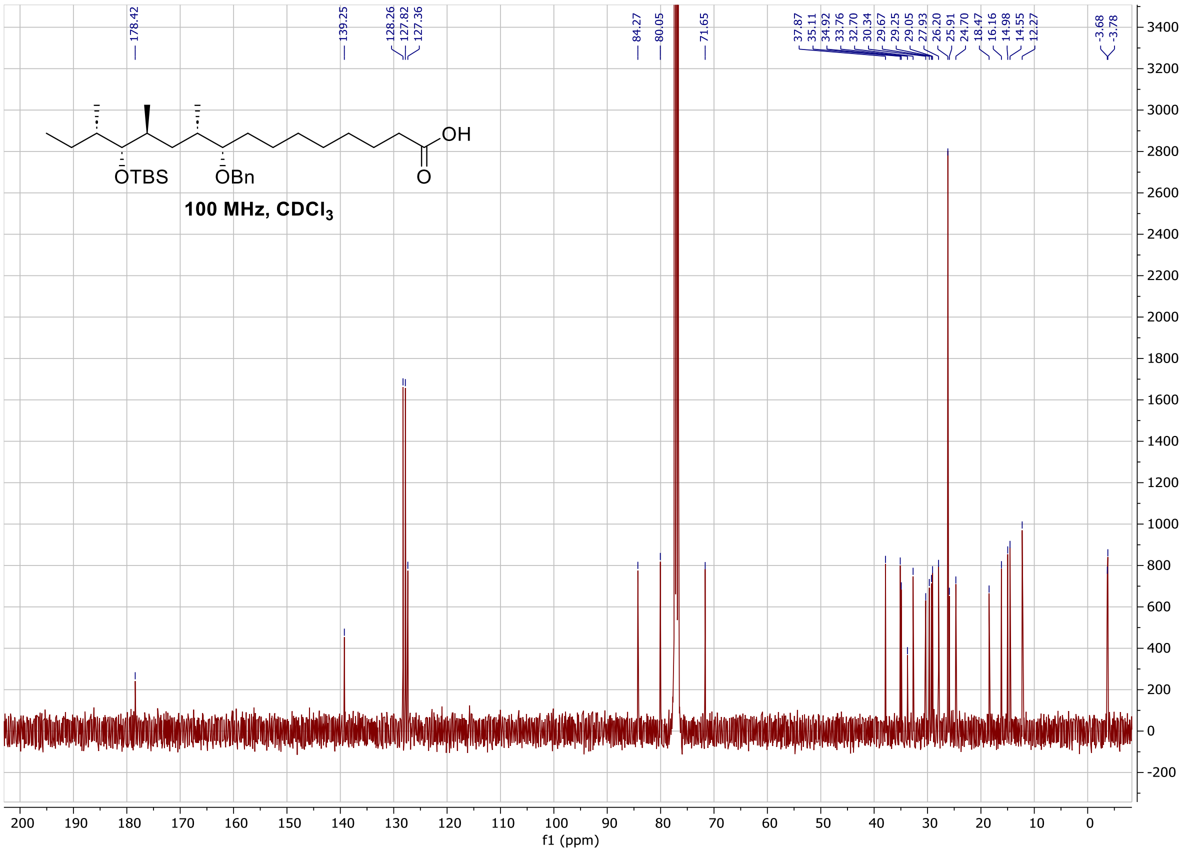

Figure S-35 $^{13}C\{^{1}H\}$ NMR spectrum of compound **15**.

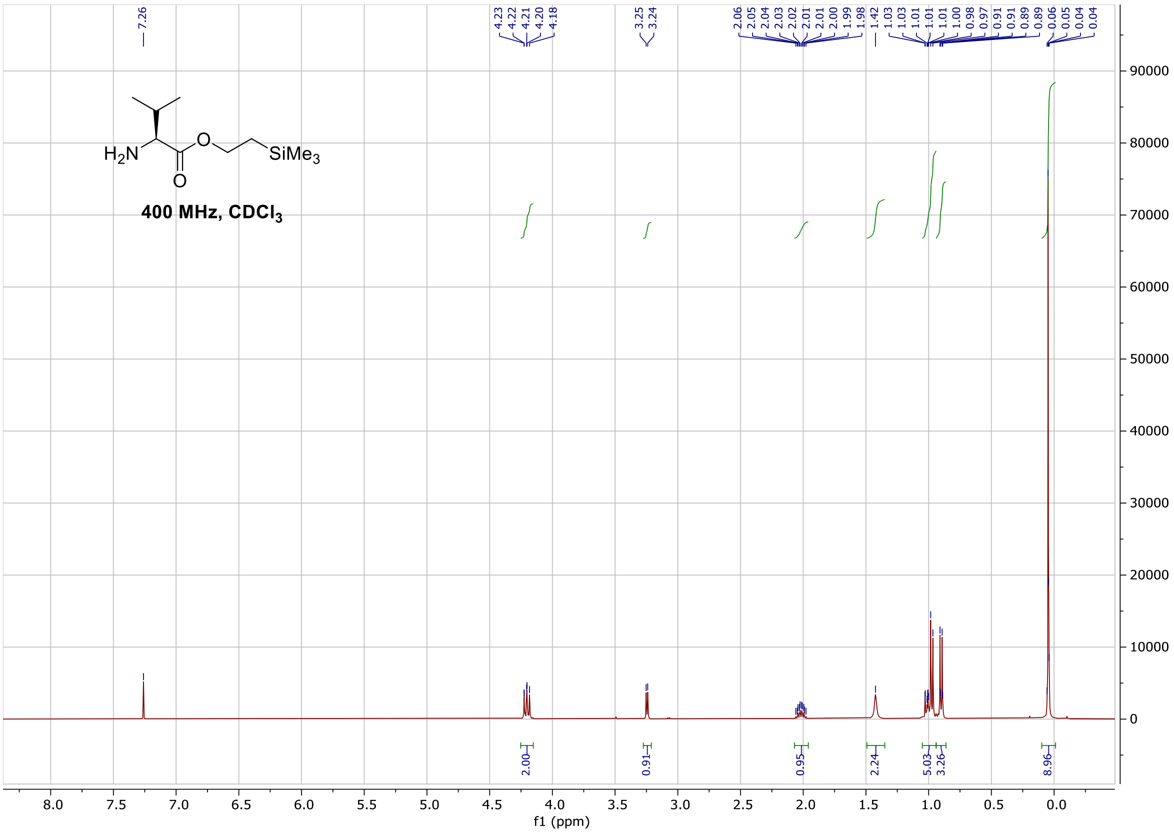

Figure S-36 ^{1}H-NMR spectrum of compound **16**.[2]

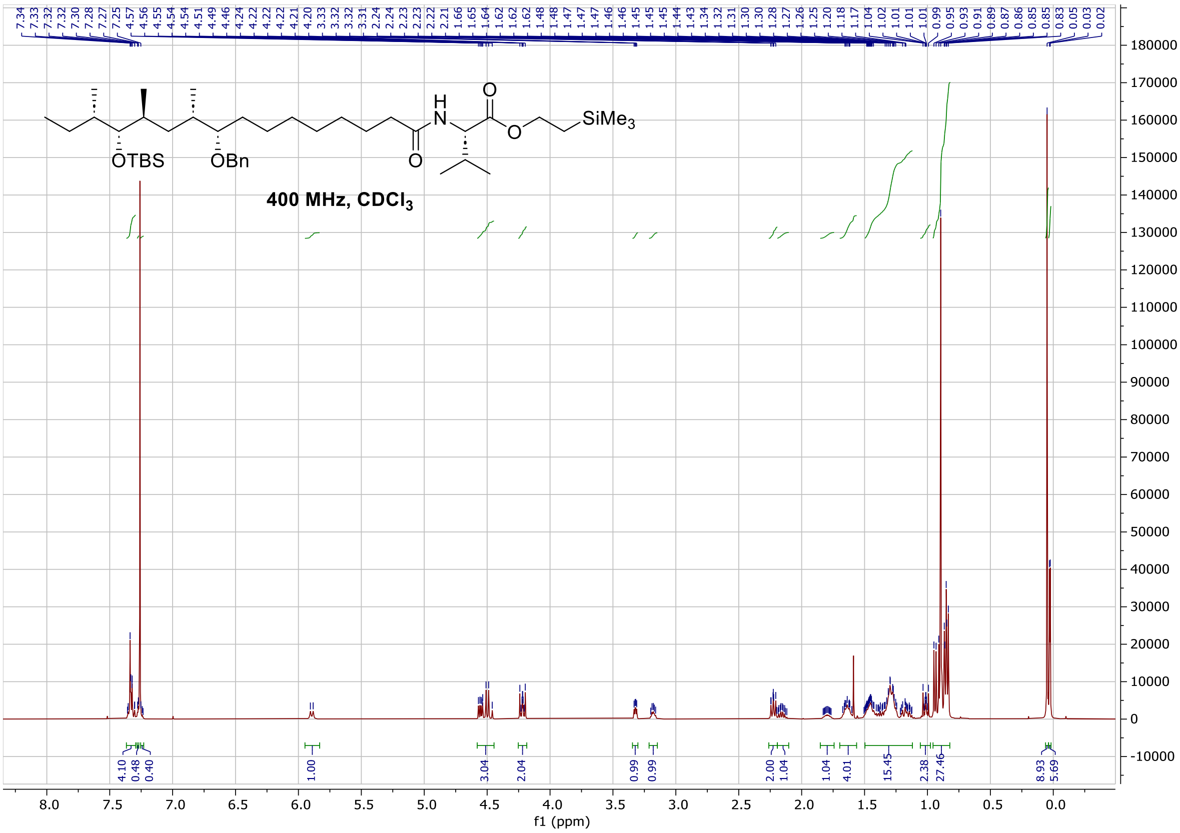

Figure S-37 ^{1}H-NMR spectrum of compound **17**.

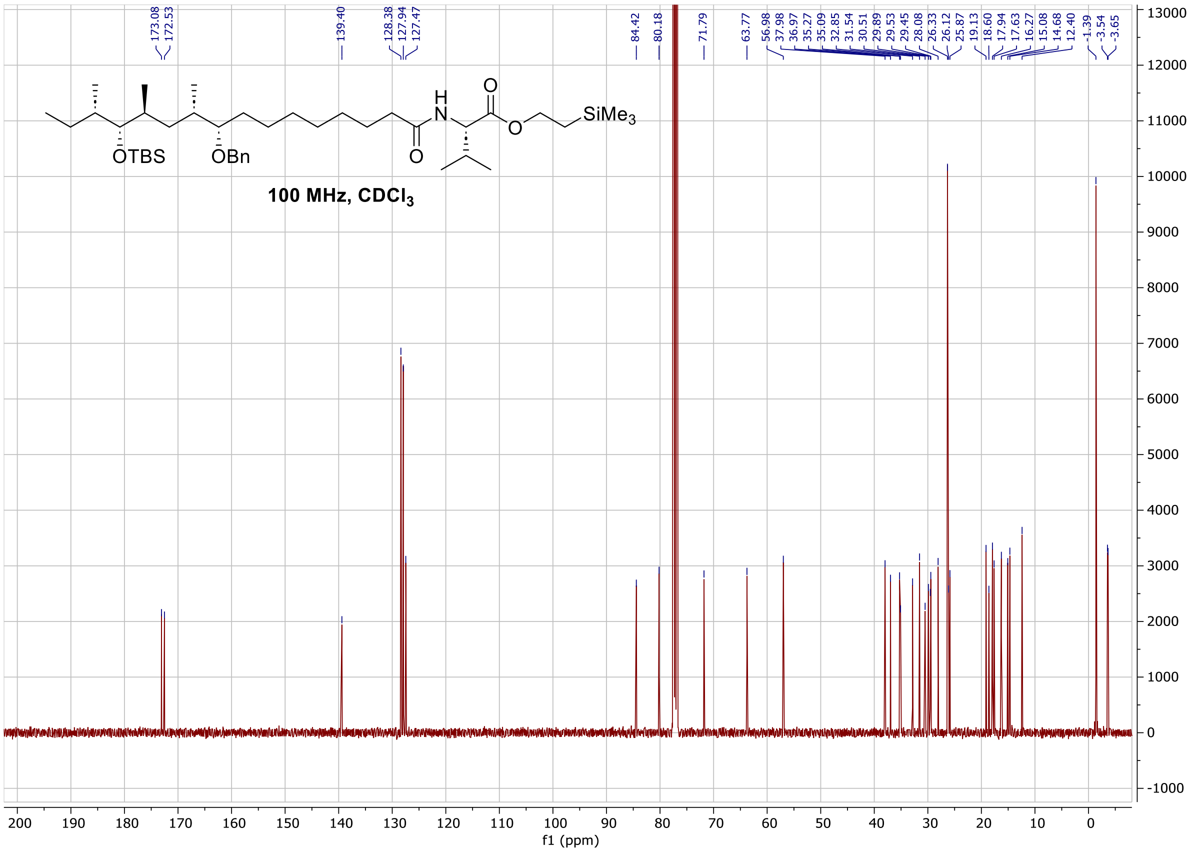

Figure S-38 $^{13}C\{^{1}H\}$ NMR spectrum of compound **17**.

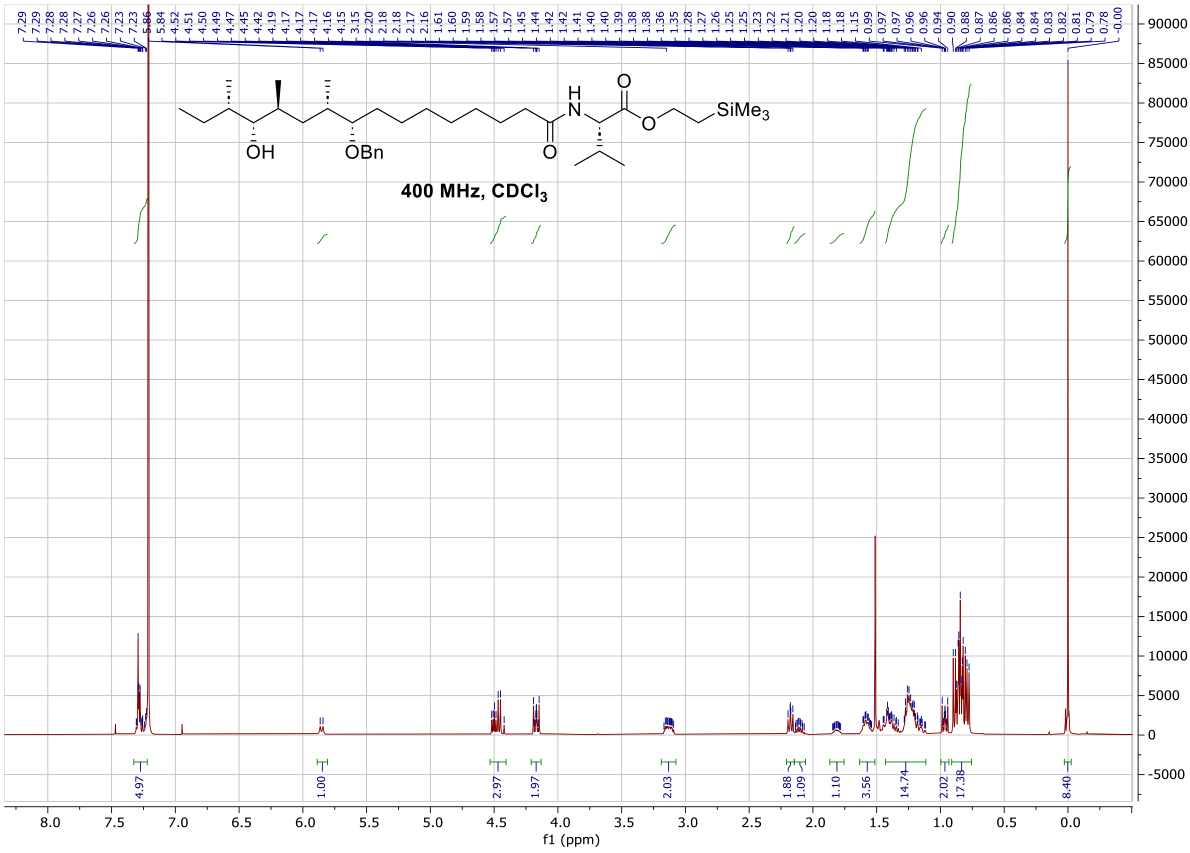

Figure S-39 ^{1}H-NMR spectrum of compound **18**.

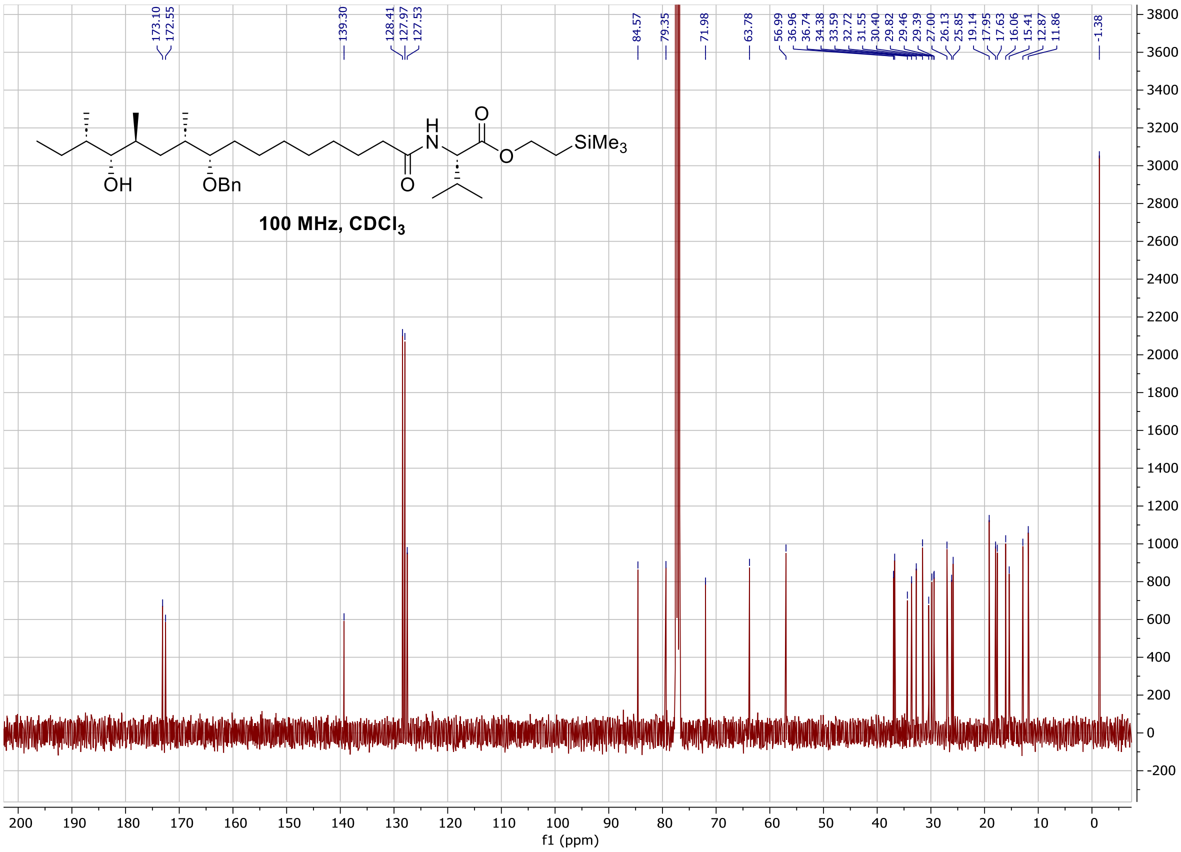

Figure S-40 $^{13}C\{^{1}H\}$ NMR spectrum of compound **18**.

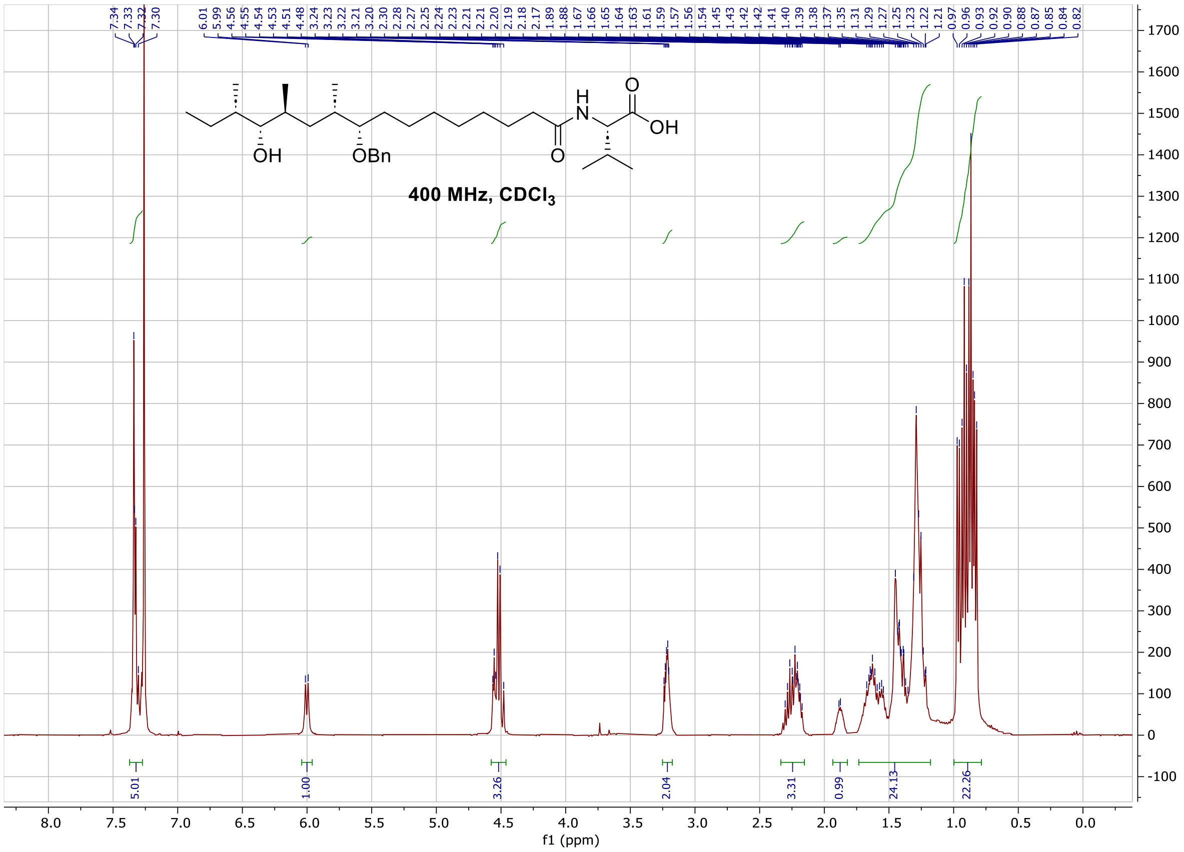

Figure S-41 ^{1}H-NMR spectrum of compound **19**.

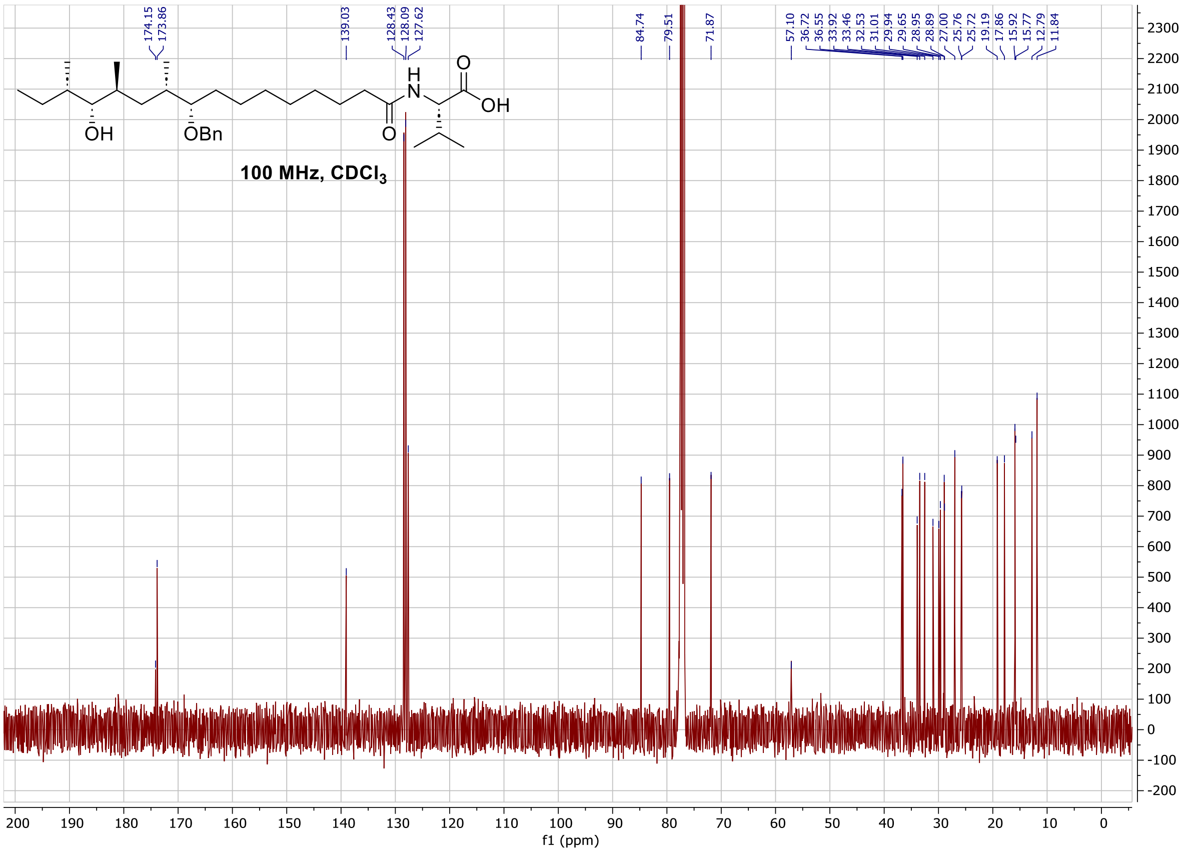

Figure S-42 $^{13}C\{^{1}H\}$ NMR spectrum of compound **19**.

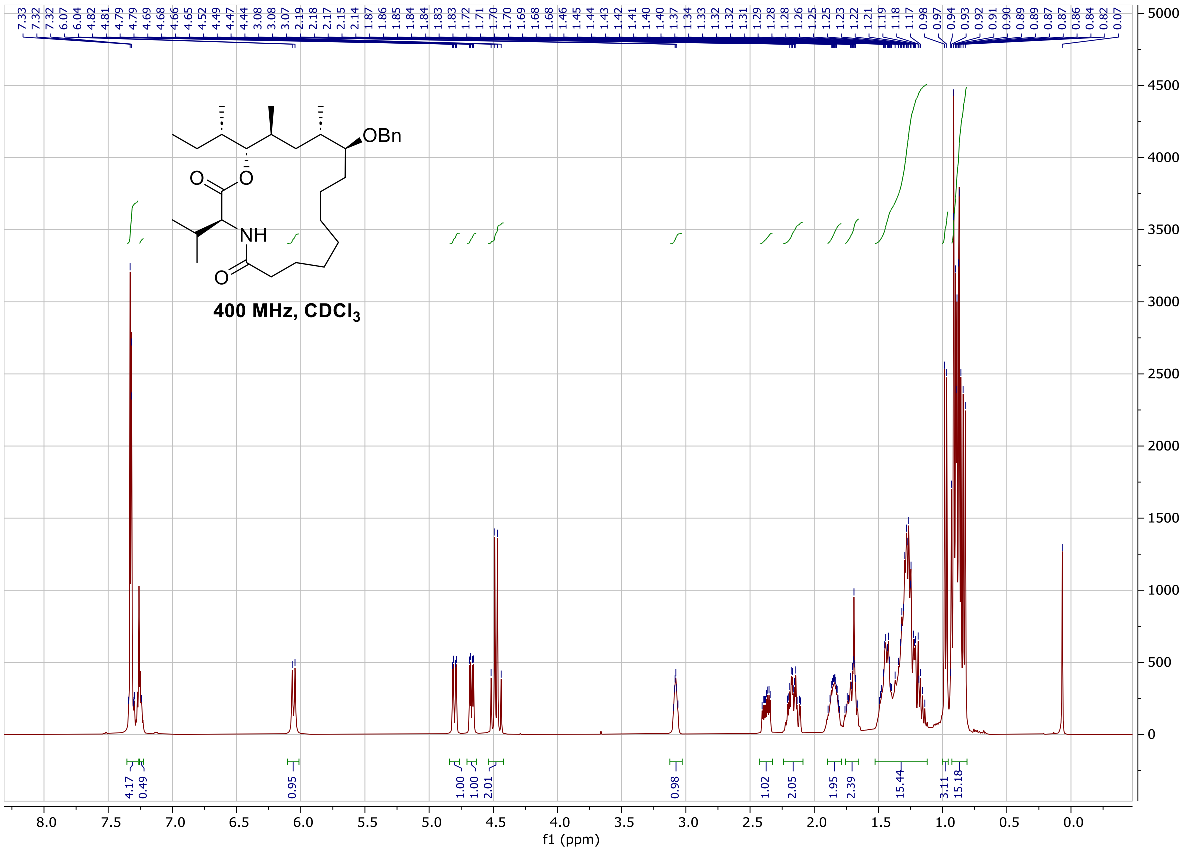

Figure S-43 ^{1}H-NMR spectrum of compound **20**.

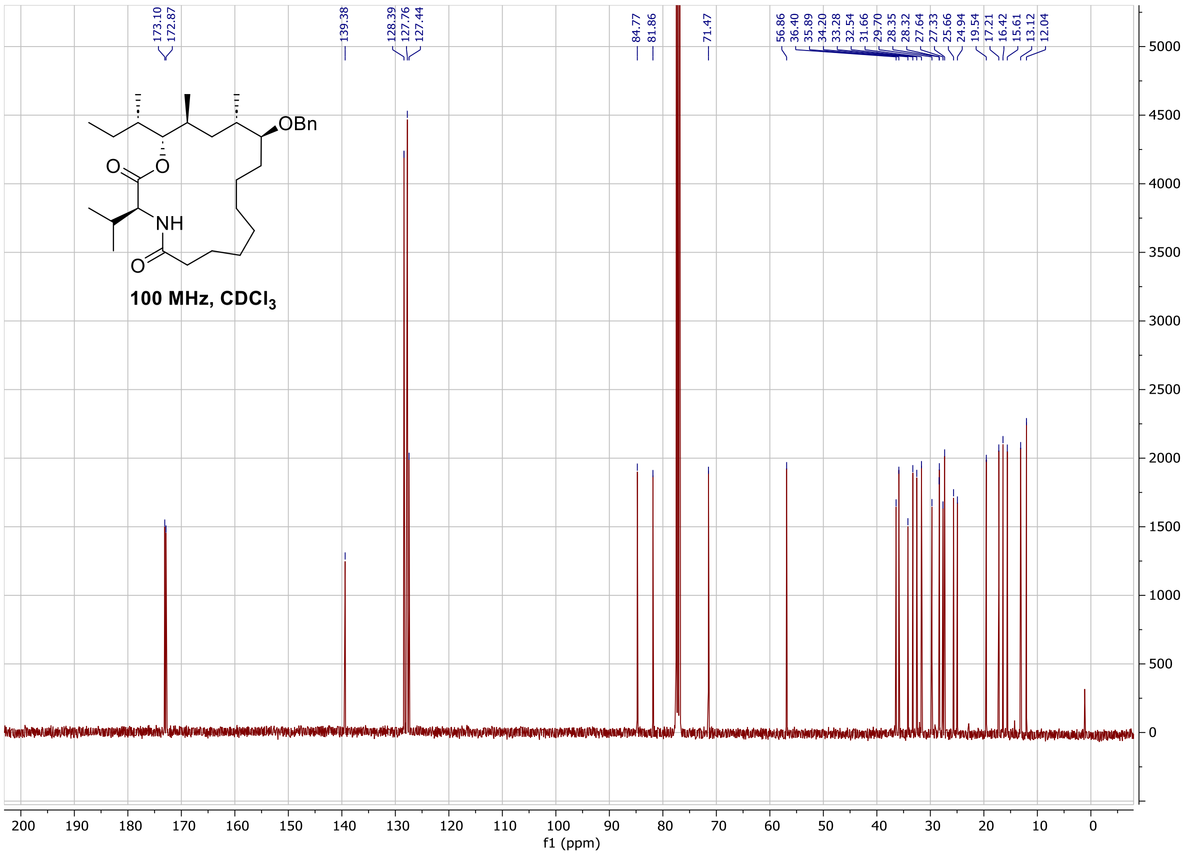

Figure S-44 $^{13}C\{^{1}H\}$ NMR spectrum of compound **20**.

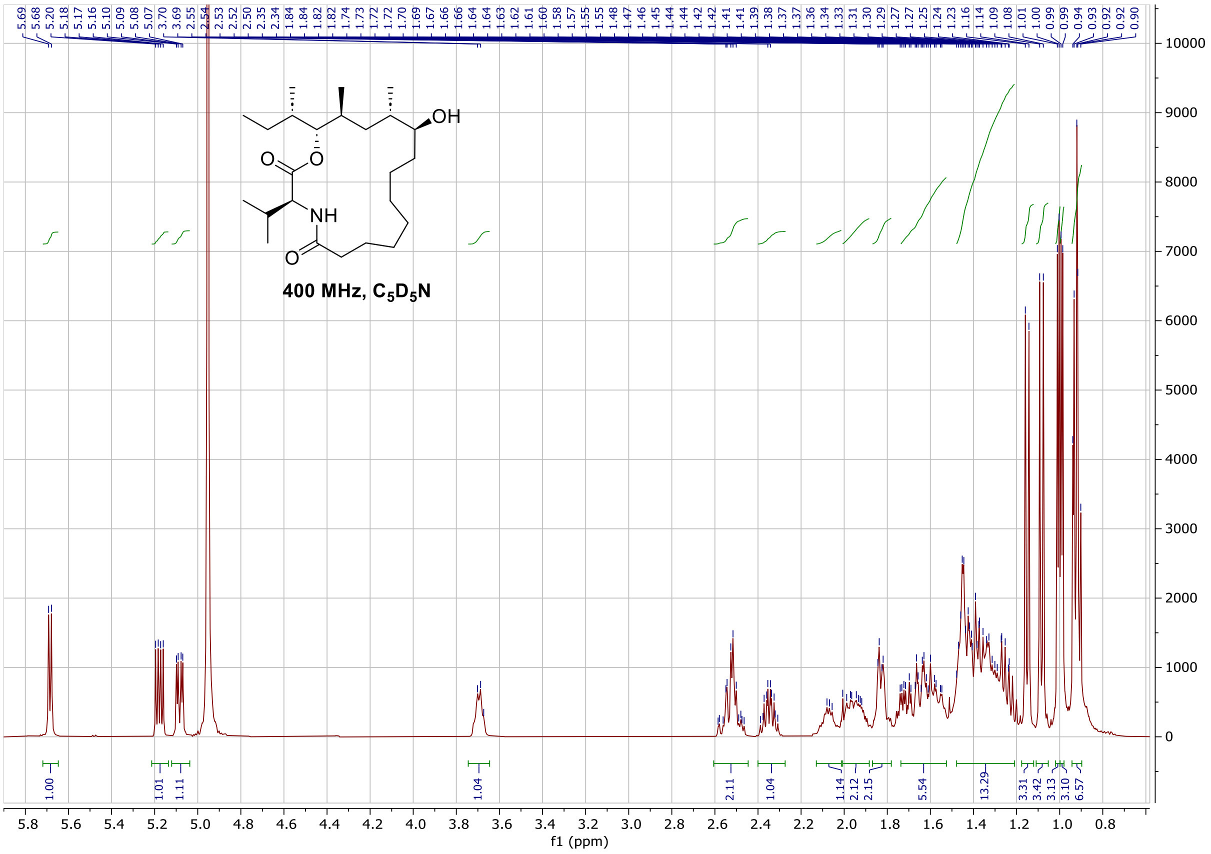

Figure S-45 ^{1}H-NMR spectrum of compound **1** in pyridine-d_5.

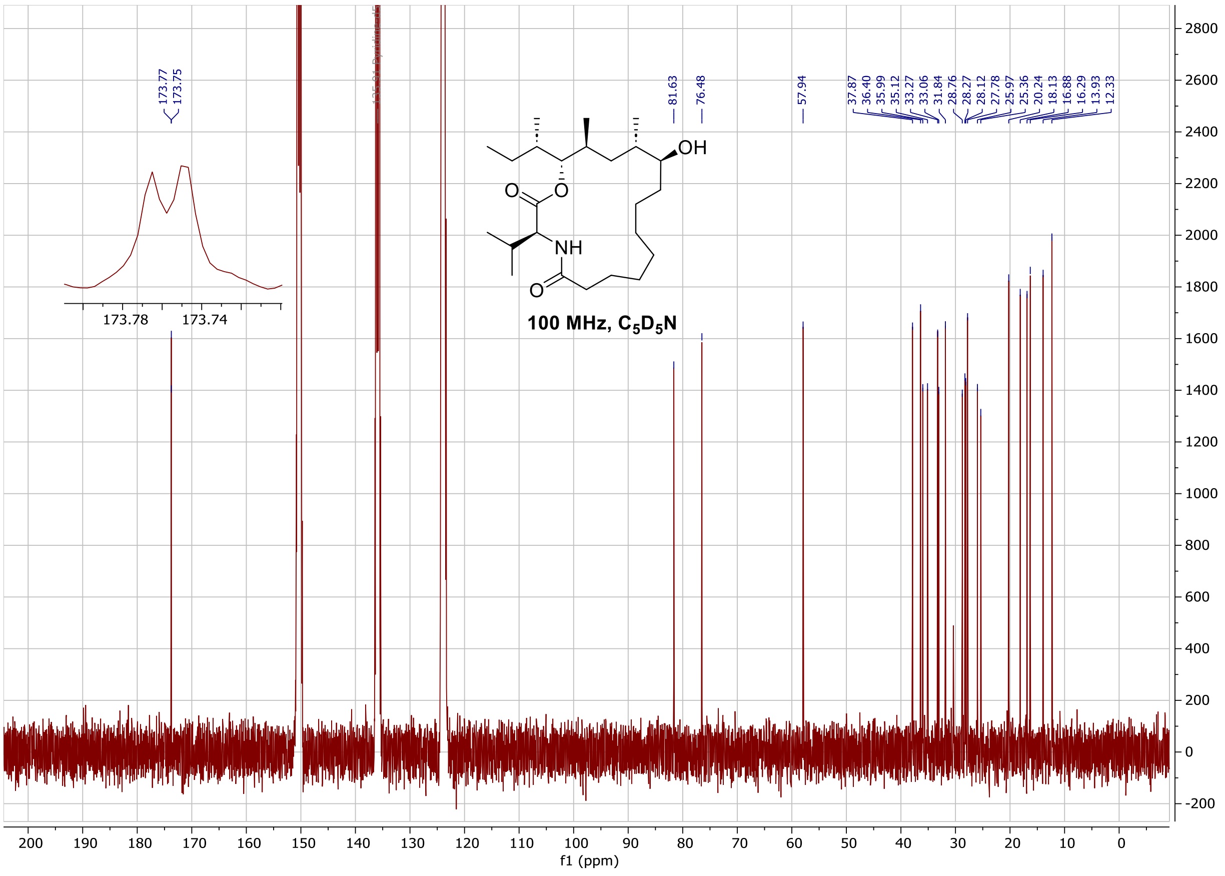

Figure S-46 $^{13}C\{^{1}H\}$ NMR spectrum of compound **1** in pyridine-d_5.

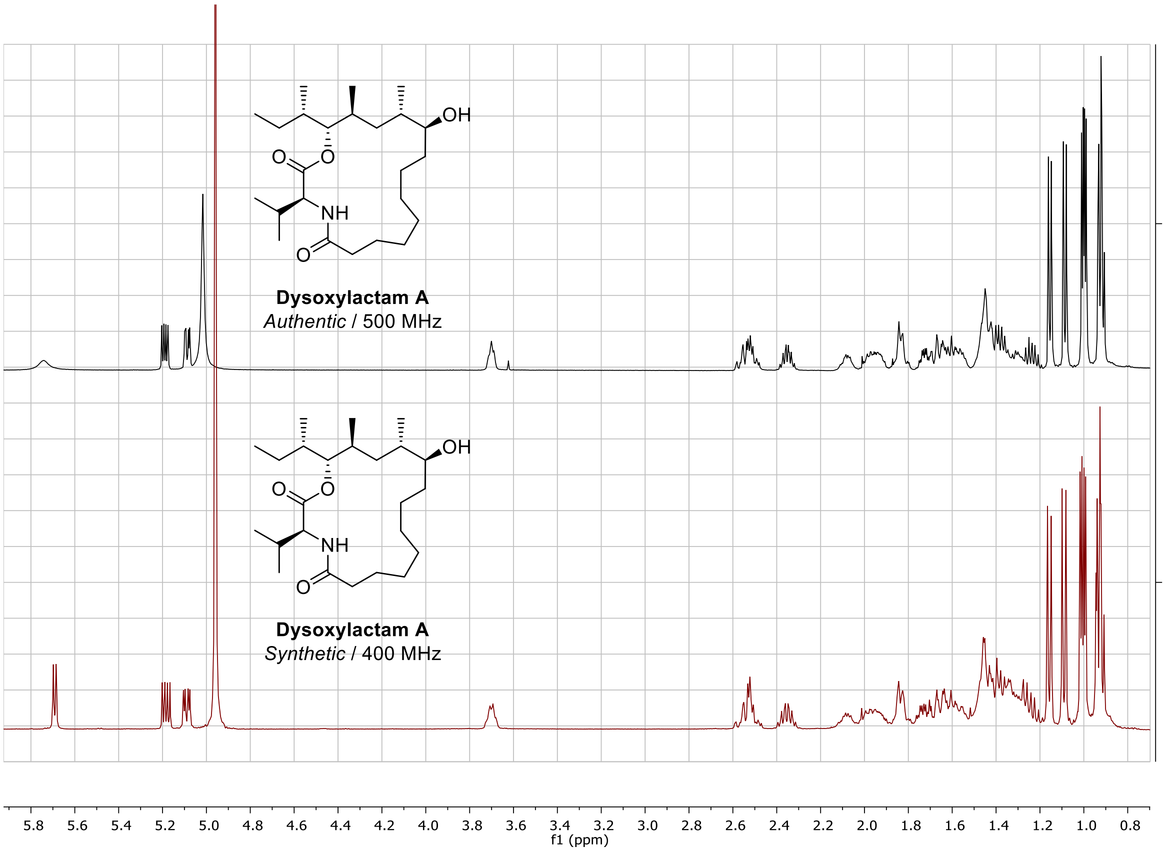

Figure S-47 Direct comparison between the ^{1}H-NMR spectra of synthetic and authentic dysoxylactam A (**1**) in pyridine-d_5.*

---

* The original ^{1}H and ^{13}C NMR data files from the isolation and characterization work were kindly provided to us by Professors Lou and Yue at State Key Laboratory of Drug Research, Shanghai Institute of Materia Medica, Chinese Academy of Sciences, People's Republic of China.

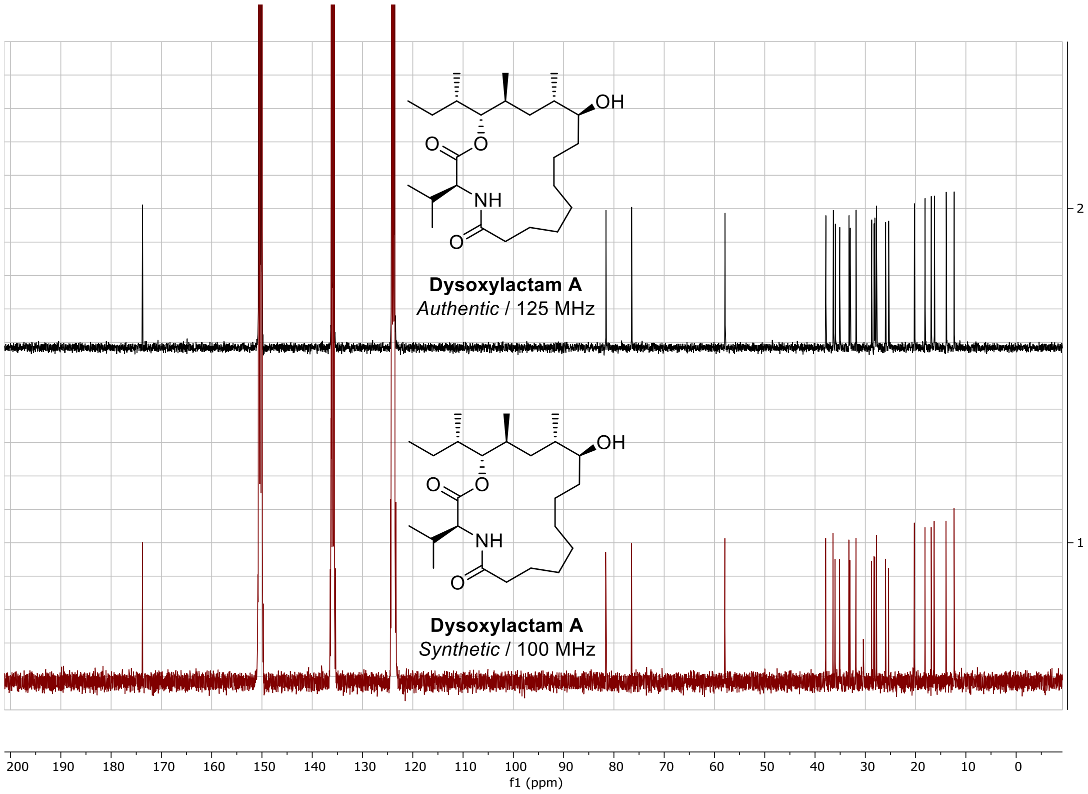

Figure S-47 Direct comparison between the $^{13}C\{^1H\}$ NMR spectra of synthetic and authentic dysoxylactam A (**1**) in pyridine-d_5.

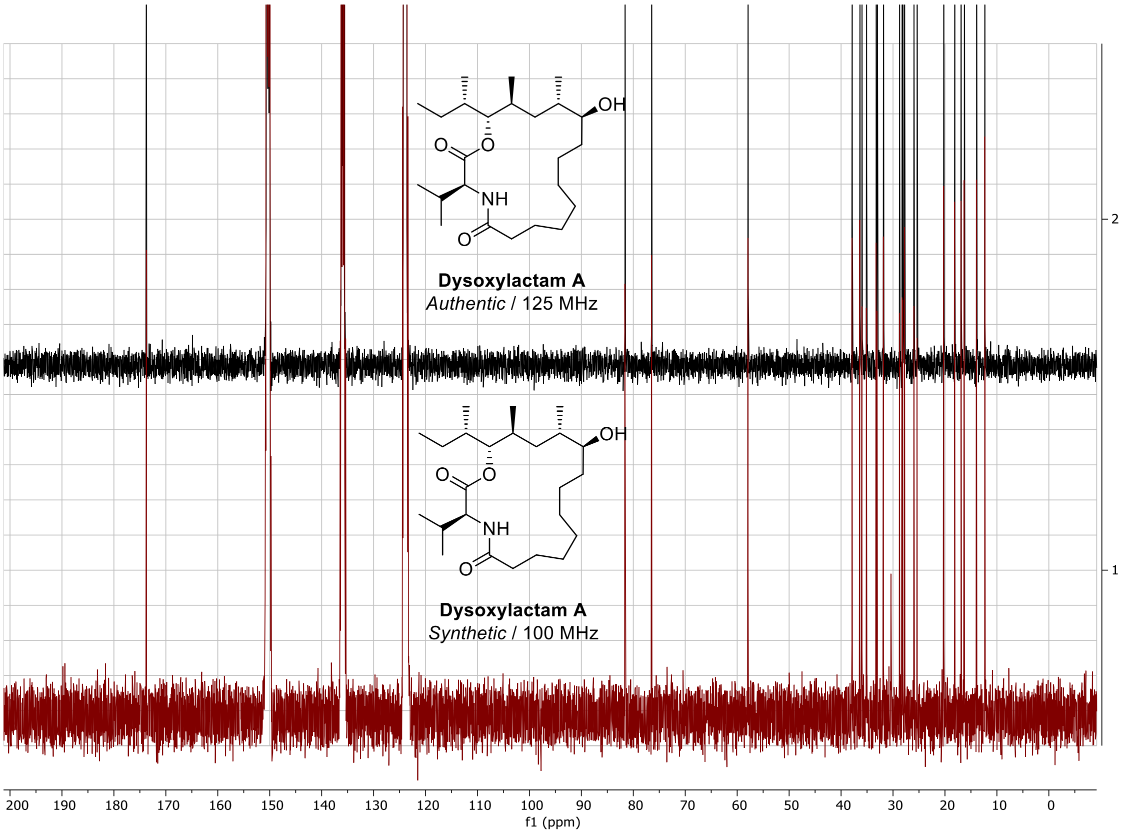

Figure S-48 Direct comparison between the $^{13}C\{^1H\}$ NMR spectra of synthetic and authentic dysoxylactam A (**1**) in pyridine-d_5. *Increased intensity.*

HRMS-spectra

Acquisition Parameter

Source Type	ESI	Set Capillary	3500 V	Set Nebulizer	0.5 Bar
Focus	Not active	Set End Plate Offset	-500 V	Set Dry Heater	200 °C
Scan Begin	50 m/z	Set Charging Voltage	2000 V	Set Dry Gas	4.0 l/min
Scan End	1500 m/z	Set Corona	0 nA	Set Divert Valve	Waste
				Set APCI Heater	0 °C

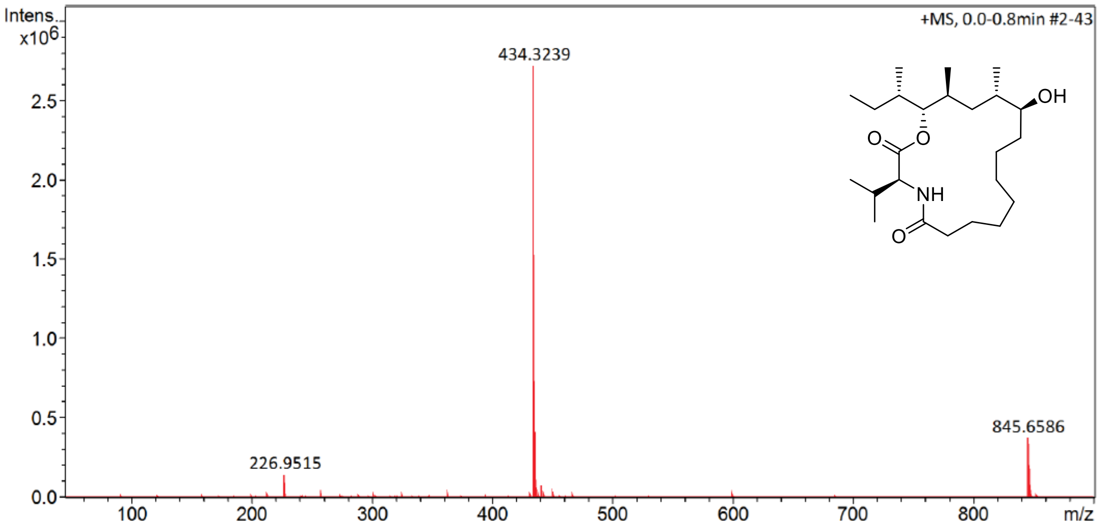

#	m/z	Res.	S/N	I	I %	FWHM
1	90.9766	24974	955.8	17462	0.6	0.0036
2	158.9641	31995	958.1	16842	0.6	0.0050
3	173.0784	32077	705.2	12360	0.5	0.0054
4	198.9566	35337	1224.6	21401	0.8	0.0056
5	212.9722	32998	1620.0	28353	1.0	0.0065
6	226.9515	35079	8087.9	142496	5.2	0.0065
7	256.9620	37580	2698.0	48708	1.8	0.0068
8	273.1672	38000	849.3	15712	0.6	0.0072
9	289.1614	28711	804.4	15442	0.6	0.0101
10	301.1410	39125	1267.1	24941	0.9	0.0077
11	325.2533	37092	1262.6	27386	1.0	0.0088
12	339.0766	37859	597.5	13699	0.5	0.0090
13	362.9263	38593	1181.0	29362	1.1	0.0094
14	394.3314	38255	614.4	16869	0.6	0.0103
15	413.2662	41037	474.9	14147	0.5	0.0101
16	430.9138	41457	738.1	23319	0.9	0.0104
17	434.3239	37970	85100.6	2719282	100.0	0.0114
18	434.4485	63752	1143.8	36549	1.3	0.0068
19	435.3273	37767	22937.5	733683	27.0	0.0115
20	436.3303	38360	3500.7	112178	4.1	0.0114
21	438.3553	41859	1108.9	35642	1.3	0.0105
22	441.2974	39080	2407.8	77918	2.9	0.0113
23	442.3008	39918	701.9	22781	0.8	0.0111
24	450.2979	40161	1221.7	40428	1.5	0.0112
25	466.3503	40815	786.8	27147	1.0	0.0114
26	599.1704	43388	517.5	22108	0.8	0.0138
27	845.6586	42086	7624.8	377163	13.9	0.0201
28	846.6619	41908	4082.8	201853	7.4	0.0202
29	847.6652	42418	1104.6	54594	2.0	0.0200
30	852.6321	44300	307.8	15171	0.6	0.0192

Figure S-49 HRMS of of dysoxylactam A (**1**) with mass spectrum list report.

Acquisition Parameter

Source Type	ESI	Set Capillary	3500 V	Set Nebulizer	0.5 Bar
Focus	Not active	Set End Plate Offset	-500 V	Set Dry Heater	200 °C
Scan Begin	50 m/z	Set Charging Voltage	2000 V	Set Dry Gas	4.0 l/min
Scan End	1500 m/z	Set Corona	0 nA	Set Divert Valve	Waste
				Set APCI Heater	0 °C

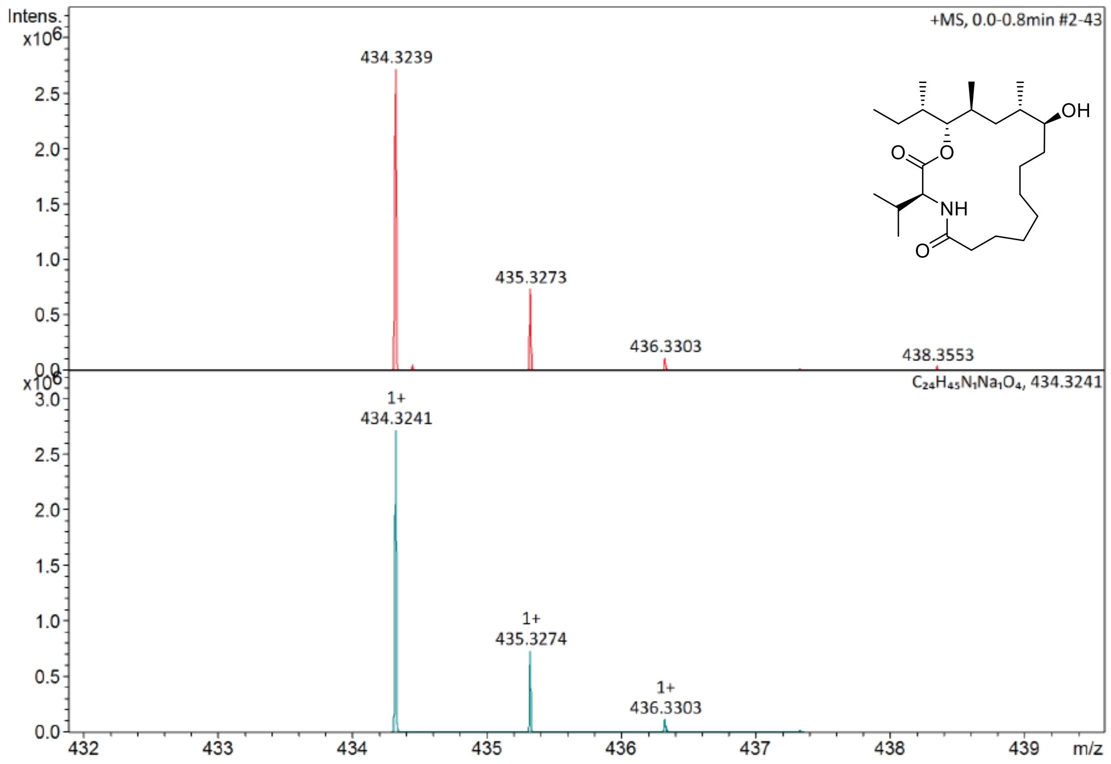

Meas. m/z	Ion Formula	m/z	err [ppm]
434.3239	C24H45NNaO4	434.3241	0.5

Figure S-50 HRMS of of dysoxylactam A (**1**): *Top*: Experimental HRMS spectrum. *Bottom*: Simulated spectrum.†

† We gratefully acknowledge the Mass Spectrometry Laboratory, Department of Chemistry, University of Oslo, for performing the HRMS analysis of the final compound dysoxylactam A (**1**).

References

(1) Paterson, I.; Anne Lister, M. Aldol condensations of chiral ethylketones: Control by chiral boron reagents. *Tetrahedron Letters* **1988**, *29* (5), 585-588.
(2) Freire, F.; Gellman, S. H. Macrocyclic Design Strategies for Small, Stable Parallel β-Sheet Scaffolds. *J. Am. Chem. Soc.* **2009**, *131* (23), 7970-7972.
